# Human archetypal pluripotent stem cells differentiate into trophoblast stem cells via endogenous BMP5/7 induction without transitioning through naive state

**DOI:** 10.1038/s41598-024-53381-w

**Published:** 2024-02-08

**Authors:** Ethan Tietze, Andre Rocha Barbosa, Bruno Araujo, Veronica Euclydes, Bailey Spiegelberg, Hyeon Jin Cho, Yong Kyu Lee, Yanhong Wang, Alejandra McCord, Alan Lorenzetti, Arthur Feltrin, Joyce van de Leemput, Pasquale Di Carlo, Gianluca Ursini, Kynon J. Benjamin, Helena Brentani, Joel E. Kleinman, Thomas M. Hyde, Daniel R. Weinberger, Ronald McKay, Joo Heon Shin, Tomoyo Sawada, Apua C. M. Paquola, Jennifer A. Erwin

**Affiliations:** 1 Lieber Institute for Brain Development, Baltimore, MD USA; 2 Department of Neurology, Johns Hopkins School of Medicine, Baltimore, MD USA; 3 Inter-Institutional Graduate Program on Bioinformatics, University of São Paulo, São Paulo, SP Brazil; 4 Department of Psychiatry, University of Sao Paulo, Medical School, São Paulo, Brazil; 5 Department of Genetic Medicine, Johns Hopkins University School of Medicine, Baltimore, MD USA; 6 Center for Mathematics, Computation and Cognition, Federal University of ABC, Santo André, SP Brazil; 7 Department of Basic Medical Science, Neuroscience, and Sense Organs, University of Bari Aldo Moro, Bari, Italy; 8 Department of Neuroscience, Johns Hopkins School of Medicine, Baltimore, MD USA; 9 Department of Psychiatry & Behavioral Sciences, Johns Hopkins University School of Medicine, Baltimore, MD USA; 10 Present Address: Center for Precision Disease Modeling and Division of Endocrinology, Diabetes and Nutrition, Department of Medicine, University of Maryland School of Medicine, Baltimore, MD 21201 USA

**Keywords:** Induced pluripotent stem cells, Stem-cell research, Transdifferentiation

## Abstract

Primary human trophoblast stem cells (TSCs) and TSCs derived from human pluripotent stem cells (hPSCs) can potentially model placental processes in vitro. Yet, the pluripotent states and factors involved in the differentiation of hPSCs to TSCs remain poorly understood. In this study, we demonstrate that the primed pluripotent state can generate TSCs by activating pathways such as Epidermal Growth Factor (EGF) and Wingless-related integration site (WNT), and by suppressing tumor growth factor beta (TGFβ), histone deacetylases (HDAC), and Rho-associated protein kinase (ROCK) signaling pathways, all without the addition of exogenous Bone morphogenetic protein 4 (BMP4)—a condition we refer to as the TS condition. We characterized this process using temporal single-cell RNA sequencing to compare TS conditions with differentiation protocols involving BMP4 activation alone or BMP4 activation in conjunction with WNT inhibition. The TS condition consistently produced a stable, proliferative cell type that closely mimics first-trimester placental cytotrophoblasts, marked by the activation of endogenous retroviral genes and the absence of amnion expression. This was observed across multiple cell lines, including various primed induced pluripotent stem cell (iPSC) and embryonic stem cell (ESC) lines. Primed-derived TSCs can proliferate for over 30 passages and further specify into multinucleated syncytiotrophoblasts and extravillous trophoblast cells. Our research establishes that the differentiation of primed hPSCs to TSC under TS conditions triggers the induction of *TMSB4X*, *BMP5/7*, *GATA3*, and *TFAP2A* without progressing through a naive state. These findings propose that the primed hPSC state is part of a continuum of potency with the capacity to differentiate into TSCs through multiple routes.

## Introduction

Human models are essential to study human placenta development and function. Comparative genome analysis demonstrates that genes involved in reproduction and placenta function exhibit significant divergence between humans and mice, underscoring the limitations of non-human models^1^. Considering the involvement of placental anomalies in developmental disorders tied to maternal hyperimmune states^2^, in vitro modeling of the human placenta is crucial for pinpointing disease-associated molecular targets, conducting phenotypic screens, and assessing toxicity.

Trophoblast stem cells (TSC) are proliferative in vitro stem cells originating from the trophectoderm layer, with the capacity to differentiate into cytotrophoblasts (CTBs)^3^. These CTBs subsequently develop into syncytiotrophoblasts (STBs) and extravillous cytotrophoblasts (EVTs)^3^. The optimal approach for modeling placental disorders using cohorts of human induced pluripotent stem cells (hiPSCs) remains debated^4^. Patient-derived iPSC cohorts are invaluable for modeling diseases and genetic traits, with iPSC banks typically cultured under primed conditions, establishing primed cells as the starting point for most studies.

Yet, there is ongoing debate regarding the developmental potential of primed PSCs to differentiate into TSCs^4^. Various culture conditions guide human stem cells to different developmental stages, including 'naive' conditions that mimic a pre-implantation epiblast state^5–7^ and archetypical ‘primed’ conditions which achieve a post implantation-like epiblast state. Conventional wisdom holds that the trophoblast lineage, part of the extraembryonic tissues, is specified before the blastocyst stage, suggesting that differentiation of both primed and naive PSCs to trophoblasts is atypical in vivo. Questions remain about the heterogenous pluripotent states of archetypical primed iPSC and the competency of these states to give rise to TSCs^4^. Some studies suggest that primed iPSC are incapable of being specified to TSCs, instead giving rise to amnion-like cells; however, several recent studies conclusively demonstrate that primed iPSCs can generate *bona fide* TSCs by bone morphogenetic protein-4 (BMP4) activation and WNT inhibitor (IWP2) treatment^8,9^ or by inhibiting tumor growth factor beta (TGFβ)^10^. Such conflicting results may stem from the close developmental relationship between human amnion and trophoblasts^11^ and that suboptimal culture conditions^10^.

In this study, we aim to define the developmental trajectory of primed hPSC as they differentiate into TSCs and examine the role of exogenous BMP4 and WNT signaling. Four factors reprogramming of human fibroblasts cultured in either primed or naive conditions leads to upregulation of trophoblast programs, and TSCs can be derived from both naive and primed reprogramming intermediates^12^. Activin/Nodal, fibroblast growth factor (FGF), insulin-like growth factor (IGF), and WNT signaling regulate pluripotency in primed iPSCs, and manipulating these pathways can induce primed iPSC specification to TSCs and other embryonic lineages^13^. BMP4 induces differentiation of human primed iPSCs to trophoblast-like cells^14^. In the presence of BMP4, inhibition of FGF and Activin/Nodal signaling^15,16^ or inhibition of WNT signaling generates more uniform TSCs^8,17^. TSCs can also be generated from human primed iPSCs by the activation of WNT and EGF while inhibiting TGFβ, histone deacetylase (HDAC) and Rho-associated protein kinase (ROCK)^3^, which is enhanced in the presence of BMP4 or by removing WNT activation^10^.

We find that activation of WNT and EGF while inhibiting TGFβ, HDAC and ROCK, in the absence of exogenous BMP4, specifies multiple iPSC lines to TSCs. We demonstrate that TSCs are capable of self-renewal for at least 30 passages and can differentiate into STBs and EVTs. TSCs generated by TS condition are transcriptionally highly similar to TSCs previously generated from primary blastocysts, placenta, and transdifferentiated from either naive or primed conditions. We used temporal single-cell RNA sequencing (scRNA-seq) analysis to elucidate the trajectory of this specification and find that during lineage specification, TSCs upregulate endogenous retroviral genes. Specification employs established trophoblast programs including *TMSB4X*, *YAP*, *GATA3, CDX2* and *TFAP2A* and induces endogenous *BMP5* and *BMP7* expression*,* indicating a role for endogenous BMP signaling even in the absence of exogenous BMP4 treatment.

## Results

### Derivation of proliferative TSCs from primed human PSCs without exogenous BMP4

**Figure 1:**
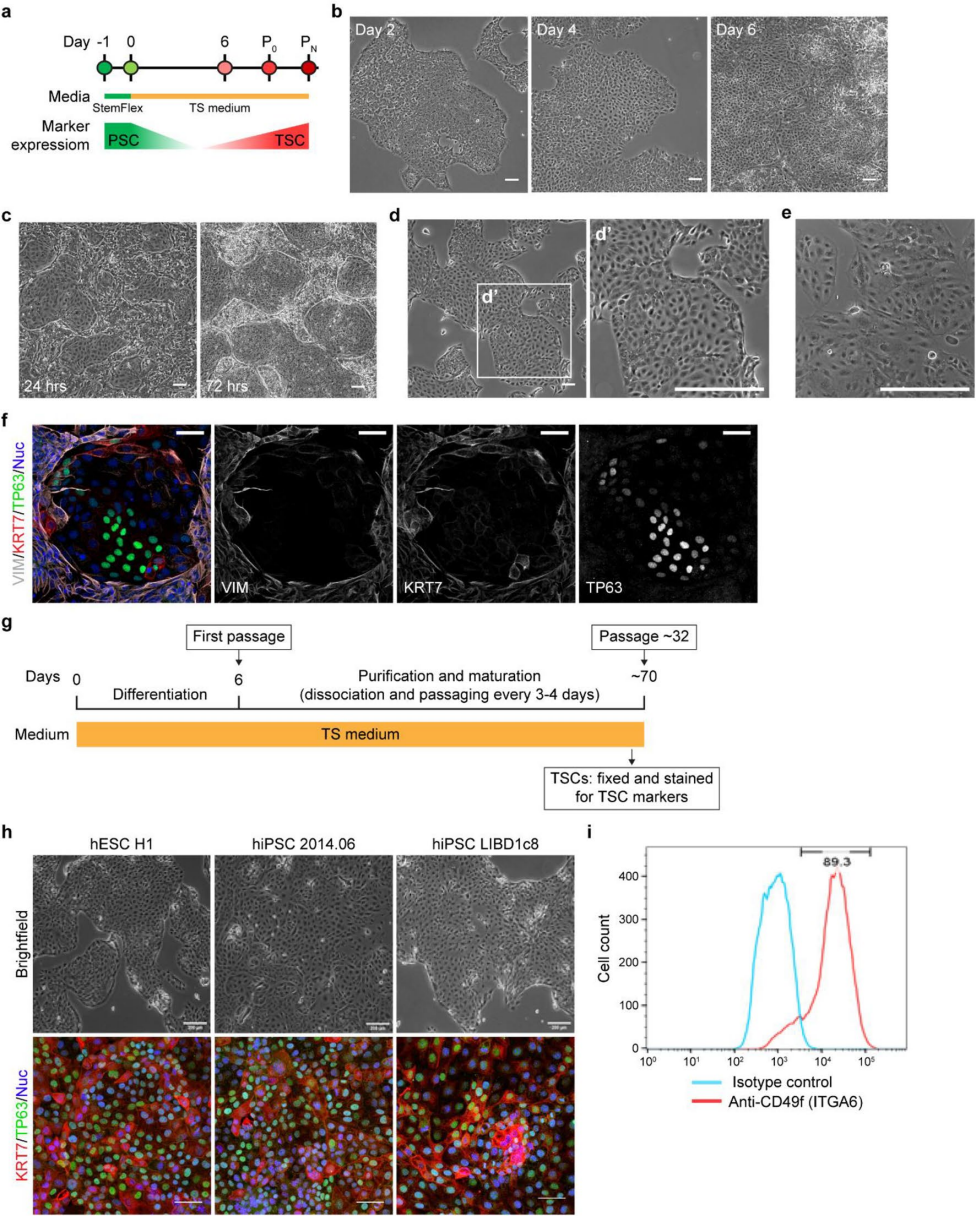
Derivation of trophoblast stem cells (TSCs) from human pluripotent stem cells (hPSCs). (**a**) Schematic overview of TSC derivation from hPSC. (**b**) Representative brightfield images of differentiating TSCs from hESC H1. Days after addition of TS media are indicated. (**c**) Images of TSCs derived from hESC H1 at 24- and 72-h following passage 1 (P1) on day 6. (**d**) Images of mature TSCs derived from hESC H1 after ten passages. Magnified image is shown as d′. (**e**) Image of primary TSCs (CT29)^3^. (**f**) Representative images of immunofluorescence staining of TSCs after one passage for VIM, KRT7 and TP63. Nuclei were stained with Hoechst 33342 (blue). TSCs were derived from hiPSC (LIBD7c6). (**g**) Schematic representation of the protocol for TSC derivation from hPSC. (**h**) Representative bright and immunofluorescent images for TSCs derived from hPSC lines. Nuclei were stained with Hoechst 33342 (blue). (**i**) Flow cytometry data showing the expression of CD49f/ITGA6 in mature TSCs at passage 30 derived from hESC H1. Scale bars, 100 mm (**b**–**e**,**h**: brightfield images); 50 mm (**f**,**h**: immunofluorescent images).

While previously described primed hiPSC differentiations to trophoblast have included BMP4 activation, evidence has shown that hiPSCs can differentiate to trophoblast in the absence of exogenous BMP4^18^. We first asked whether primed human embryonic stem cells (hESCs) and hiPSCs can be differentiated to TSCs in the absence of exogenous BMP4 (Fig. 1a, Supplemental Fig. 1). hESC line H1^19^ colonies demonstrated tight packing with defined edges characteristic of primed cells, 24 h after passaging in feeder-free primed conditions. Once self-organized colonies were established, cells were switched directly to the differentiation medium which activates WNT and EGF while inhibits TGFβ, HDAC and ROCK (TS condition). By brightfield imaging over the subsequent six days, we observed that cells proliferated rapidly and adopted a flatter appearance with some cells adopting a cobblestone appearance (Fig. 1b). After subsequent passaging, two morphologically distinct populations emerged. Circular colonies with an epithelial-like appearance were surrounded by phase bright fibroblastic cells (Fig. 1c). The inner epithelial-like cells continued to proliferate and appeared morphologically similar to TSCs derived from human villus cytotrophoblasts (primary TSCs)^3^ (Fig. 1d,d′,e). Immunostaining revealed that subpopulations of the inner cells expressed TP63 (a CTB marker) and/or KRT7 (a pan-trophoblast marker) and minimally expressed VIM (a stromal marker), while the surrounding fibroblastic cells strongly expressed KRT7 and VIM (Fig. 1f). TSCs were passaged up to 32 times (Fig. 1g) by maintaining the expression of KRT7 and TP63. (Fig. 1h). These TSCs did not express the pluripotency markers, SOX2 and NANOG (Supplemental Fig. 1c and d). The majority of cells at passage 30 highly expressed CD49f./ITGA6 (89.3%), a common cell surface marker for CTBs and human TSCs^3^ (Fig. 1i).

Given that pluripotent stem cell lines vary in their propensity to differentiate to different cell types^20^, we also asked whether TS condition could specify a variety of primed hPSCs from different sources to TSCs. We confirmed TSC specification of a hiPSC line reprogrammed from dermal fibroblasts by Sendai virus, named 2014.06 (Supplemental Fig. 2a–c), and two hiPSC lines derived from postmortem dura fibroblasts by reprogramming with episomal vectors, LIBD1c8 and LIBD7c6^21^ (Supplemental Fig. 1a and b; Supplemental Fig. 2), by immunostaining with VIM, KRT7 and TP63 (Supplemental Fig. 1c and d; Fig. 1f and h).

In summary, our data suggest that primed hPSCs can differentiated to proliferative TSCs in the absence of exogenous BMP4.

### Differentiation of TSCs into multinucleated STBs and EVTs

Proliferative CTBs are bipotential stem cells with the capacity to differentiate into STBs and EVTs^3^. To investigate the differentiation potential of TSCs derived from primed hPSCs, we induced differentiation of TSCs to differentiate into mature trophoblast cell types, EVTs and STBs.

**Figure 2:**
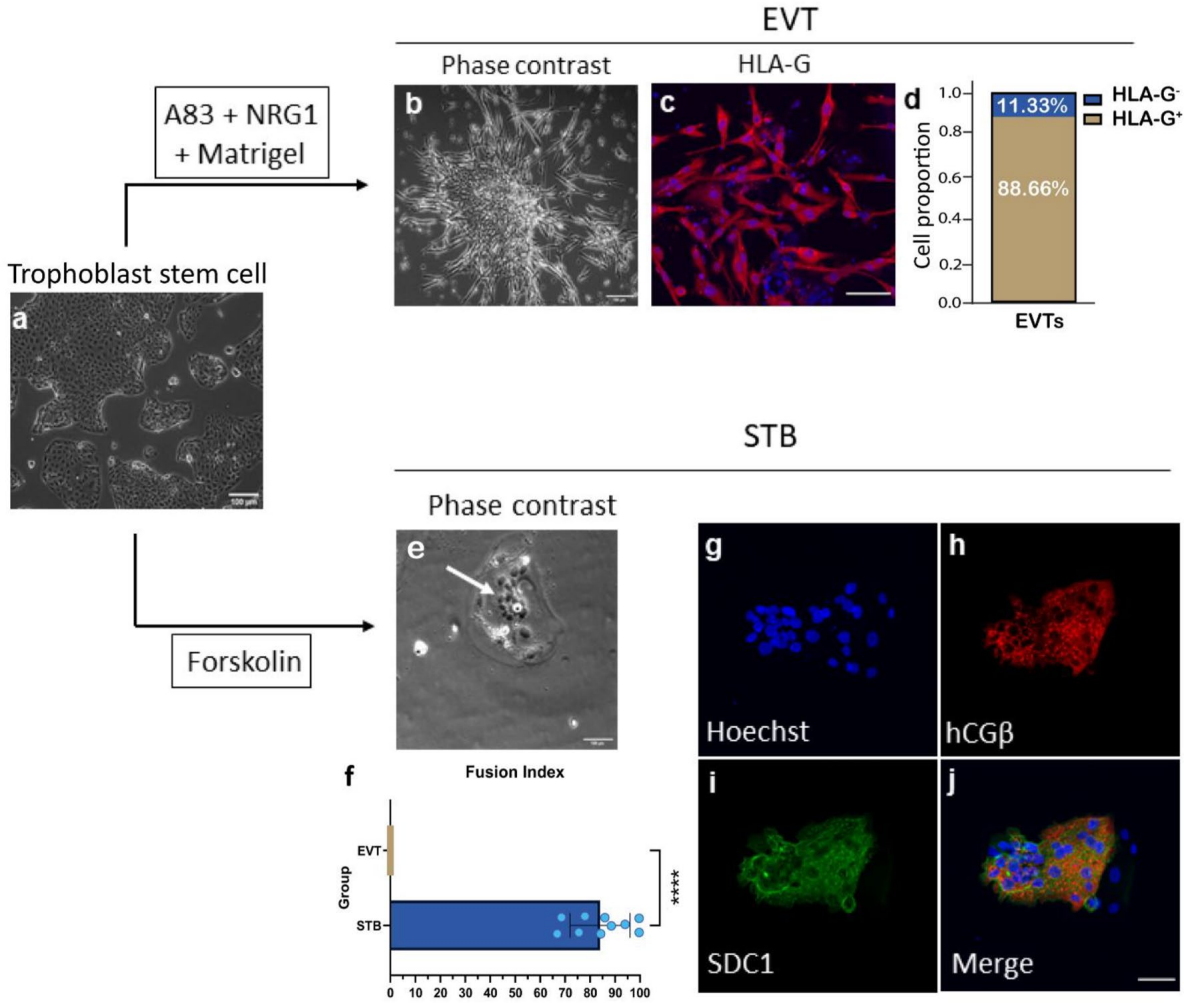
hPSC-derived TSCs differentiate into syncytiotrophoblasts (STBs) and extravillous cytotorophoblasts (EVTs). (**a**) Representative image of TSCs derived from hESC H1. (**b**) Representative image of EVTs differentiated from hESC H1-derived TSCs. (**c**) Immunofluorescence image of EVTs showing the expression of HLA-G. Nuclei were stained with Hoechst 33342. (**d**) Proportion of EVTs positive or negative for HLA-G after 6 days of differentiation (n = 300 cells, 12 ROI). (**e**) Representative image of STBs derived from hESC H1-derived TSCs. (**f**) Fusion efficiency of STBs and EVTs derived from hESC H1-derived TSCs. Ten ROIs for both STBs and EVTs were analyzed; n = 30–50 nuclei per ROI. Data is presented by mean ± SEM. ****p < 0.0001 (Mann–Whitney test). (**g–j**) Immunofluorescence images of STBs showing the expression of hCGb (**h**) and SDC1 (**i**). Scale bars, 200 mm (**a**); 100 mm (**b**,**c**,**e**,**g**–**j**).

EVT differentiation of the hESC H1-derived TSCs (Fig. 2a) was induced with the TGFβ inhibitor, A83, NRG1α and matrigel, as previously described for differentiation of primary TSCs into EVTs^3^. After 9 days in EVT differentiation, TSCs acquired EVT morphology (Fig. 2b), and 88% of cells were positive for the EVT specific marker, HLA-G (Fig. 2c and d), thereby resembling previously described EVTs derived from primary TSCs^3^.

To confirm the differentiation potential into STBs, we induced the differentiation of hESC H1-derived TSCs to STBs with Forskolin for 3 days as previously described^3^. STBs are a terminally differentiated multinucleated epithelial layer that infiltrates the maternal endometrium. The multinucleated cell forms from multiple CTB cell fusions. We observed cells exhibiting the STB morphology of multinucleation (Fig. 2e). The fusion index revealed that more than 80% of the cells were multinucleated (Fig. 2f), similar to STBs derived from human primary TSCs^3^ and TSCs derived from naive hPSCs^22^. These multinucleated cells expressed STB specific markers, human chorionic gonadotropin beta (hCGβ) and Syndecan-1 (SDC1) (Fig. 2g–j), which were not detected in hiPSCs (Supplemental Fig. 1a). Taken together, these findings indicate that primed hPSC-derived TSCs are bipotential stem cells capable of efficient differentiation into both HLA-G-positive EVTs and hCGβ- and SDC1-positive STBs.

### scRNA-seq reveals unique transcriptional programs for primed hiPSCs specification to TSCs in the absence of BMP4

**Figure 3:**
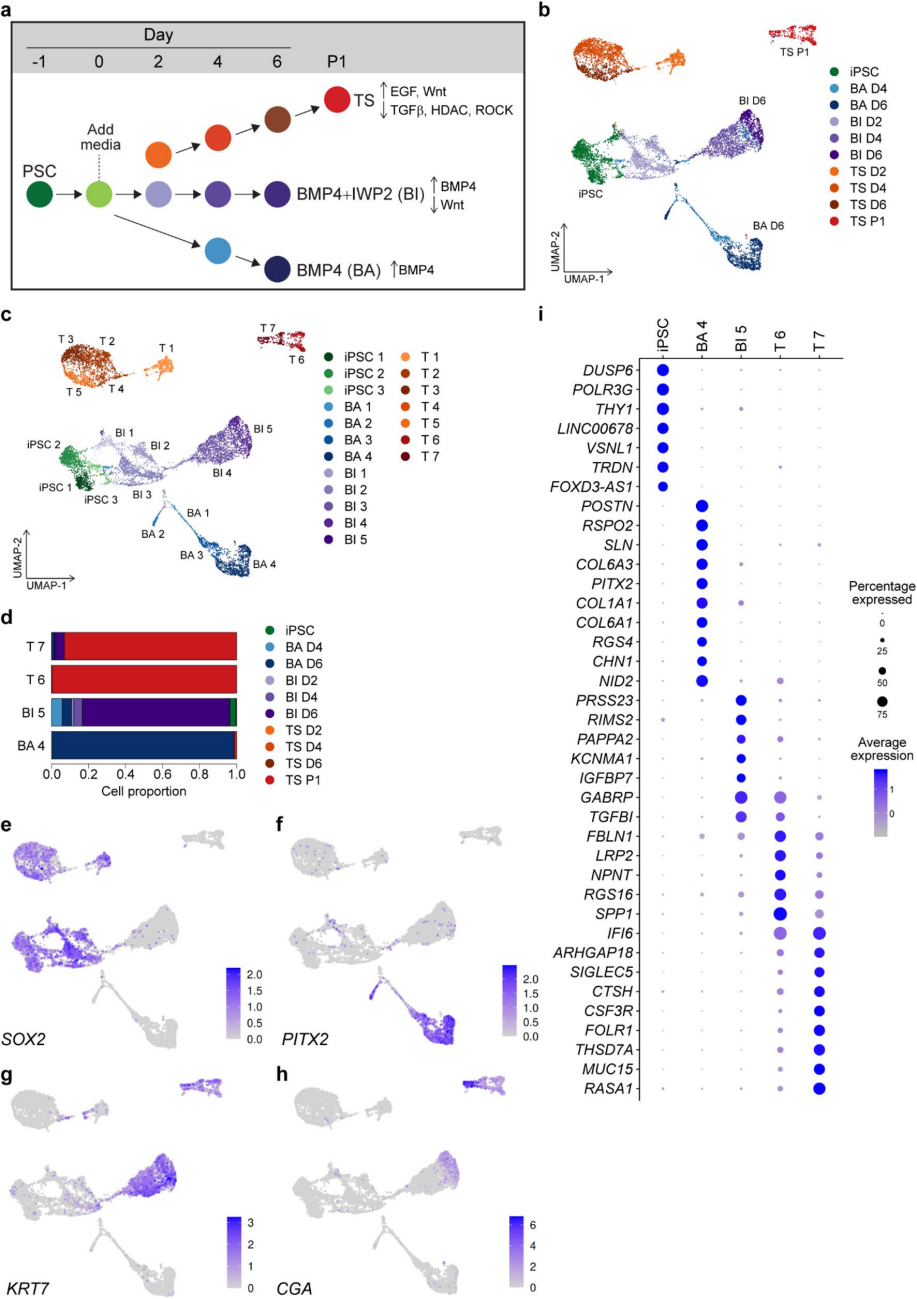
Temporal single cell RNA sequencing (scRNA-seq) of hiPSCs differentiation into trophoblast differentiation. (**a**) Schematic of trophoblast differentiation conditions. hiPSCs were plated and cultivated for 24 h before addition of differentiation media. Cells were collected for scRNA-seq at indicated time points. (**b**) Uniform Manifold Approximation and Projection (UMAP) embedding of 9821 single cell transcriptomes from three distinct differentiation conditions with groupings based on sample identity (**b**) or gene expression clusters (**c**) calculated by k nearest neighbors using the Euclidean distance of the 30 first PCs which identifies 19 clusters. Cells from the BA condition almost exclusively formed four clusters (BA1–BA4) indicated in blue. Cells from the BI condition were found predominantly in a group of five heterogeneous clusters (BI1–BI5) indicated in purple. hiPSCs are indicated in green and cells from TS conditions were found in clusters (T1–T7) indicated in orange. (**d**) Proportion of the cells at the most mature state in each differentiation condition (T7, T6, BI5 and BA4, presented in **c**). (**e**–**h**) UMAP showing the normalized expression of marker genes for iPSCs (*SOX2*, **e**), placental stromal cells (*PITX2*, **f**), trophoblasts (*KRT7*, **g** and *CGA*, **h**). (**i**) Dot plot showing genes upregulated in iPSCs and the most mature cells in each differentiation condition (T7, T6, BI5 and BA4), compared to all other cell clusters presented in (**c**). Non-parametric Wilcoxon rank sum test (adj.*p*-value < 0.05; log_2_FC > 0.25). Average normalized expression levels are indicated.

To define the molecular events involved in the specification of primed hiPSCs to TSCs, we performed temporal scRNA-seq analysis of hiPSCs that were specified into TSCs using the previously described TS condition. Due to previous reports of BMP4 and WNT involvement in this process, we compared specification with TS condition to BMP4 Alone (BA) condition^14^ and BMP4 + IWP2 (BI) condition^17^. Twenty-four hours after passaging of hiPSCs, the medium was changed to differentiation medium for each condition (BA, BI and TS). Single cells were sequenced at the iPSC stage before differentiation (day 0); at days 4 and 6 for the BA condition; at days 2, 4 and 6 for the BI condition, and at days 2, 4, 6 and 8 for the TS condition, resulting in 10 single-cell transcriptomes (Fig. 3a). In the TS condition, day 8 is a timepoint two days after the first passage annotated as passage 1 (P1) in Fig. 3a. In the BA and BI conditions, no proliferative cells survived passaging, therefore the P1 time point was not sequenced. We sought to compare the effects of altering BMP4 and WNT, but did compare previously described iPSC specification methods. All differentiations began with hiPSCs in StemFlex media after clump passaging that gave rise to TSCs from multiple hiPSC lines (Fig. 1), a different iPSC media from previous BMP4 protocols (see Discussion). Using the highly parallel droplet based single cell sequencing method, Drop-Seq^23^, 9821 high-quality cells were obtained after removing cells with less than 1000 genes detected and more than 20% of mitochondrial mapping rate (Supplemental Fig. 3a–d). The average rate of mapping reads for mitochondrial genes in all cells was 3.9%, indicating good viability (Supplemental Fig. 3a). The total number of genes detected ranged between 21,804 at day 4 in the BI condition (BI D4) to 27,134 at day for in the TS condition (TS D4) (Supplemental Fig. 3b). Total number of high-quality cells ranged between 563 (TS D2) to 1572 (iPSC) (Supplemental Fig. 3c).

We performed dimensionality reduction for the most variable genes across all cells with Principal Component Analysis (PCA) and Uniform Manifold Approximation and Projection (UMAP) embedding. Single cells are separated by differentiation stage and condition, with UMAP dimension 2 generally capturing the differentiation condition (Fig. 3b). To identify transcriptionally similar groups of cells (hereafter referred to as clusters), we performed a graph-based clustering analysis of k nearest neighbors using the Euclidean distance of the 30 first PCs in Seurat^24^. As a result, we identified 19 clusters (Fig. 3c). The clusters contained mostly a single time-point and differentiation condition (Fig. 3d; Table S1). The iPSC clusters (iPSC1-3) are primarily composed of iPSCs with minor contributions from the initial days of all three differentiation conditions. Cells from the BA condition almost exclusively formed four clusters (BA1-4). BA1 and BA3 were predominantly composed of cells at day 4 of the differentiation (BA D4), while BA2 and BA4 were predominantly from cells at day 6 (BA D6). Cells from the BI condition were found predominantly in a group of five heterogeneous clusters (BI1-5). BI2 and BI3 clusters were predominantly derived from cells at day 2 of the differentiation (BI D2), while cells at day 4 (BI D4) contributed to BI4 cluster, and cells at day 6 (BI D6) contributed to BI5 cluster. BI1 cluster was mixture of cells in the BI condition at different time points, accompanied by a smaller population from the BA condition. Furthermore, within cluster BI5, there was a minor presence of cells derived from the last day of differentiation under the TS condition (TS P1), indicating the ability of cells in this cluster to differentiate into TSCs. Cells from the TS condition almost exclusively populated 7 clusters (T1–T7). T1 cluster was predominantly composed of cells at day 2 (TS D2) and T2-5 clusters were composed of a mixture of cells at days 2–6 (TS D2, TS D4 and TS D6). The cells from the TS P1 condition separated into two clusters, T6 and T7, providing further support for the brightfield observations indicating the presence of two distinct cell populations (Fig. 1c and f). Small portion of cells at day 6 from the BI condition (BI D6) and the BA condition (BA D6) also contributed to T7 cluster (Fig. 3d; Table S1).

To understand transcriptional changes during the differentiation, we asked which cells express canonical pluripotent, trophoblast and mesoderm specific genes. The PSC marker *SOX2* was highly expressed in the iPSC stage and was absent from the most differentiated clusters in all conditions (BA4, BI5, T6 and T7) (Fig. 3e). *SOX2* expression was maintained at a higher level in the initial days of the TS condition compared to the BI and BA conditions. *PITX2*, identified as a marker gene for a stromal-fetal communicating cell type in scRNA-seq of the human placenta^25,26^, exhibited elevated expression during the later stages of cells under the BA condition (BA2 and BA4). This indicates that the BA condition generates cells with mesenchymal lineage (Fig. 3f). *KRT7* and *CGA* are recognized for their expression in both stem cell-derived CTBs and placental tissue in vivo^27^. We observed the expression of these genes in cells at the later stages of the BI condition (BI4 and BI5) and the TS condition (T6 and T7), indicating a specification towards trophoblast lineage (Fig. 3g and h; Supplemental Fig. 4a and b). Furthermore, cells in both the TS and BI conditions exhibited an overall increase in the expression of trophoblast markers, such as *GATA3* and *TFAP2C* (Supplemental Fig. 4c). Conversely, the BA condition displayed a specification towards mesodermal lineage, characterized by the expression of *GATA4*, *TBXT* and *PDGFRA* (Supplemental Fig. 4d). In the mouse, *Cdx2* is involved in the segregation of the inner cell mass and trophoblast lineages at the blastocyst stage by repressing *Pou5f1*/*Oct-4* and *Nanog* in the trophoblast. Overexpression of *Cdx2* in mouse ESCs causes differentiation to TSCs^28,29^. In humans, *CDX2* expression is initiated after blastocyst formation and has variable expression patterns in trophoblast^30^. Similar to human embryo and primary TSCs^3^, we observed a transient expression of *CDX2* during the intermediate stages of specification (day2–4) for all conditions and is not expressed in the most differentiated state in the TS condition (Supplemental Fig. 4e).

We next investigated differentially expressed genes across the clusters. Primed PSC marker genes, *DUSP6* and *THY1*, are among the top genes upregulated in iPSCs. Cells in the BA4 cluster showed a significant upregulation of genes in WNT signaling pathway, such as a WNT agonist, *RSPO2*^31^ and *PITX2,* which interacts with WNT signaling and regulates collagen expression^32^. Upregulated genes in the BI5 cluster cells included a serine protease involved in Snail-dependent epithelial to mesenchymal transition, *PRSS23*^33^ and insulin-like growth factor signaling *IGFBP7*^34^ and *PAPPA2*^35^. Cells in the T7 cluster exhibited the upregulation of genes regulating EGFR signaling, such as *IFI6,* and *MUC15*^36^, and the YAP downstream effector *ARHGAP18*^37^, which was recently implicated in the specification iPSCs to bipotent TSCs^38^ (Fig. 3i; Tables S2 and S3). To summarize, our comparative analysis of the TSC differentiation conditions reveals that the TS condition—activating WNT and EGF while inhibiting TGFβ, HDAC, and ROCK—elicits transcriptional patterns in primed hiPSCs closely resembling trophoblast cells in vivo.

### Trophoblast expression signatures are enriched in hiPSC-derived TSCs

**Figure 4:**
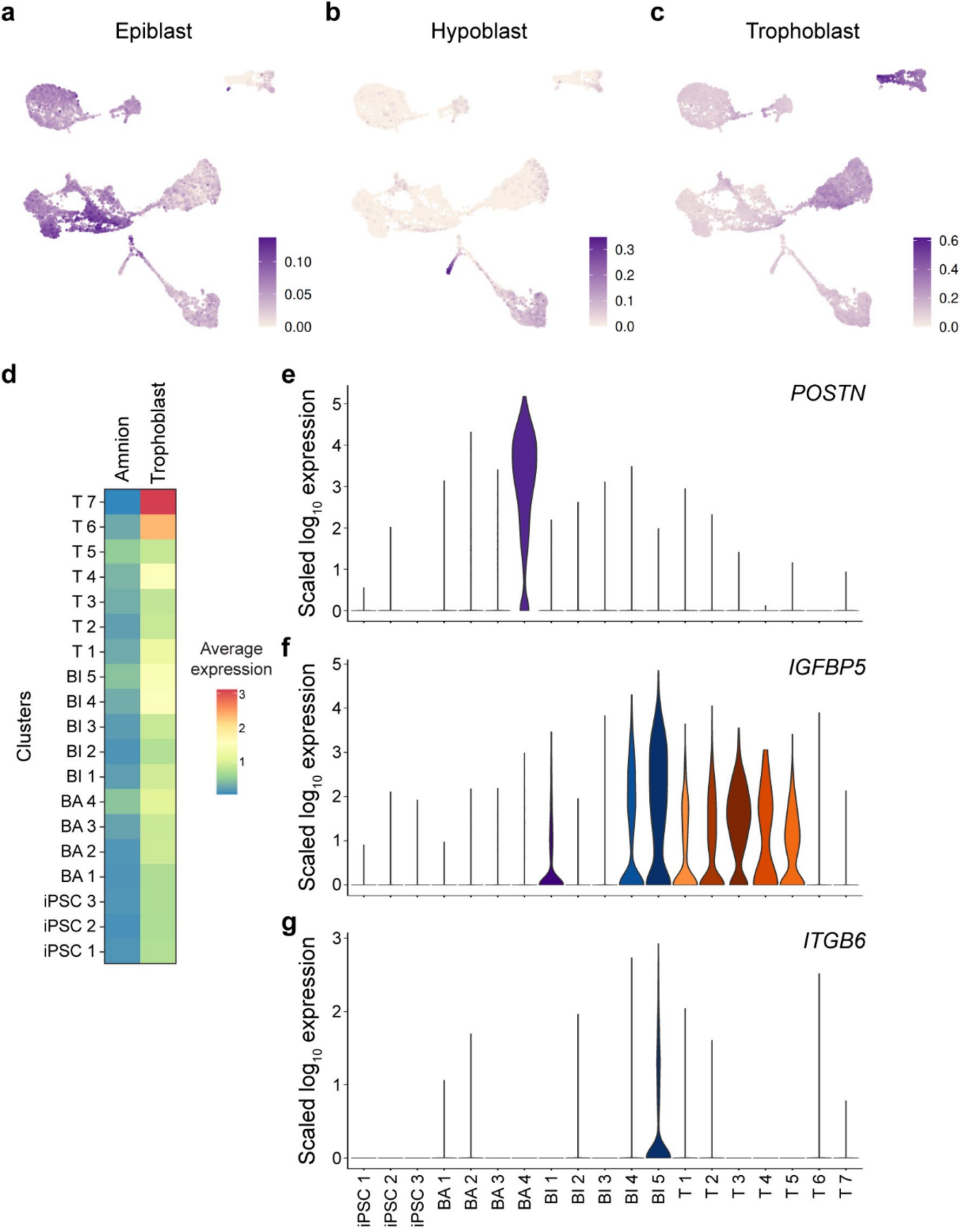
hiPSC-derived TSCs without activating BMP4 exhibit similar gene expression patterns to trophoblasts from cultured human embryos. (**a**–**c**) UMAP showing the expression similarity scores for single cells based on gene expression signatures in epiblast (**a**), hypoblast (**b**) and trophoblast (**c**) of human embryo^39^. (**d**) Mean expression of genes identified in amnion^42^ and trophoblast^39^ in each cell cluster. The T7 cluster shows the highest average expression of trophoblast genes (*p* = 3.5e−41, Wilcoxon Rank Sum Test with Holm-middle combined) and the lowest average expression of amnion genes (*p* = 4.8e−06), compared to all other cell clusters. (**e**–**g**) Violin plots showing the expression of amnion markers *POSTN* (**e**), *IGFBP5* (**f**) and *ITGB6* (**g**) in each cell cluster.

To compare the primed hiPSC-derived cell types from all differentiation condition to peri-implantation human embryos, we calculated the scaled transcriptional similarity of our scRNA-seq data to previously annotated gene signatures for epiblast, hypoblast and trophoblast from cultured human embryos^39^ (Fig. 4a–d). Two iPSC clusters exhibited the highest similarity to epiblast marker gene expression, with average score of 0.1 (Fig. 4a). Interestingly, a population of cells from the BA2 cluster were most similar to hypoblast tissue profiles with a similarity score of 0.3 (Fig. 4b). The T6 and T7 clusters, representing the most mature trophoblast stage, displayed a specific expression profile associated with trophoblast tissue, exhibiting a transcriptional similarity of 0.6 (Fig. 4c). Moreover, the T7 cluster exhibited the highest level of trophoblast gene expression, determined through a pairwise comparison of the average trophoblast gene expression across all clusters (Wilcoxon Rank Sum Test with Holm-middle combined p value = 3.5e−41). Additional validation of the trophoblast transcriptional similarity via gene set enrichment analysis (GSEA) demonstrated that the T7 cluster displayed the most pronounced enrichment for the expression of trophoblast-signature genes (adjusted *p* value = 0.001, Supplemental Fig. 5a). Collectively, the results obtained from our single-cell RNA sequencing analyses suggest that cells derived from hiPSCs, when subjected to the TS condition, develop a trophoblast identity within eight days after specification and passaging.

We further compared the specification map with in vivo amnion. Recent studies suggest that primed PSCs patterned with exogenous BMP4 give rise to amnion-like cells^22,40,41^. Consistent with transcriptional trophoblast identity, the T7 cluster exhibited the highest enrichment for genes identified in in vivo trophoblast^39^. Notably, the T7 cluster had the lowest average expression of genes expressed in in vivo amnion^42^ (Wilcoxon Rank Sum Test with Holm-middle combined *p* value = 4.8e−06) (Fig. 4d; Tables S2–S6). Moreover, we did not observe a significant enrichment of genes expressed in the T7 cluster for human amnion-signature genes (adjusted *p* value = 1, Supplemental Fig. 5b and c). Conversely, cells in the earlier stages of all conditions exhibited expression of the amnion genes.

For example, *POSTN* is significantly upregulated in the BA4 cluster (Wilcoxon Rank Sum test; adjusted p value < 2.225074e−308) (Fig. 4e). *IGFBP5* showed significant upregulation in both the BI and TS conditions, except the most mature clusters T6 and T7 (BI4: adjusted *p* value = 4.389994e−95; BI5: adjusted *p* value = 1.909090e−147; T3: adjusted *p* value = 4.301357e−148) (Fig. 4f). A recent study reported high expression of the amnion gene *ITGB6* in primed hiPSC-derived TSCs^40^. Although clusters from the BI5 cluster showed significant upregulation of this gene (adjusted *p* value = 8.890e−286), it was nearly absent from all cells under the TS condition (Fig. 4g). Taken together, these findings suggest that after eight days of specification and passaging in the TS condition, primed hiPSCs become specified as trophoblasts without expressing the amnion genes.

### Primed hiPSC-derived TSCs resemble first trimester placental CTBs

Next, we explored the similarity between primed hPSC-derived TSCs and cells from human early placentas. By GSEA using Cell-Specific Expression Analysis (CSEA)^43^, we identified genes that were preferentially expressed in each of the 19 clusters of hiPSC-derived cells (Fig. 3c), with a specificity index probability (pSI) statistic at thresholds of *p* < 0.05. We then tested whether cell type-specific genes previously identified by scRNA-seq of human placental^25,26^ are over-represented in the cell type-specific genes for the 19 clusters, by hypergeometric test and applied the Bonferroni correction for multiple comparisons, considering all the tested gene lists [α = 0.05/(19 × (38 + 14)) = 5.1 × 10^–5^].

**Figure 5:**
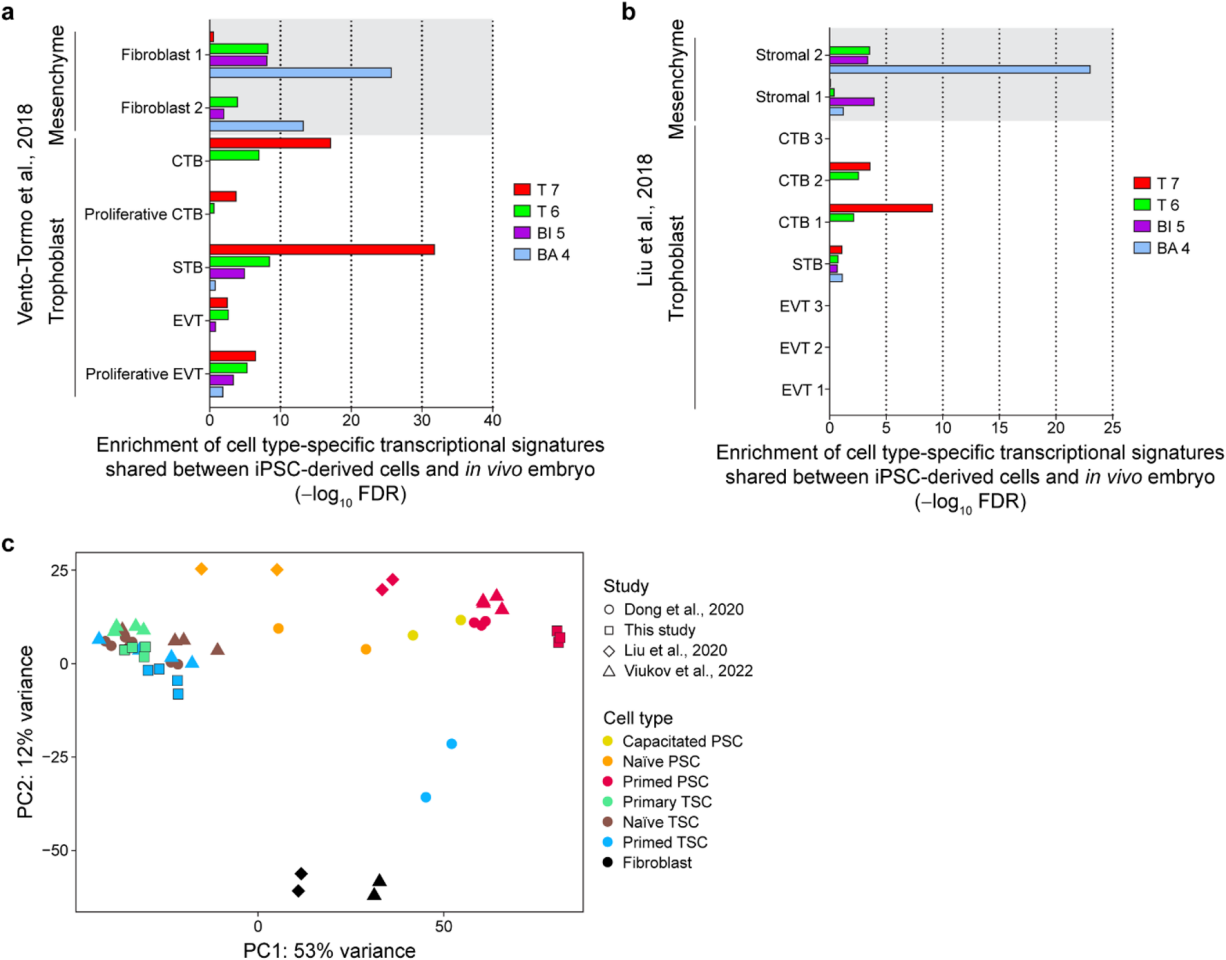
hiPSC-derived TSCs exhibit similarity to human placental cells during the first trimester. (**a**,**b**) Gene set enrichment analysis (GSEA) of cells at the most mature state in each differentiation condition (T7, T6, BI5 and BA4) to cell types identified in first trimester placenta^25,26^. (**c**) PCA plot showing the similarity of TSCs derived from hPSCs (primed TSC) in this study to TSCs from human primary tissues (primary TSC)^3,10^, TSCs derived from naive hPSCs (naive TSC)^10,41^ and primed TSCs in the previous studies^10^.

The transcriptional profiles from all iPSC clusters (iPSC1-3) and clusters from the earlier timepoints (days 2 and 4) in all differentiation conditions did not show significant enrichment for any of the placental cell type clusters from first-trimester placentas^26^ (Supplemental Fig. 6a and b). The T6 and T7 clusters, which are composed of cell at the most mature state in the TS condition, were significantly enriched for the STB and CTB clusters from first-trimester placentas^26^ (Fig. 5a and Supplemental Fig. 6a). The expression profile of the T7 cluster was highly specific for STB and CTB with no significant enrichment for any other cell-type in the maternal fetal interface. To visualize placental cell-type specific expression patterns among the hiPSC-derived cell types, we generated a heatmap of the top unique markers of the fetal cell types identified in Vento-Tormo et al.^26^ and polar trophectoderm markers identified in Petropoulos et al.^44^ (Supplemental Fig. 6c). Consistent with the GESA, we observed high expression of a majority of the unique marker genes for CTBs and STBs in the T7 cluster. Conversely, we observed the significant enrichment of the BA4 cluster for two fibroblast cell types in first-trimester placentas. Vento-Tormo et al. described that these placental fibroblasts as mesenchymal stromal cells of fetal origin that derive from the primitive endoderm expressing *GATA4*, *GATA6*, *PDGFRA*, and *SOX17* (Supplemental Fig. 7a). The BI5 cluster did not show strong enrichment for a specific placental cell type and instead had weaker enrichment for several clusters including STBs, and maternal decidua derived fibroblast endodermal cell type and decidual stromal cells (Fig. 5a and Supplemental Fig. 6a). We also compared the expression profiles of the 19 clusters to cells from first trimester (8 weeks of gestation) and second trimester (24 weeks of gestation)^25^ (Fig. 5b and Supplemental Fig. 6b). Consistent with the findings in Fig. 5a, we observed a strong enrichment of the T6 and T7 clusters for the fusion competent CTBs (CTB1-3). The BA and BI clusters were enriched for two different mesoderm cells (Stromal 1 and 2). In summary, TSCs derived from primed hiPSCs are highly enriched for the expression of genes specific to STBs and CTBs from human early placentas.

**Figure 6:**
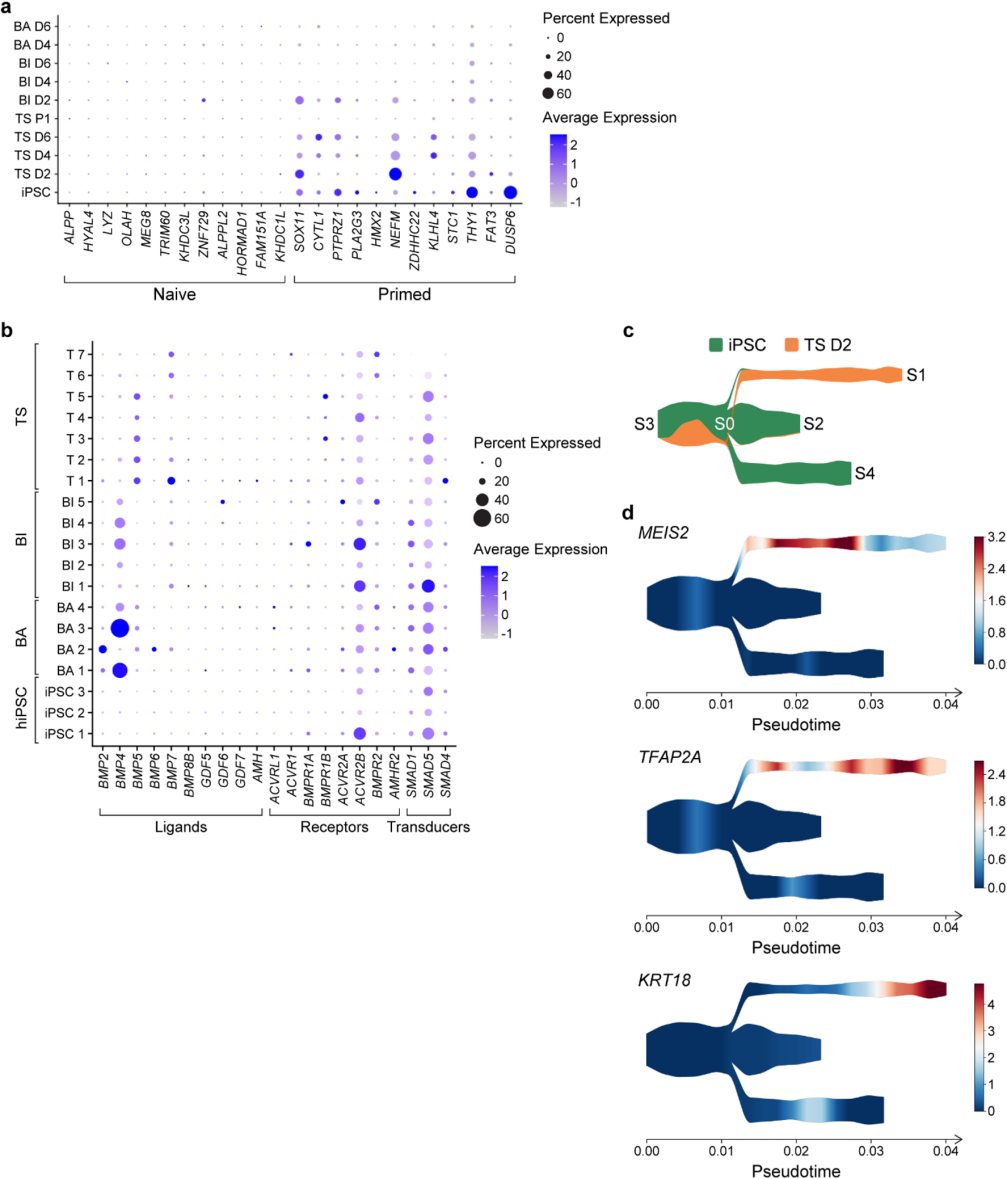
The process of primed hiPSC specification into TSCs begins with *TFAP2A* rewiring, without activating programs associated with naive hPSCs. (**a**) Dot plot showing the expression of marker genes for naive and primed hPSCs in cells derived from each differentiation condition across various time points. (**b**) Dot plot displaying the expression of genes in BMP signaling in each cell cluster. (**c**) Developmental trajectory from hiPSCs to cells differentiated in TS condition at day 2 (TS D2). Cell proportion along the smoothed pseudotime is shown. Nodes are labeled with numbers S0–S4. Branches are defined as the cells between 2 nodes. (**d**) Expression of top ranked genes showing the significant correlation with the pseudotime transition from S0 to S1 (TSC lineage).

Additionally, we compared the expression profiles of the 19 clusters of hiPSC-derived cells to lineage and marker genes from pre-implantation datasets^45^. We found the highest expression of trophoblast genes such as *KRT18*, *TEAD3*, *GATA3* and *GATA2* in the TS clusters (T6 and T7), with moderate expression in BI clusters (BI4 and BI5) (Supplemental Fig. 7a). Primitive endoderm genes such as *SOX17*, *GATA4*, *GATA6* showed the highest expression in the BA clusters. When compared to primary trophoblast cells, we confirmed that the T7 cluster has the highest-level expression of CTB, TSC and EVT enriched genes (Supplemental Fig. 7b).

We then sought to evaluate the biological similarity of primed hPSC-derived TSCs, specifically those differentiated under the TS condition, and several other types of TSCs obtained from different sources. These included primary TSCs from human blastocysts and early-stage placentas^3^ as well as TSCs derived from both naive and primed hPSCs in the previous studies^10,12,41^. Principal component analysis of bulk RNA-seq data illustrated that TSCs obtained from primed hPSCs under the TS condition display a comparable transcriptional profile to TSCs from human primary tissues, as well as those derived from both naive and primed PSCs (Fig. 5c).

Altogether, TSCs obtained from primed hPSCs under the TS condition exhibit a transcriptional profile, which is highly similar to CTBs from human early placenta. These cells are transcriptionally indistinguishable from TSCs derived from human blastocysts and early-stage placentas and from both naive and primed PSCs. Our data also suggest that hPSC-derived cells differentiated under the BA condition exhibit more heterogeneous placental cells types with evidence of the amnion gene expression (Fig. 4).

### Specification of primed hiPSC to TSC initiates with *TFAP2A* rewiring without activation of naive hPSC programs

It has been reported that primed hPSCs are restricted in their potency and unable to differentiate to TSCs, while naive PSCs readily differentiate to TSCs^41^. Therefore, we asked whether the primed hiPSC or cells differentiating from these cells under the BA, BI and TS condition adopt a naive stem cell program during differentiation. Across the differentiation condition, expression of marker genes of the naive PSCs^46^ was nearly absent (Fig. 6a). Especially, cells from any conditions did not express *HORMAD1*, *ALPPL2*, *KHDC3L*, *TRIM60*, and *HYAL4* (Fig. 6a, Table S7). Less than 1% of cells from any condition showed detectable expression for six other marker genes *ALPP*, *OLAH*, *LYZ*, *MEG8*, *KHDC1L*, and *FAM151A*. *ZNF729*. In contrast, marker genes of primed PSCs were consistently expressed in iPSCs and often throughout the differentiation state. These findings suggest that the transition of primed hiPSCs into TSCs does not necessitate the activation of transcriptional programs typically associated with naive hPSCs.

Next we investigated the contribution of BMP signaling to the TSC differentiation processes from primed hiPSCs. We observed that only a small proportion of cells expressed *BMP4* under the TS conditions, whereas its expression was notably increased in various clusters from the BA and BI conditions compared to iPSCs and cells originating from the TS condition (Fig. 6b; Table S7). Conversely, *BMP5* and *BMP7* were expressed in a larger portion of the cells under TS condition compared to iPSCs (*BMP5*: 0–0.67% iPSC vs 24.72% T1 cells and *BMP7*: 1.01–4.71% iPSC vs 29.06% T1 cells). Genes encoding the BMP effector proteins (transducers) *SMAD1*,*4*, and *5* are expressed during the earlier days in all conditions, which may indicate that the endogenous BMP signaling is important during the initial differentiation process.

To identify the most significant gene expression changes associated with the initial stages of hiPSCs specification to TSCs under the TS condition, we applied a single-cell trajectory inference and pseudotime estimation (STREAM)^47^ to iPSC and cells at day 2 under the TS condition (TS D2). Briefly, single cells were ordered along probabilistic trajectories and a numeric value referred to as pseudotime was assigned to each cell to indicate how far it progresses along the dynamic differentiation.

STREAM identified an initial branch composed of both iPSCs and TS D2 cells (S3-S0), which transition to three branches: S0-S1 branch reflecting the exit from pluripotent state and specification to TSC lineage and two branches reflecting the pluripotency continuum (S0–S2, S0–S4) (Fig. 6c,d; Supplemental Fig. 8a and b; Tables S8–12). We next identified the genes involved in the specification that are correlated with the transition along the branches (see “Material and methods”). The pluripotency to TSC lineage pathway (branch S0-S1) showed an upregulation of genes encoding cytokeratin (*KRT18*, *KRT19* and *KRT8*) and calcium binding proteins, such as *S100A11* and *S100A10*, which are known to be expressed in trophoblast^48,49^ (Supplemental Fig. 8c). The top positively correlated transition genes along the branch S0-S1 included *LRRN3*, which amplifies MAPK signaling through *EGFR*^50^, *MEIS2*^51^, *BMP5*, *BMPR1B* and *TFAP2A* (Fig. 6c,d) a transcription factor key to the suppression of pluripotency and expression of trophoblast associated genes^52^. We also found *TMSB4X* as a top transition gene, which is implicated in stemness of progenitor trophoblast cells of first trimester human placentas by increasing NOTCH1 activity^53–55^ (Tables S8–12). The top positively correlated transition genes along the branches S3-S0, S0-S2 and S0-S4 included known primed PSC markers such as *THY1*, *LINC00458* and *SOX11* (Supplemental Fig. 8d). Collectively, these findings suggest that the activation of trophoblast transcriptional programming takes place early in the hiPSC specification process to TSCs, facilitated by known trophoblast-associated regulatory factors such as *TMSB4X*, *YAP*, *BMP5*, and *TFAP2A*.

### Human endogenous retrovirus-derived genes participate in a regulatory subnetwork within TSCs derived from primed hiPSCs

Endogenous retroviruses have been pivotal in the evolutionary diversification of the mammalian placenta, with numerous instances of human endogenous retroviruses (hERVs) being co-opted for essential functions^56–59^. We reasoned that hERV expression may be altered because primed hPSCs, naive hPSCs, and TSCs differ in the regulation of human endogenous retroviruses^60^. Therefore, we investigated how hERVs are expressed during the specification. While there are potentially 1,500 ERV-derived genes capable of encoding proteins^57^, we restrict our analysis to the 20 ERV-derived genes that are currently annotated as human genes including *ERV3-1, Suppressyn (SUPYN/ERVH48-1)*, *Syncytin-1 (ERVW-1)* and *Syncytin-2 (ERVFRD-1),* which function in antiviral responses and syncytial fusion. We found specific upregulation of five ERV-derived genes, *ERVH48-1*, *ERV3-1*, *ERVMER34-1*, *ERVW-1* and *ERVFRD-1*, in the most differentiated cells in the TS condition, cluster T7 (Supplemental Fig. 9a).

To understand the place of ERV-derived genes in the TSC regulatory network, we analyzed the participation of ERV-derived genes in a gene regulatory network. We built a transcription factor (TF) and target gene network model using the Passing Attributes between Networks for Data Assimilation (PANDA) algorithm on all clusters among the different specification conditions. PANDA integrates information from TF-sequence-motif data, gene expression and protein–protein interaction (PPI) in a message-passing approach^61^. We found that ERV interactions (TFs + ERV-derived genes) are prominent in the most differentiated TS cluster (TS 7) and in no other cell clusters. We found regulatory interactions for five ERV-derived genes (*ERVH48-1*, *ERV3-1*, *ERVMER34-1*, *ERVW-1* and *ERVFRD-1*) are highly unique to the TS 7 cluster network, while only 0 or 1 ERV-derived gene was found in the other cell clusters (Supplemental Figs. 9b and 10). Interestingly, genes encompassed in ERV regulatory interactions in TSCs were enriched in biological pathways related to hormone metabolism, cell differentiation and the immune system (adj. p value < 0.01) (Supplemental Fig. 9c; Table S13). In addition, ERV regulatory interactions are also enriched for categories such as placenta development, trophoblast cell differentiation and syncytium formation (Table S13). To understand the significance of the ERV-subnetwork interactions, we next evaluated the strength of ERV edges in the TS cluster network. Weights of ERV regulatory interactions were highly ranked among all network connections (Wilcoxon Rank Sum *p*-value = 1.6778e−31, permuted *p*-value = 0.001), indicating a significant contribution to the gene regulatory network of the TSC state. In sum, we found that ERV-derived genes participate in a regulatory subnetwork within TSCs derived from primed hiPSCs.

## Discussion

In this study, we delineate how primed human induced pluripotent stem cells (hiPSCs) are specified into trophoblast stem cells (TSCs) through the exogenous application of Epidermal Growth Factor (EGF), WNT, and valproic acid, coupled with the inhibition of Tumor Growth Factor Beta (TGFβ) and histone deacetylases (HDAC). We further demonstrate the involvment of *GATA3*, *BMP5/7*, and *TFAP2A* in this process, which notably occurs without passing through a naive state. We demonstrate that TSCs derived from primed hiPSCs closely mirror the transcriptional profile of cytotrophoblasts (CTBs) present in the human placenta. These TSCs exhibit rapid proliferation and can differentiate into extravillous trophoblasts (EVTs) and multinucleated syncytiotrophoblasts (STBs), akin to hTSCs. Within eight days of induction, the TSCs manifest a distinctive and active transcriptional network of human endogenous retroviruses, paralleling that found in in vivo CTBs.

The ability of primed human stem cells to differentiate into TSCs and trophoblast-like cells have been contested. Here we confirm that primed hiPSC retains a broad potency to generate TSCs which are transcriptionally highly similar to primary TSCs. While recent studies conclusively prove that primed hiPSC can be specified to TSCs^62,63^, studies report absent^64^ or inefficient conversion to TSCs with the direct application of the TSC-media, as described here. Optimized culturing conditions and differences in the primed hiPSC state at the initiation of TSC-media exposure likely explain the more robust differentiation described here. We induce differentiation after self-organized multicellular colonies are established by clump-passaging primed cells from StemFlex conditions, plating cells in StemFlex media and inducing differentiation 24 h after passaging all in 20% O_2_. Instead of StemFlex, others have initiated differentiation with primed hPSCs cultured on MEFs^10^, TSER or E8, a xeno-free media, and used either 20% or 5% O_2_. StemFlex is proprietary media containing bovine serum albumin and not all components are known. While PSCs grown in StemFlex and E8 have similar transcriptional profiles, hPSC in StemFlex demonstrate less karyotypic abnormalities, more ubiquitous expression of Nanog, and functional differences such as enhanced single-cell cloning and robustness to automation^65^. While the conditions optimized here improve the specification of TS from primed hiPSC, removing WNT for the initial days would likely improve the efficiency even further, as recently published for primed hiPSC grown on MEFs^10^ although not directly tested here.

We also confirm that the exogenous BMP4 protocols can generate cells with key features of trophoblast-like cells, but the majority of cells specified by BMP4 are less similar to in vivo CTBs. Importantly, the BMP4 Alone methods (BA condition) used here differ from the more established BAP method^15,16^, which are optimized to produce more efficient trophoblast differentiation. For the direct TSC differentiation method here (the TS condition), we find that endogenous *BMP5* and *BMP7* are induced, without inducing BMP4 or naive state. Similar to induction with BMP4, the TS condition induces transient initial *CDX2* expression, and *CDX2* is also transiently upregulated in an enhanced primed hPSC to TSC conversion without WNT stimulation and inhibiting TGFβ.

Our data suggest that while primed hiPSCs can differentiate via multiple paths, the paths share many established trophoblast specification factors and the final TSCs are similar. Single cell sequencing allows deconvolution of the cellular specification by complete transcriptional profiles. The specification generates two separate cell clusters both with trophoblast expression, and the TSCs preferentially expand to purity with passaging. Further supporting similarity to primary TSC derived from human villous cytotrophoblast and from the blastocyst outgrowth^3^, TSCs derived from primed hPSCs also begin as mixed populations of cells which are passaged to generate pure TSC lines. Our trajectory analysis indicates that *CDX2* and *TFAP2C/A* are important in the initial exit from pluripotency in primed PSCs and facilitates trophoblast lineage specification, similar to human trophoblast specification in vivo*. TFAP2A* is a transcription factor involved in both trophoblast specification and a trophoblast-like amnion speciation. In humans, the first wave of amnion specification may follow a trophoblast pathway^11^. We use multiple statistical tests examining single genes, gene sets and complete transcriptional profile similarities to demonstrate that the primed hPSC-derived TSCs are most transcriptionally similar to trophoblast and not amnion.

We find that five hERV-derived ORFs annotated as human genes are expressed in primed hPSC-derived TSCs, similar to in vivo^57^. hERV are abundant sequences within the human genome that are derived from ancient retroviral infections in the human ancestral germline. hERV-derived long terminal repeats function as human-specific enhancers during placentation and inflammation. hERV-derived envelope proteins create coding ORFs. A specific hERV-envelope protein named Syncytin-1 (*hERVW-1*) is best characterized for cell–cell fusion during embryo attachment and syncytialization where it is upregulated^58^. Like many of the other 668 hERV-envelope proteins expressed in the early embryo and placenta, Syncytian-1 is widely expressed in the inner cell mass, trophoblast and CTBs^57^. Other hERV-derived ORFs inhibit cell fusion (*Syncytin-2*), provide immunosuppressive activities (*Syncytin-2*, *ERV3-1* and *Suppressyn*)^59^, and restrict retroviral infection in the morula, inner cell mass, trophoblast and ESCs (*Suppressyn*)^57^.

The differentiation of primed hiPSCs to trophoblast allows for the modeling of placental diseases from patient-specific iPSCs without first trimester primary tissue and without transitioning through a naive state. While the final TSC product of these different models appears similar, further investigations are needed to examine the differences on TSC models because the naive verses primed states differ in molecular features important for placenta. In the naive state, global DNA demethylation and genomic instability occur in both t2i/L/Go or 5i/L/A conditions^60,66^, and imprinted loci may be aberrantly demethylated^40^. Mammalian imprinting is most pronounced in the placenta^67^ and cellular conditions that maintain imprinting may be important in a cellular model of the human placenta. X chromosome inactivation (XCI) is reset in naive conditions compared to the preservation of inactivated or eroded in primed conditions X chromosomes.

Placental adaptive responses to insults can alter fetal development and predispose the fetus for later diseases, including diabetes and neurodevelopmental disorders^68,69^. As both a protective barrier and a component of the innate immune system, cytotrophoblasts and their progeny play important roles in the outcome of insults^70^. Recent evidence that Covid19 leads to a hyperimmune state has implications for placental biology and fetal development, potentially analogous to the effects of other hyperimmune states during pregnancy^2^. Genetic variation among individuals influences how a pathogen affects the fetus, and iPSC models of this variation could be used to assay potential therapeutic interventions. We anticipate that primer hPSC-derived TSCs will be a powerful model to study the placental contribution to disease and the genetic regulation of human placental evolution.

## Material and methods

### PSC culture

Human ESCs and iPSCs were maintained on Cultrex (1 mg/12 ml DMEM/F12) coated plates in StemFlex media. Media was changed every 48 h in accordance with manufacturer recommendations. Cells were passaged in small clusters using Versene solution. See supplemental methods for details.

### Differentiations and cell maintenance

24 h post passaging with Versene and plating in StemFlex media, adherent cells were washed with DPBS and switched to differentiation media. TS medium: DMEM/F12 with Glutamax supplemented with 0.1 mM 2-mercaptoethanol, 0.2% FBS, 0.3% BSA, 1% ITS-X supplement, 1.5 μg/ml l-ascorbic acid, 50 ng/ml EGF, 2 μM CHIR99021, 0.5 μM A83-01, 1 μM SB431542, 0.8 mM VPA and 5 μM Y27632. BA condition: 10 ng/ml BMP4, DMEM/F12 with Glutamax supplemented with l-ascorbic acid 2-phosphate magnesium. BI condition: 10 ng/ml BMP4 and 2 μM IWP2, in DMEM/F12 with Glutamax supplemented with l-ascorbic acid 2-phosphate magnesium. Cells were collected on day 2, 4, and 6 for RNA sequencing. For the TS condition, after cells were cultured in TS media for 6 days, they were passaged using TrypLE express at a split ratio of 1:3 and plated on new Collagen IV (5 μg/mL) coated plates. After the first passage, media was changed every 48 h and split at a ratio of 1:3–1:6 every 3–4 days. See supplemental methods for more details.

### EVT and STB differentiation and characterization

Differentiation of TSCs were performed as previously described^3^, with minor modifications. For EVT differentiation, 24-well plates ibidi were coated with 1 μg/mL Collagen IV overnight. 2 × 10^5^ TSC were seeded per well in 500 µL EVT basal medium [DMEM/F12 supplemented with 0.1 mM β-mercaptoethanol, 0.5% penicillin–streptomycin, 0.3% BSA, 1% ITS-X, 7.5 μM A83-01, 2.5 μM Y27632] supplemented with 4% KSR and 100 ng/mL NRG1 alpha. Matrigel was added to a 2% final concentration shortly after resuspending TSC in the medium. On day 3, the media was replaced with 500 µL EVT basal medium supplemented with 4% KSR, and Matrigel was added to a 0.5% final concentration. At day 6, the media was replaced with 500 µL EVT basal medium, and Matrigel was added to a 0.5% final concentration. At day 9, the cells were ready for analysis.

For STB differentiation, 24-well plates ibidi were coated with 2.5 μg/mL Collagen IV overnight. 2 × 10^5^ TSCs were seeded per well in 500 µl STB medium [DMEM/F12 supplemented with 0.1 mM β-mercaptoethanol, 0.5% penicillin–streptomycin, 0.3% BSA, 1% ITS-X, 2.5 μM Y-27632, 2 μM Forskolin and 4% KSR]. The media was changed at day 3, and at day 4 the cells were ready for analysis.

### Supplementary Information

Supplementary Information 1.

Supplementary Information 2.

Supplementary Information 3.

Supplementary Table S1.

Supplementary Table S2.

Supplementary Table S3.

Supplementary Table S4.

Supplementary Table S5.

Supplementary Table S6.

Supplementary Table S7.

Supplementary Table S8.

Supplementary Table S9.

Supplementary Table S10.

Supplementary Table S11.

Supplementary Table S12.

Supplementary Table S13.

## Data Availability

The Single cell RNA sequencing datasets generated and/or analyzed during the current study have been deposited in ArrayExpress with the accession code E-MTAB-9526. Analysis pipelines are publicly deposited on Github:https://github.com/paquolalab/placenta_ips.
