# Supplementary material for: Human archetypal pluripotent stem cells differentiate into trophoblast stem cells via endogenous BMP5/7 induction without transitioning through naive state
Source: Sci Rep. 2024 Feb 8;14:3291. doi: 10.1038/s41598-024-53381-w (PMC10853519; doi:10.1038/s41598-024-53381-w)
Supplement: Supplementary file 1 — Supplementary Information 1. [file 41598_2024_53381_MOESM1_ESM.docx]

**Supplementary information**

**A single cell trajectory of human archetypal pluripotent stem cell differentiation to trophoblast stem cells reveals induction of endogenous BMP5/7 and GATA3 without transitioning through a naive state**

Ethan Tietze^a,#^, Andre Rocha Barbosa^a,b,c,#^, Bruno Araujo^a,#^, Veronica Euclydes^a,b,d,#^, Bailey Spiegelberg^a,e^, Hyeon Jin Cho^a^, Yong Kyu Lee^a^, Yanhong Wang^a^, Alejandra McCord^a^, Alan Lorenzetti^a^, Arthur Feltrin^a,f^, Joyce van de Leemput^a,$^, Pasquale Di Carlo^a,g^, Gianluca Ursini^a,h^, Kynon J. Benjamin^a,b,i^, Helena Brentani^c,d^, Joel E. Kleinman^a,i^, Thomas M. Hyde^a,b,i^, Daniel R. Weinberger^a,b,e,h,i^, Ronald McKay^a,h^, Joo Heon Shin^a,b^, Tomoyo Sawada^a,b,*^, Apua C.M. Paquola^a,b^, and Jennifer A. Erwin^a,b,h,*^

1. Lieber Institute for Brain Development, Baltimore, Maryland, USA
2. Department of Neurology, Johns Hopkins School of Medicine, Baltimore, Maryland, USA
3. Inter-institutional Graduate Program on Bioinformatics, University of São Paulo, São Paulo, SP, Brazil
4. Department of Psychiatry, University of Sao Paulo, Medical School, São Paulo, Brazil
5. Department of Genetic Medicine, Johns Hopkins University School of Medicine, Baltimore, Maryland, USA
6. Center for Mathematics, Computation and Cognition, Federal University of ABC, Santo André, SP, Brazil
7. Department of Basic Medical Science, Neuroscience, and Sense Organs – University of Bari Aldo Moro, Bari, Italy
8. Department of Neuroscience, Johns Hopkins School of Medicine, Baltimore, Maryland, USA
9. Department of Psychiatry & Behavioral Sciences, Johns Hopkins University School of Medicine, Baltimore, MD, USA

$ Present address: Center for Precision Disease Modeling and Division of Endocrinology, Diabetes and Nutrition, Department of Medicine, University of Maryland School of Medicine, Baltimore, Maryland, 21201, USA

#These authors contributed equally to this study.

*Lead contact and corresponding author: Tomoyo.Sawada@libd.org; Jennifer.Erwin@libd.org

**This PDF file includes:**

Supplementary Figures 1 to 10

Legend for supplementary tables 1 to 13

Key Reagents and Resources

Supplemental Methods

References

**Supplementary Figures**

**
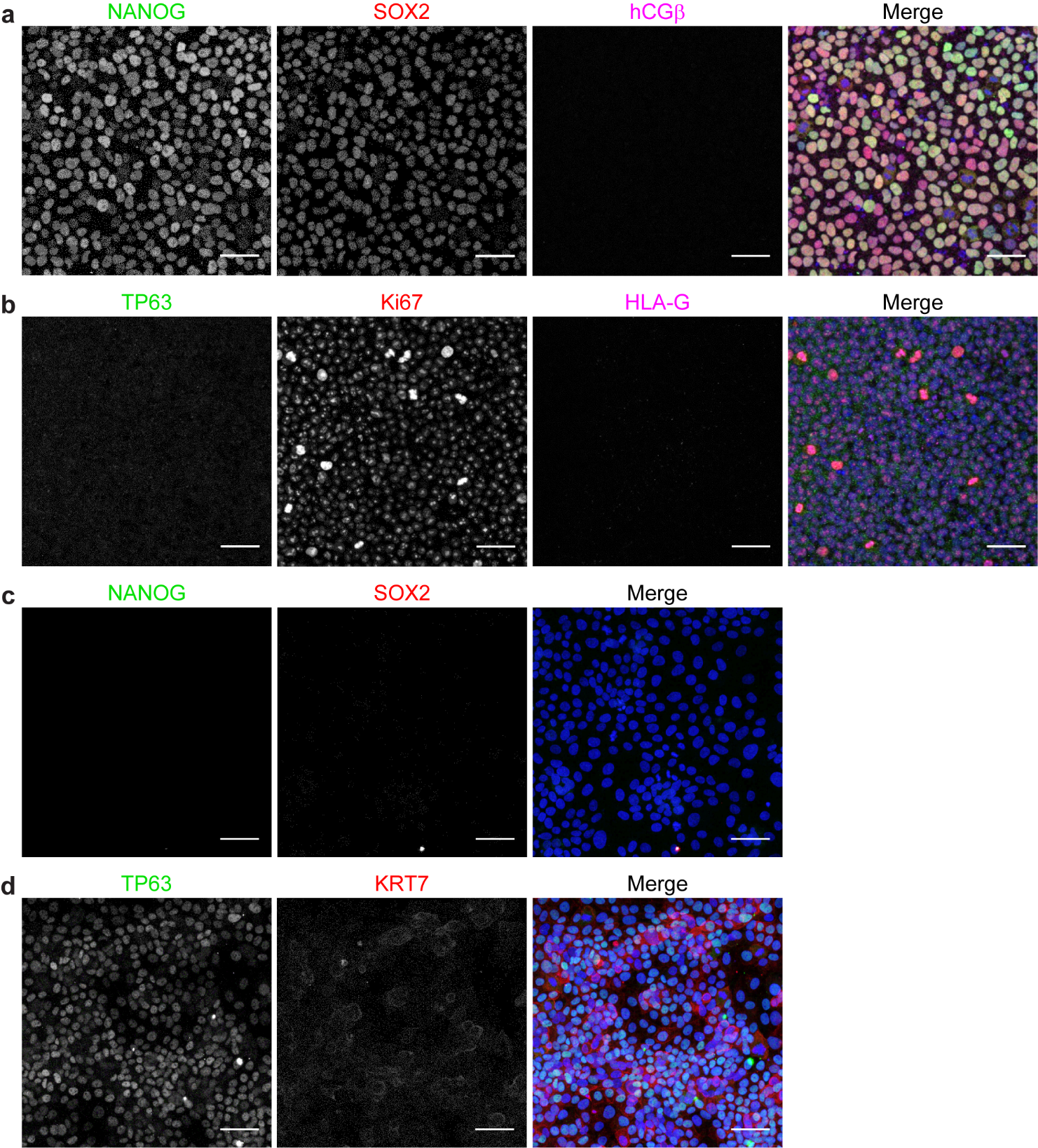
**

**Supplementary Fig. 1: The loss of pluripotent stem cell (PSC) marker expression and the acquisition of trophoblast-specific marker expression occur during the differentiation of hPSCs into trophoblast stem cells (TSCs).** (**a**) Immunofluorescent images of hiPSC LIBD7c6 for PSC markers NANOG and SOX2, and a trophoblast marker hCGβ. (**b**) Immunofluorescent images of hiPSC LIBD7c6 for a proliferation marker Ki67 and trophoblast markers TP63 and HLA-G. (**c**) Immunofluorescent images of TSCs derived from hiPSC 2014.06 for PSC markers NANOG and SOX2. (**e**) Immunofluorescent images of TSCs derived from hiPSC 2014.06 for trophoblast markers TP63 and KRT7. Nuclei were stained with Hoechst 33342 (blue). Scare bars, 50 μm.

**
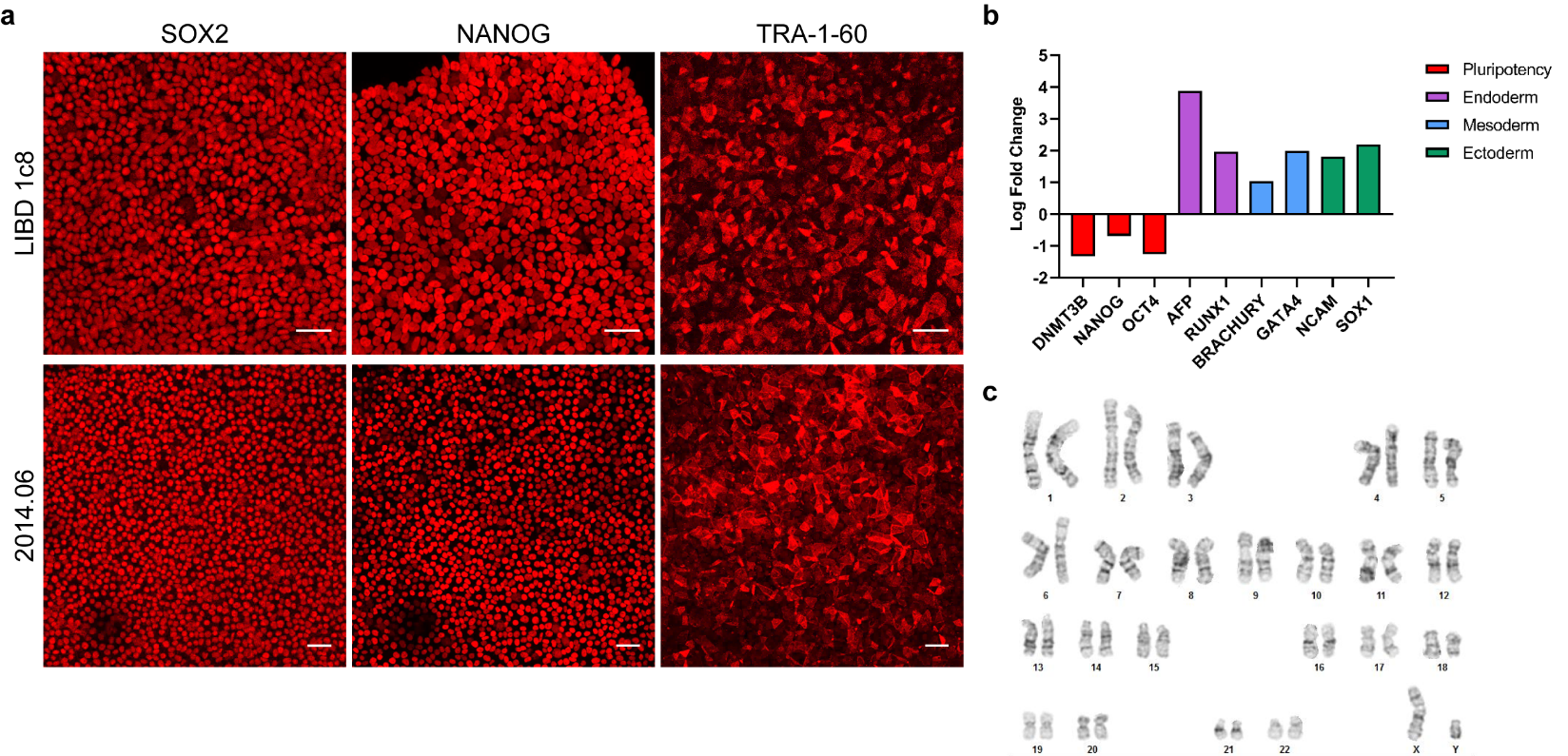
**

**Supplementary Fig. 2: Characterization of hiPSC lines.** (**a**) Immunofluorescent images of hiPSC lines LIBD1c8 and 2014.06 for PSC markers SOX2, NANOG and TRA-1-60. (**b**) Relative expression levels of marker genes for PSC and three germ layers in spontaneously differentiated hiPSC 2014.06, normalized to their respective expression levels in undifferentiated condition by RT-qPCR. (**c**) G-band karyotyping of hiPSC 2014.06. Scare bars, 50 μm.


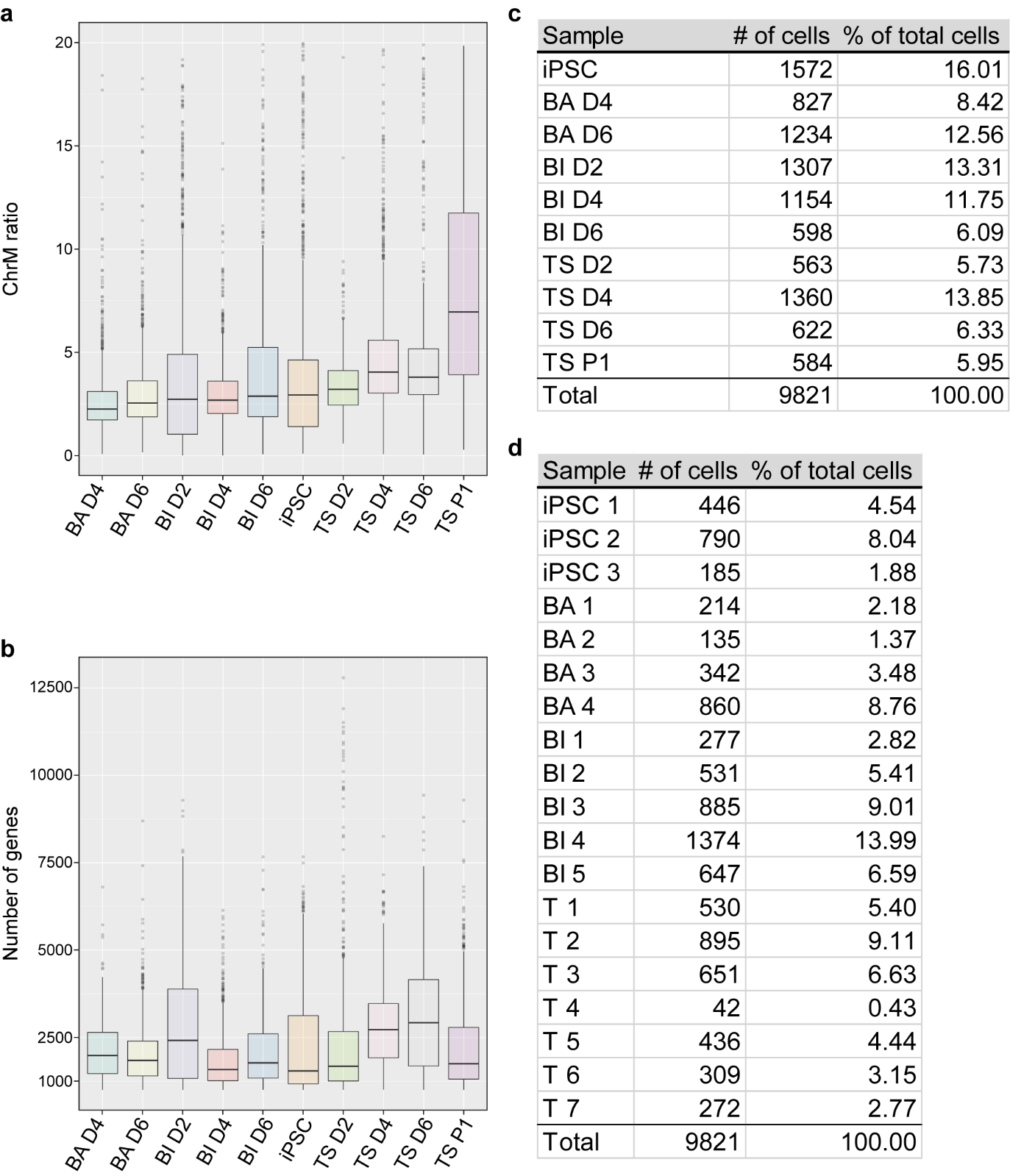


**Supplementary Fig. 3: Quality control metrics for scRNA-seq/Drop-seq analysis.** (**a**) Box plot showing the mapping rate for mitochondrial genes per droplet. (**b**) Box plot showing the number of genes detected per droplet. (**c**) Summary table presenting the number of cells in each experimental sample. The composition of cells from each experimental sample across the data set is also shown. (**d**) Summary table showing the number of cells within each cell cluster. The percentage composition of cells from each cell cluster across the dataset is also indicated.


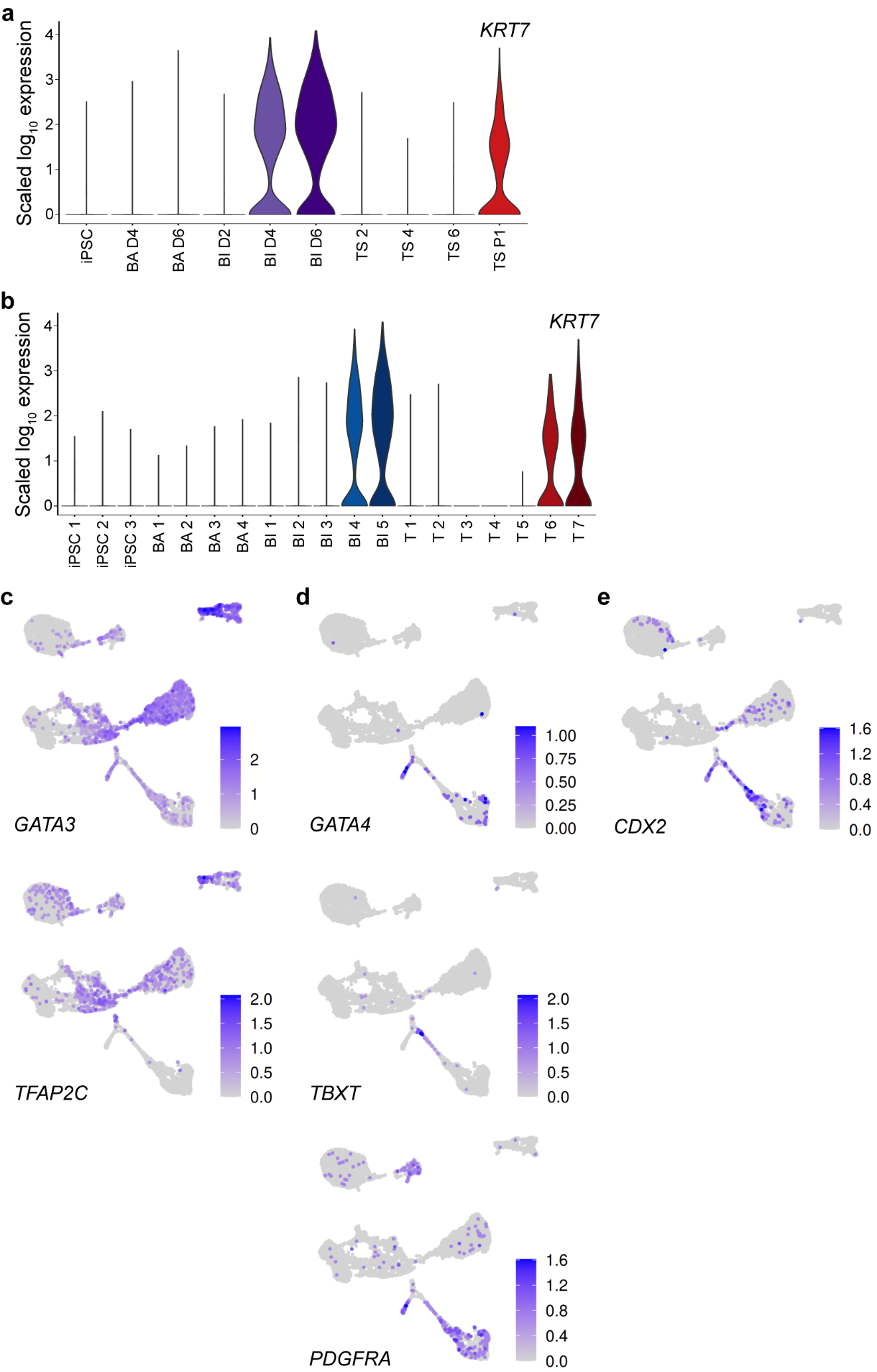


**Supplementary Fig. 4: Representative gene expressions in the cells differentiated from hiPSCs in different conditions.** (**a, b**) Violin plots showing the expression of KRT7 in each experimental samples (**a**) and each cell cluster (**b**). (**c**, **d**) UMAP showing the expression of marker genes for trophoblast (*GATA3*, *TFAP2C* and *CDX2*, **c**) and mesodermal-lineage cells (*GATA4*, *TBXT* and *PDGFRA*, **d**).


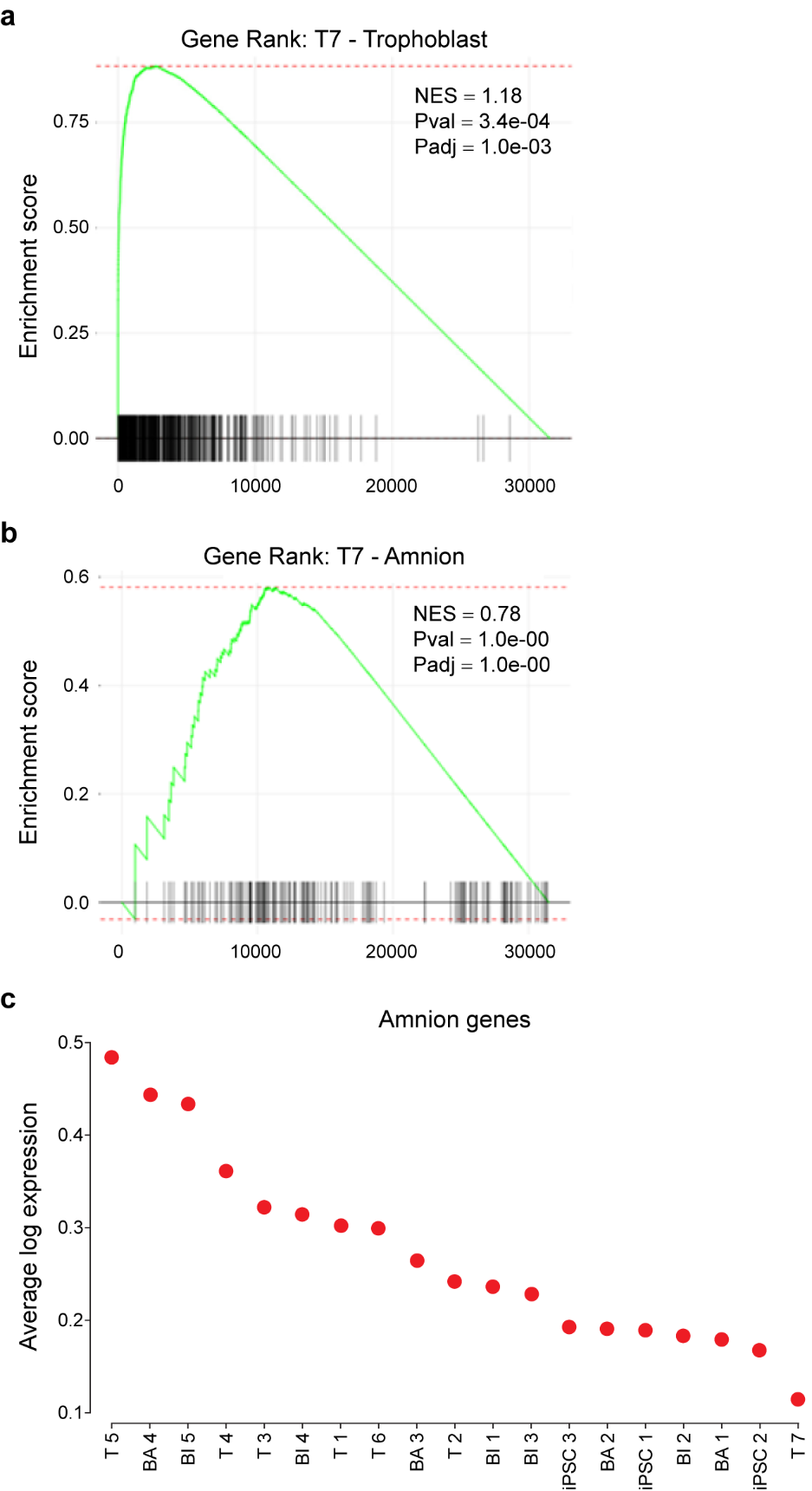


**Supplementary Fig. 5: Gene set enrichment analysis (GSEA) in the T7 cells for trophoblast and amnion genes.** (**a**, **b**) Gene rank for trophoblast (**a**) and amnion (**b**) in cells in the T7 cluster. NES, normalized enrichment score. (**c**) Average expression of amnion genes across the cell clusters.

**
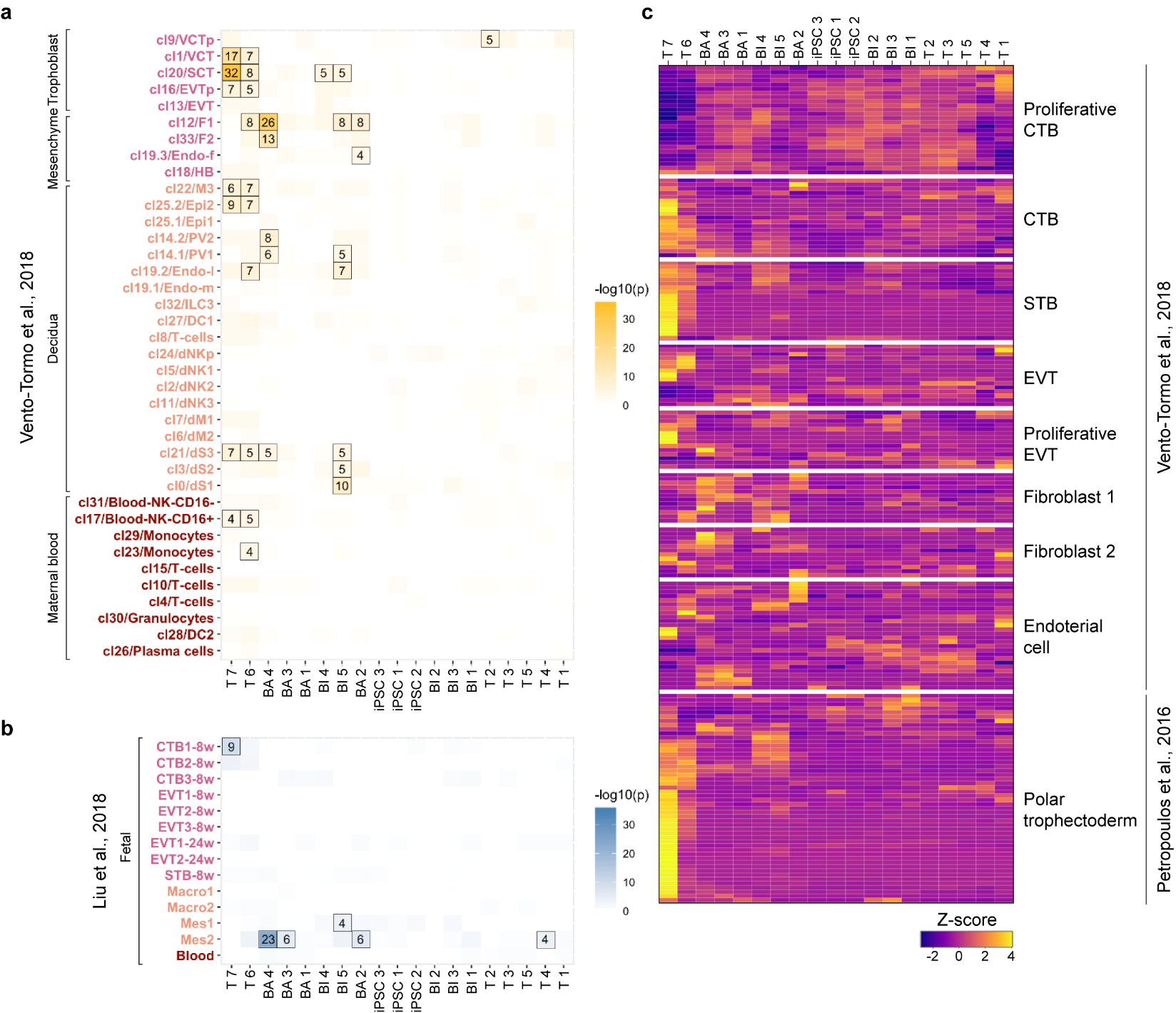
**

**Supplementary Fig. 6: Gene expression patterns in hiPSC-derived TSCs are enriched for human placental cells during the first trimester.** (**a**, **b**) GESA results demonstrating the enrichment of cell clusters to cell types identified in first trimester placentas [25, 26]. Color and number in each square indicate the −log_10_(p-value) obtained through Bonferroni-corrected hypergeometric tests for the enrichment of cell-specific genes in hiPSC-derived cell clusters concerning the cell types identified in first-trimester placentas [25, 26]. (**c**) Heatmap of the normalized expression of the top unique marker genes of placental cell types of fetal origin [26] and of polar trophectoderm [44].


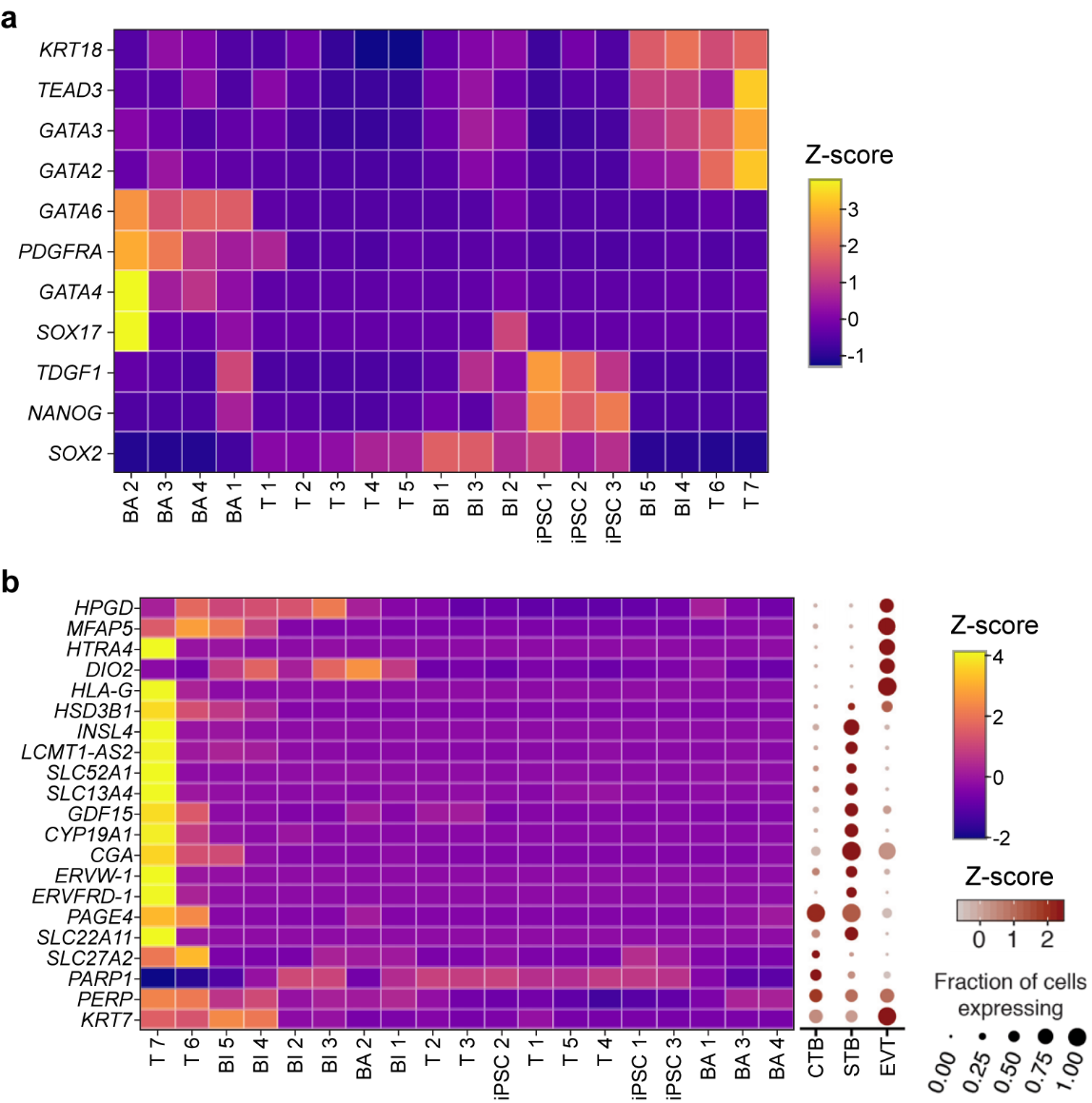


**Supplementary Fig. 7: Lineage specification and cell cluster identity based on marker gene expression profiles.** (**a**) Heatmap showing the expression of high confidence marker genes for epiblast, primitive endoderm, and trophectoderm in each cell cluster [supplementary ref. 1]. (**b**) Heatmap showing the expression of marker genes for trophoblast-lineage cells (left). Dot plot displaying the expression of these genes in primary villous cytotrophoblast (CTB), syncytiotrophoblast (STB), and extravillous trophoblast (EVT), modified dot plot from [supplementary ref. 2].


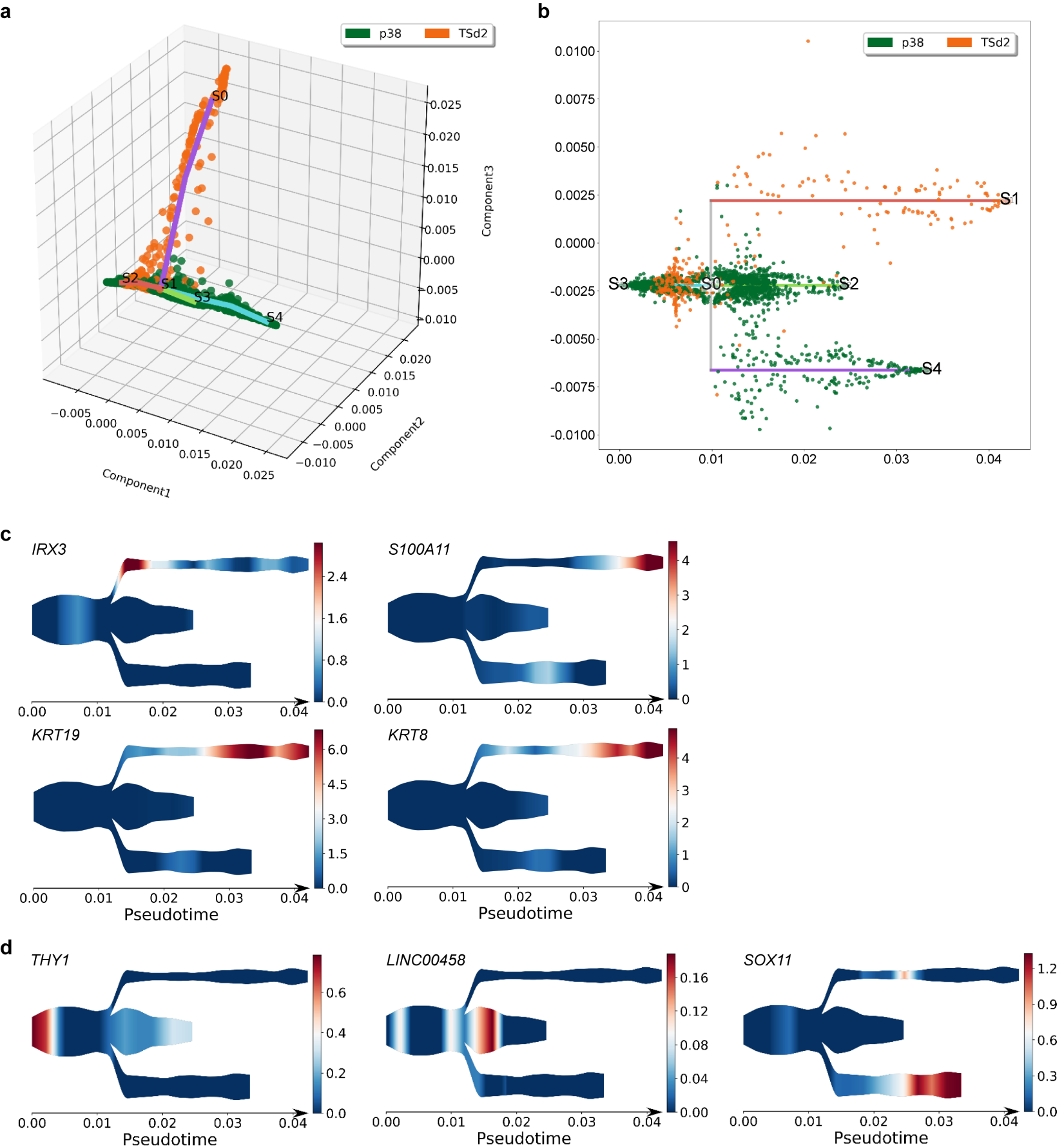


**Supplementary Fig. 8: Developmental trajectory of TSC derivation from hiPSC under TS condition.** (**a**, **b**) Visualization of branches assigned using the first principal components (**a**) and the resulting subway map representation of the reconstructed pseudotime trajectory using hiPSCs and cells in the TS D2 condition (**b**). (**c**) Expression of genes showing the significant correlation with the pseudotime transition from S0 to S1 (TSC-lineage). (**d**) Expression of primed PSC marker genes expressed along psuedotime.

**
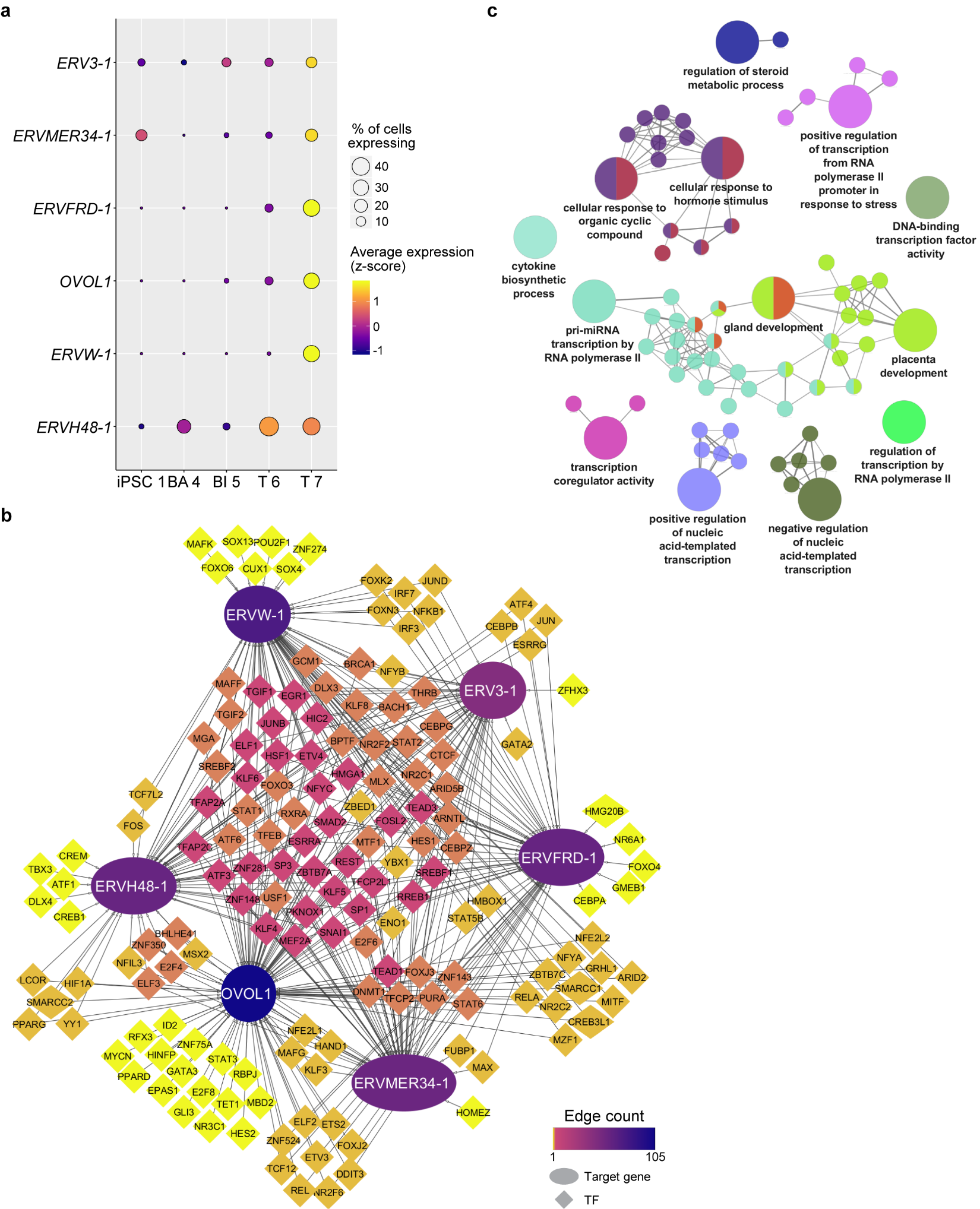
**

**­Supplementary Fig. 9: Endogenous retrovirus-derived genes participate in a gene regulatory subnetwork uniquely in cells derived from the TS condition.** (**a**) Dot plot showing the expression of *HERV* genes in the cells at the most mature state in each differentiation condition (T7, T6, BI5 and BA4) and in hiPSC cluster (iPSC1). (**b**) Cytoscape representation of the top transcription factor (TF) partners of *ERVs* detected in cells in the TS7 cluster (164 TFs and 6 targets genes are indicated). (**c**) Visual representation of gene ontology terms enriched in the most significant ERVs partners show in **b** (*p*<0.0001).


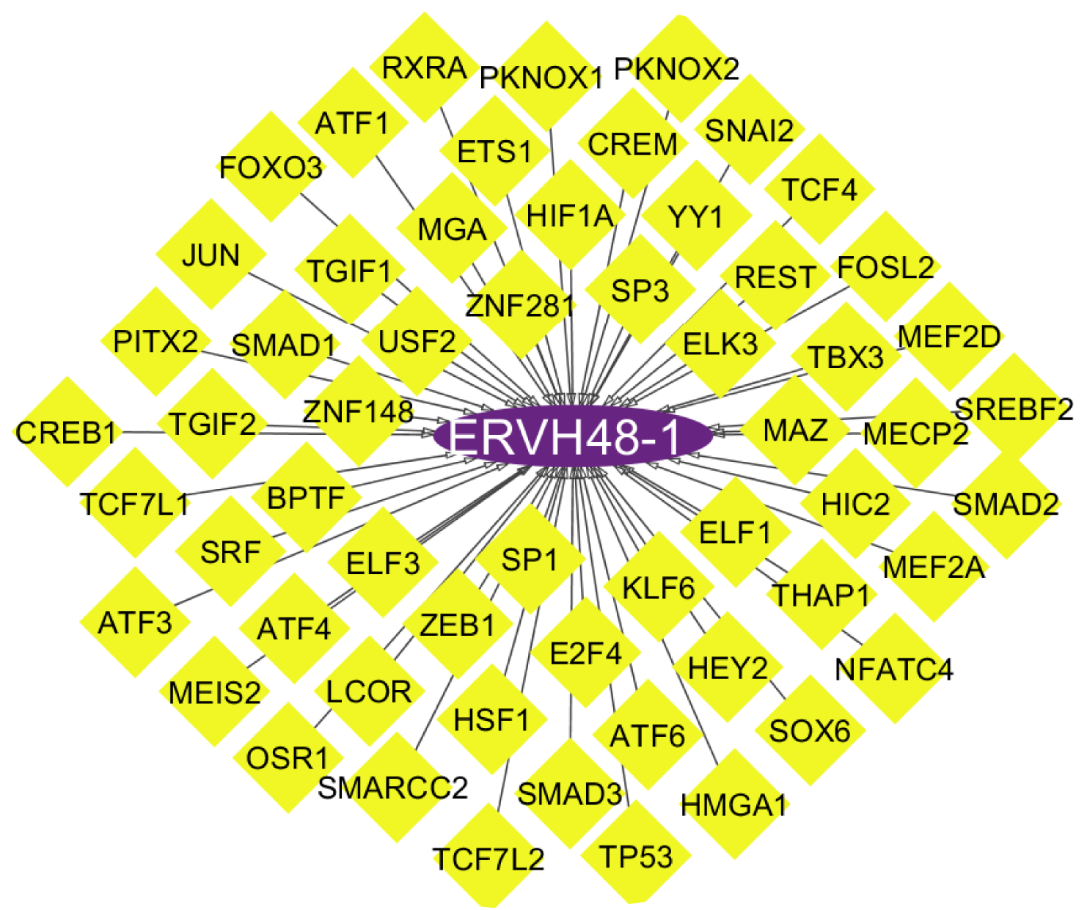


**Supplementary Fig. 10: PANDA network analysis reveals unique regulation of endogenous retrovirus-derived genes in cells differentiated under the BA condition.** Cytoscape representation of *ERV48-1* connected network identified by PANDA in cells in the BA4 cluster.

**Legend for supplementary tables**

**Table S1** (separate file): Number of cells from each differentiation condition/day that are assigned to the cluster.

**Table S2** (separate file): Differentially expressed genes in each cluster compared to all other clusters.

**Table S3** (separate file): Differentially expressed genes analyzed by pairwise comparisons of one cluster compared to another cluster.

**Table S4** (separate file): Average log normalized expression of amnion genes for each cluster shown in Fig. 2b.

**Table S5** (separate file): Average log normalized expression of amnion genes for each cluster shown in Fig. 2c.

**Table S6** (separate file): Pairwise comparisons of amnion gene expression among cell clusters shown in Fig. 2c.

**Table S7** (separate file): Percentage of cells expressing BMP signaling genes in each cell cluster (Fig. 2c) and cells expressing marker genes for naïve and primed human pluripotent stem cells in each differentiation condition (Fig. 2b).

**Table S8** (separate file): STRAM analysis of transition and differentially expressed leaf genes in iPSC compared to TS D2.

**Table S9** (separate file): Differentially expressed genes in the node S1 compared to the node S0 in STREAM analysis.

**Table S10** (separate file): Differentially expressed genes in the node S2 compared to the node S0 in STREAM analysis.

**Table S11** (separate file): Differentially expressed genes in the node S4 compared to the node S0 in STREAM analysis.

**Table S12** (separate file): Differentially expressed genes in the node S3 compared to the node S0 in STREAM analysis.

**Table S13** (separate file): Gene ontology enrichment analysis of the ERV-associated gene regulatory network.

**Key Reagents and Resources**

| Reagent or Resource Type | Name | Supplier | Product Number | Additional information |
| --- | --- | --- | --- | --- |
| Antibody | anti-hCGβ (mouse monoclonal) | Abcam | ab9582 | 1:100 |
| Antibody | anti-HLAG (mouse monoclonal) | Abcam | ab52455 | 1:100 |
| Antibody | anti-KI67 (rabbit polyclonal) | Abcam | ab15580 | 1:250 |
| Antibody | anti-KRT7 (mouse monoclonal) | ThermoFisher Scientific | MA5-11986 | 1:1000 |
| Antibody | anti-TJP1 (mouse monoclonal) | ThermoFisher Scientific | 33-9100 | 1:100 |
| Antibody | anti-TP63 (goat polyclonal) | Novus Biologicals | AF1916 | 1:100 |
| Antibody | anti-VIM (rabbit monoclonal) | Abcam | ab92547 | 1:500 |
| Antibody | anti-SDCA1 (rabbit monoclonal) | Abcam | Ab128936 | 1:500 |
| Antibody | anti-CD49f (Integrin alpha 6 – rat monoclonal - FITC) | eBioscience | 11-0495-82 | 0.25µg |
| Antibody | Isotype control anti-IgG2a kappa (rat – FITC) | eBioscience | 11-4321-42 | 0.25µg |
| Antibody | Donkey anti-mouse IgG AF-488 | Jackson Immunoresearch Laboratories | 715-545-151 | 1:500 |
| Antibody | Donkey anti-mouse IgG Cy3 | Jackson Immunoresearch Laboratories | 715-165-151 | 1:500 |
| Antibody | Donkey anti-rabbit IgG AF-488 | Jackson Immunoresearch Laboratories | 711-545-152 | 1:500 |
| Antibody | Donkey anti-rabbit IgG Cy3 | Jackson Immunoresearch Laboratories | 711-005-152 | 1:500 |
| Antibody | Donkey anti-rabbit IgG AF-647 | Jackson Immunoresearch Laboratories | 711-605-152 | 1:500 |
| Antibody | Donkey anti-goat IgG Cy3 | Jackson Immunoresearch Laboratories | 705-165-147 | 1:500 |
| Chemical, Peptide, or Recombinant Protein | 2-mercaptoethanol | ThermoFisher Scientific | 21985023 |  |
| Chemical, Peptide, or Recombinant Protein | Bmp4 | Peprotech | 120-05-5ug |  |
| Chemical, Peptide, or Recombinant Protein | Bovine Serum Albumin | Cell Signaling Technology | 9998S |  |
| Chemical, Peptide, or Recombinant Protein | Cell Banker 1 Cryopreservation Media | Amsbio | 11888 |  |
| Chemical, Peptide, or Recombinant Protein | Collagen IV | Corning | 354233 |  |
| Chemical, Peptide, or Recombinant Protein | Cultrex Reduced Growth Factor Basement Membrane Extract, PathClear | R&D Systems | 3433-005-01 |  |
| Chemical, Peptide, or Recombinant Protein | DMEM/F12, Glutamax supplement | ThermoFisher Scientific | 10565018 |  |
| Chemical, Peptide, or Recombinant Protein | DPBS, no calcium, no magnesium | ThermoFisher Scientific | 14190250 |  |
| Chemical, Peptide, or Recombinant Protein | Epidermal Growth Factor | Peprotech | AF-100-15 |  |
| Chemical, Peptide, or Recombinant Protein | Fetal Bovine Serum | ThermoFisher Scientific | 16140071 |  |
| Chemical, Peptide, or Recombinant Protein | Gelatin | Sigma-Aldrich | G1393-100mL |  |
| Chemical, Peptide, or Recombinant Protein | Hoechst 33342 | ThermoFisher Scientific | H3570 |  |
| Chemical, Peptide, or Recombinant Protein | Insulin-transferrin-selenium | Corning | 25-800-CR |  |
| Chemical, Peptide, or Recombinant Protein | ITS-X | ThermoFisher Scientific | 51500056 |  |
| Chemical, Peptide, or Recombinant Protein | IWP2 | BioGems | 6866167-1mg |  |
| Chemical, Peptide, or Recombinant Protein | L-Ascorbic Acid | BioGems | 2520691 |  |
| Chemical, Peptide, or Recombinant Protein | L-ascorbic acid 2-phosphate magnesium | Sigma-Aldrich | A8960-5G |  |
| Chemical, Peptide, or Recombinant Protein | SB431542 | BioGems | 3014193 |  |
| Chemical, Peptide, or Recombinant Protein | StemFlex Media | ThermoFisher Scientific | A3349401 |  |
| Chemical, Peptide, or Recombinant Protein | TrypLE express | ThermoFisher Scientific | 12604013 |  |
| Chemical, Peptide, or Recombinant Protein | Valproic Acid Sodium salt | BioGems | 1066656 |  |
| Chemical, Peptide, or Recombinant Protein | Y-27632 | BioGems | 1293823 |  |
| Chemical, Peptide, or Recombinant Protein | Forskolin | BioGems | 6652995 |  |
| Chemical, Peptide, or Recombinant Protein | Recombinant human NRG1 alpha | BioLegend | 765704 |  |
| Cell Line | 2014.06 Human Induced Pluripotent Stem Cells | Lieber Institute | N/A |  |
| Cell Line | CF-1 Mouse Embryonic Fibroblasts | ThermoFisher Scientific | A34181 |  |
| Cell Line | LIBD1c8 Human Induced Pluripotent Stem Cells | Sawada et al., 2020 [21] | N/A | Generated in lab |
| Cell Line | LIBD7c6 Human Induced Pluripotent Stem Cells | Sawada et al., 2020 [21] | N/A | Generated in lab |
| Cell Line | LIBD9c1 Human Induced Pluripotent Stem Cells | Sawada et al., 2020 [21] | N/A | Generated in lab |
| Cell Line | WA-01 (H1) Human Embryonic Stem Cells | Wicell | WB0197 |  |
| Cell Line | L0001A_X04 Human Induced Pluripotent Stem Cells | Lieber Institute | N/A | Generated in lab |
| Cell Line | L0002A_X03 Human Induced Pluripotent Stem Cells | Lieber Institute | N/A | Generated in lab |
| Cell Line | L0003B_X01 Human Induced Pluripotent Stem Cells | Lieber Institute | N/A | Generated in lab |
| Cell Line | CT27 Primary Trophoblast Stem Cells from Placenta | RIKEN BRC (Okae et al., 2018 [3]) | RCB4936 |  |
| Cell Line | CT29 Primary Trophoblast Stem Cells from Placenta | RIKEN BRC (Okae et al., 2018 [3]) | RCB4937 |  |
| Cell Line | bTS11 Primary Trophoblast Stem Cells from Blastocyst | RIKEN BRC (Okae et al., 2018 [3]) | RCB4941 |  |
| Cell Line | BTS5 Primary Trophoblast Stem Cell from Blastocyst | RIKEN BRC (Okae et al., 2018 [3]) | RCB4940 |  |

**Supplemental Methods**

**hPSC culture:** 6-well plates were coated with reduced growth factor Cultrex (1mg/12 ml DMEM/F12) at 37°C for at least one hour. Human ESC and iPSC were maintained on coated plates in Stemflex media. Media was changed every 48 hrs in accordance with manufacturer recommendations. Cells were passaged in small clusters using Versene solution and a split ratio of 1:10-1:12. For cryopreservation, cells were suspended in PSC cryopreservation medium and temporarily stored in a deep freezer at −80°C before being transferred to liquid nitrogen for long term storage.

**Differentiations and Cell Maintenance:** 6-well plates were coated with reduced growth factor Cultrex (1mg/12 ml DMEM/F12) at 37°C for at least one hour. 80% confluent PSC were harvested using Versene solution such that small cellular aggregates each containing approximately 5-20 cells were passaged without centrifugation. These cellular aggregates were seeded at a ratio of 1:12 in a new well, and cultured in 2 mL of StemFlex medium. After 24 hours, cells were washed with DPBS and 2 mL of the indicated media was added to each differentiating well. TSC medium: DMEM/F12 with Glutamax supplemented with 0.1 mM 2-mercaptoethanol, 0.2% FBS, 0.3% BSA, 1% ITS-X supplement, 1.5 μg/ml L-ascorbic acid, 50 ng/ml EGF, 2 μM CHIR99021, 0.5 μM A83-01, 1 μM SB431542, 0.8 mM VPA and 5 μM Y27632. BMP4 Alone (BA condition): 10 ng/ml BMP4, DMEM/F12 with Glutamax supplemented with L-ascorbic acid 2-phosphate magnesium. BMP4 + IWP2 (BI condition): 10 ng/ml BMP4 and 2uM IWP2, in DMEM/F12 with Glutamax supplemented with L-ascorbic acid 2-phosphate magnesium. Every 24 hours, 2 mL of fresh medium was added to each well. Cells were collected on day 2, 4, and 6 for single cell RNA sequencing. For the TS condition, cells were passaged using TrypLE express 6 days after switching from StemFlex to TS media at a split ratio of 1:3 and plated on new Col IV (5 ug/mL) coated plates. After the first passage, cells were fed every 48 hours and split at a ratio of 1:3-1:6 every 3-4 days when 80% confluent. TSC cultures between passages 10-20 were used for all experiments unless otherwise noted. Cells were cultured in humidified incubators 5% CO2 and 20% O2. For cryopreservation, TSC were suspended in Cell Banker 1 and temporarily stored in a deep freezer at −80°C before being transferred to liquid nitrogen for long term storage.

**EVT and STB differentiation:** Differentiation of TSCs were performed as previously described [supplementary ref. 3], with minor modifications. For EVT differentiation, 24-well plates ibidi were coated with 1 μg/mL Collagen IV overnight. 2 × 105 TSC were seeded per well in 500µL EVT basal medium [DMEM/F12 supplemented with 0.1 mM β-mercaptoethanol, 0.5% penicillin-streptomycin, 0.3% BSA, 1% ITS-X, 7.5 μM A83-01, 2.5 μM Y27632] supplemented with 4% KSR and 100 ng/mL NRG1 alpha. Matrigel was added to a 2% final concentration shortly after resuspending TSC in the medium. On day 3, the media were replaced with 500µL EVT basal medium supplemented with 4% KSR, and Matrigel was added to a 0.5% final concentration. At day 6, the media were replaced with 500µL EVT basal medium, and Matrigel was added to a 0.5% final concentration. At day 9, the cells were ready for analysis. For STB differentiation, 24-well plates ibidi were coated with 2.5 μg/mL Collagen IV overnight. 2 × 105 TSCs were seeded per well in 500µl STB medium [DMEM/F12 supplemented with 0.1 mM β-mercaptoethanol, 0.5% penicillin-streptomycin, 0.3% BSA, 1% ITS-X, 2.5 μM Y-27632, 2 μM Forskolin and 4% KSR]. The media was changed at day 3, and at day 4 the cells were ready for analysis.

**Flow Cytometry:**

Trophoblast stem cells-like were dissociated with TrypLE, passed through a 70 μM mesh filter, and suspended in 2% BSA/PBS. For flow cytometric analysis of ITGA6, cells were incubated with FITC-conjugated anti-CD49f (Integrin alpha 6 - ITGA6) for 30 minutes at room temperature and then fixed with 4% PFA for 15min. FITC-conjugated rat IgG2a was used as isotype control. Flow cytometry was carried out using the Becton Dickinson LSRII and the acquisition was done in BD FACSDiva Software Version 8.0.1. The data were analyzed using FlowJo software.

**Immunofluorescence Staining:**

Cells were washed in Tris Buffered Saline (TBS), fixed with 4% paraformaldehyde for ten minutes, and washed three times in TBS. Cells were then incubated in blocking solution (3% horse serum, 0.1% Triton-X in TBS) for one hour at room temperature before being incubated with primary antibodies in blocking solution overnight at 4°C. Cells were then rinsed twice and incubated with secondary antibodies in blocking solution for two hours at room temperature. After two washes in TBS cells were counterstained with Hoechst 33342 and rinsed twice in TBS. Cells were imaged using a Zeiss LSM700 or LSM780 confocal microscope. The brightness and/or contrast was globally adjusted for some images.

**HLA-G and Fusion Index Analysis:**

The HLA-G positive cells were calculated using the following formula: [(EVT-N)/T x 100]). EVT, the number of HLA-G positive cells; N, the number of HLA-G negative cells; T, total number of nuclei counted. The fusion index represents the percentage of cell-cell fusion events. Syncytia were defined as cells with at least three nuclei. The number of Hoechst 33342I-stained nuclei and syncytia were counted using Image-J. The fusion index was calculated using the following formula: [(N-S)/T] x 100. N, the number of nuclei in the syncytia; S, the number of syncytia; T, total number of nuclei counted.

**Bulk RNA sequencing and analysis:**

Total RNA was extracted from cells using a Direct-zol Miniprep kit (Zymo Research). Libraries were prepared using TruSeq Stranded Total RNA Ribo-Zero H/M/R Gold and sequenced on NovaSeq 6000 (illumina). Primed hPSC lines: H1, L0001A_X04, L0002A_X03, L0003B_X01, LIBD1c8, and LIBD9c1, TSCs derived from these hPSC lines and primary TSCs: CT27, CT29, bTS11, bTS5 [3] were analyzed. We manually annotated sample cell types by combining available metadata and information from original publications. We processed the data by mapping the reads to the human reference transcriptome (Ensembl version 106) using Salmon, and importing resulting counts into the R programming language environment by summarizing gene-level counts using tximport. We removed genes with low expression using the default parameters of edgeR’s *filterByExpr* function. Raw counts were controlled for study-associated batch effects using ComBat-seq and normalized using DESeq2’s *varianceStabilizingTransformation* function. The resulting dataset was input into DESeq2’s *plotPCA* function to perform a principal component analysis (PCA) using the 500 genes with the greatest variance. We plotted the PCA using ggplot2, featuring points relevant to the validation of the lines developed in this study.

**Single Cell sequencing by Drop-seq:**

*Dissociation of Cells for Drop-seq*

Human iPS cells were grown to 80-90% confluency. After washing cells with DPBS (Thermo Fisher Scientific 14190250) cells were incubated with Versene (Thermo Fisher Scientific 15040066) solution for approximately 10 minutes at 37 °C and gently washed up and down with a P1000 pipette to generate a single cell suspension. Cells were then diluted in Stemflex media and centrifuged at 200g for 4 min before being resuspended in 1 mL of 1X PBS, pH 7.4 (Gibco, # 10010023) with 0.01% BSA (Invitrogen, # AM2616) at the concentration of 100 cells/µL.

*Droplet generation*

Barcoded beads (Chemgenes, # Macosko-2011-10) were washed with 30 mL of ethanol (Sigma Aldrich, # E7023-500ML), then removed all ethanol after centrifugation at 1,000g for 1 minute. Washed beads were suspended in 30 mL of TE-TW (10 mM Tris pH8.0, 1 mM EDTA, 0.5% SDS) and spun down at 1,000g for 1 minute. TE-TW was discarded and re-suspended in 20 mL of TE-TW. 100 µm strainer (Corning, Cat# 352360) was used for size selection of beads.

Beads were suspended as the concentration of 120 beads/µL in 1 mL of the lysis buffer. To generate droplets, cells, beads, and oil (BioRad, #186-4006) were loaded into the 125-μm microfluidic device after aquapel flush treatment (FlowJEM). The cells and barcoded beads suspension were flown at 2 mL/h each and at 13 mL/h for oil.

*Droplet breakage, reverse transcription, and exonuclease I treatment*

Droplets were collected into the 50 mL tubes and 30 mL of 6X SSC was added, then broken with 1 mL of perfluorooctanol (Sigma, # 370533). Collected beads were washed with 1 mL of 6X SSC twice and with 300 µLof RT buffer. On removing the RT buffer, reverse transcription mixture was added into the beads. To synthesize cDNA, beads in RT mix were incubated with rotation at room temperature for 30 minutes and 42 °C for 90 minutes. The reverse transcription mixture was prepared as described by Macosko, E.Z. et. al [supplementary ref. 4]. Beads were washed once with 1 mL of TE-SDS, twice with 1 mL of TE-TW, and with 1 mL of 10 mM Tris pH 8.0. Exonuclease I treatment was performed to remove excess bead primers at 37 °C for 45 minutes with rotation followed by washing beads once with 1 mL of TE-SDS, twice with 1 mL of TE-TW, and with 1 mL of molecular grade water.

*PCR amplification, clean-up with SPRI beads, and library preparation*

Unique barcode STAMPs (Single-cell Transcriptomes Attached to MicroParticles) were resuspended in 1 mL H2O, and loaded on a hemocytometer (Incyto, # DHC-N01). Aliquots of 3,000 beads in 24.6 µL of H2O were amplified with 0.8 µM SMART PCR primer (IDT, 5’-AAG CAG TGG TAT CAA CGC AGA GT-3’) and 2X KAPA HiFi Hotstart Ready Mix (Fisher Scientific, # KK2602) using the following PCR condition: (step 1) 95 °C for 3 min, (step 2) four cycles of: 98 °C for 20 sec, 65 °C for 45 sec, 72 °C for 3 min, (step 3) 10 cycles of: 98 °C for 20 sec, 67 °C for 20 s, 72 °C for 3 min, and (step 4) finally, 72 °C for 5 min.

Purified cDNA with 0.6X SPRI beads were analyzed with High Sensitive DNA kit (Agilent, # 5067-4626), then tagmented and amplified using Nextera XT Library Preparation Kit (Illumina, # FC-131-1096) by manufacturer's manual for sequencing.

*Sequencing*

All libraries were checked its quality with Agilent High Sensitive DNA chips before sequencing.

Each library was diluted at 3 nM for an Illumina HiSeq 3000. We used HiSeq SBS 50 cycle kits for sequence (paired-end): 21-bp for read 1 with Custom Read1 primer (IDT, 5’-GCCTGTCCGCGGAAGCAGTGGTATCAACGCAGAGTAC-3’), 21-bp for read 2, and 8-bp for index 1.

*Preprocessing of Drop-seq data*

Raw sequencing data was preprocessed using the pipeline “Drop-seq Alignment Cookbook” v2.0.0 found at https://github.com/broadinstitute/Drop-seq/releases/ and described in supplementary ref. 4. Briefly, paired-end reads were filtered in order to remove read pairs with any base with quality of less than 10 in both cellular and molecular barcodes. SMART adapters at 5’ end and polyA tails at 3’ end with 6 or more bp were removed from the second pair, and then aligned to the reference human (GRCh38) genome using HISAT2 v2.1.0 [supplementary ref. 5] with the default settings. Uniquely mapped reads were retained and grouped by cell barcode. UMI barcodes with a hamming distance = 1 were merged within each cell, and the number of transcripts of each gene was defined based on the total number of each distinct UMI sequence, for that gene, in a given cell.

*Clustering and Differential Gene Expression analysis for Marker Genes*

All downstream analysis for quality control, clustering, and differential expression were performed using Seurat [supplementary ref. 6] v3.0 R package. Data was initially filtered to remove low quality cells and very low detected genes. We discarded cells with less than 1000 genes detected and with more than 20% of mitochondrial gene content. Genes expressed in less than 3 cells were also removed. Variance stabilizing and normalization was performed using sctransform [supplementary ref. 7] approach adjusting for library size and the percentage of mitochondrial genes in each cell. To cluster the cells, cell distances were defined with 30 first PCs using a graph-based approach implemented in Seurat, that briefly build a K-nearest neighbor (KNN) graph based on euclidean distance in PCA space, with subsequent refining of edge weights using Jacard similarity. Clusters were defined using Seurat default algorithm – a shared nearest neighbor (SNN) graph based on k-nearest neighbors with modularity optimization using original Louvain algorithm. Gene markers of cell clusters were defined using ROC analysis for only genes with at least 0.25 logFC average difference between the clusters. Graphic visualizations of clusters and gene expression were done using Seurat and ggplot2 in R.

*Single-cell trajectory reconstruction*

Reconstruction of cell differentiation trajectories for cell clusters was performed with STREAM (Single cell Trajectories Reconstruction, Exploration and Mapping) [supplementary ref. 8] Python package. The expression data of high-quality cells (9,821 cells) was library size normalized, scaled, natural-log transformed and used as input for STREAM. Data dimension reduction was done with Spectral Embedding (SE) based on most variable genes found with LOESS fitting. Branches representing trajectory structure were learned based on 3 SE dimensions.

Marker genes of each branch were defined by Leaf gene detection analysis implemented in the STREAM package. Initially, the average expression of each gene is calculated for all branches. Based on the average expressions, Z-scores are computed and genes are picked as candidates when greater than 1.5. Then a Kruskal–Wallis H-test is calculated for each candidate to test the difference of gene expression among branches. Finally, the Conover test for multiple comparison is computed for the differentially expressed candidates (Krustal-Wallis p-value < 0.01) between all branches, and genes with Conover p-value < 0.01 are picked as a Leaf gene for that branch. GO enrichment analysis of branch markers were performed for top 100 leaf genes using hypergeometric test with GOstats v2.48.0 R package. Circus plot with GO terms was done using GOplot v1.0.2 R package.

Transition genes were found by performing transition gene analysis implemented in STREAM. The algorithm starts ordering cells based on their inferred pseudotime and splits them in two groups. One group contains 20% of cells closer to the pseudotime start point, and the other is composed of all remaining cells (80%). Gene average expressions are calculated for each group and genes with a log2 fold change greater than 0.25 between groups are picked as candidate genes. Then, Spearman’s rank correlation is computed between inferred pseudotime and gene expression of the candidates. Genes with correlation coefficient above 0.4 are reported as transition genes.

Overrepresentation analysis was performed by using the algorithm MSET [supplementary ref. 9] implemented in R, using 10000 permutations. The list of transcription factors related to trophoblast differentiation used is described in supplementary ref. 10.

Enrichment analysis for placental cell-specific genes.

To characterize the expression profiles of the 19 clusters detected in this work, we analyzed the overlap with cell-specific genes identified by previous single-cell studies. We used two sets of candidate genes. A set of 38 cell-specific gene lists (30 gene each) was drawn from the supplementary table 2 of supplementary ref. 11. These gene lists span both maternal (decidua and blood) and fetal tissues (placenta). Another set of 14 cell-specific gene lists was drawn from the supplementary table 1 of supplementary ref. 12, which also span analogous tissues.

To profile the expression patterns of the 19 clusters detected in this work, we applied the Cell-Specific Expression Analysis (CSEA) as implemented in the pSI R package [supplementary ref. 13]. This approach compares each profile with all the others and identifies genes expressed in one group but not in the others, calculates a score (specificity index, SI) for each gene and attributes a statistical level of significance (pSI). Here, we used the normalized expression matrix to compute cluster-level median gene expression for each gene, which defined 19 cluster profiles. Then, cluster profiles were used to compute SI and pSI for each gene, within each profile. Cluster-specific gene lists were obtained applying the statistical threshold, pSI < 0.05.

Finally, we tested whether cell-specific genes from previous studies are over-represented in our clusters. We used the hypergeometric test and applied the Bonferroni correction for multiple comparisons, considering all the tested gene lists [α = 0.05/ (19× (38+14)) = 5.1×10-5].

In order to compute the similarity of the cells for different developmental stages we used gene lists defined as cell type-specific by Xiang et al. [supplementary ref. 14]. Counts matrix was filtered to keep the same cells and genes used in clustering analysis and the data was normalized using the function NormalizeData from Seurat v.4.0.2 with scale factor equal to 10,000. The similarity to each gene expression program was computed using the function AddModuleScore from Seurat. Basically, the function calculates the average expression levels of each group of cell type-specific genes (programs) on a single cell level, then subtract by the aggregated expression of control feature sets.

Comparisons of gene expression levels for cell type-specific genes among clusters were done by using average expression of each gene and performing pairwise Wilcoxon Rank Sum test. P-values were combined by using the Holm-middle method implemented in the function combinePValues from scran v.1.18.7 R package.

Differential expression analysis for amnion genes was performed by the function FindAllMarkers from Seurat R package using Wilcoxon Rank Sum test with Bonferroni correction.

*PANDA regulatory networks*

Gene regulatory networks were constructed using the pandaR v1.14.0 R package which integrates multiple types of data to infer direct interactions (edges) between TFs and target genes. PANDA initiates with a prior regulatory network that can be built by mapping TF binding sites to the genome, and refines this initial network integrating gene expression data of target genes and TF PPI data. The main idea behind the algorithm is that target genes from a given TF are likely co-expressed, and TFs that interact with each other are more likely to regulate a similar group of target genes. These two assumptions are used to infer the edge weight of each TF-target interaction, and basically reflects the congruence between the regulatory profile of a TF with target gene co-expression. Iteratively the algorithm refine the initial network structure and infer a final consensus regulatory network.

A regulatory network was built for each cluster using as initial network a TF-motif binding map described in [supplementary ref. 15] and downloaded in (https://sites.google.com/a/channing.harvard.edu/kimberlyglass/tools/resources). Gene expression data was library size normalized, scaled, and natural-log transformed. Genes expressed in less than 10% of the cells of each cluster were filtered out. PPI network of TFs was built using STRING database v11.0 (downloaded from https://string-db.org/) and score interactions were divided by 1000 to initiate the PANDA.

Edges with negative weights estimated by PANDA were discarded in order to explore only interactions with greater evidence. The relative importance of individual genes in the network topology was addressed with the betweenness centrality measure calculated using the Igraph v1.2.4 R package. For each cluster, the significance of betweenness values were calculated based on the betweenness probability distribution of 100 random networks constructed with the same number of edges of that cluster. Graphic visualization, gene clusterization, and GO enrichment analysis of gene clusters within networks were performed using Cytoscape v3.7.2 [supplementary ref. 16] and ClueGO v2.5.4.ENRICHMENT/RANKING ANALYSIS [supplementary ref. 17].

**Software and Analysis:**

Analysis pipelines are publicly deposited on Github:https://github.com/paquolalab/placenta_ips

**References**

1. Stirparo, G. G. et al. Integrated analysis of single-cell embryo data yields a unified transcriptome signature for the human preimplantation epiblast. Development dev.158501 (2018) doi:10.1242/dev.158501.
2. Suryawanshi H. et al., A single-cell survey of the human first-trimester placenta and decidua. Sci Adv. 2018 Oct 31;4(10):eaau4788. doi: 10.1126/sciadv.aau4788.
3. Okae, H. et al. Derivation of Human Trophoblast Stem Cells. Cell Stem Cell 22, 50-63.e6 (2018).
4. Macosko, E. Z. et al. Highly Parallel Genome-wide Expression Profiling of Individual Cells Using Nanoliter Droplets. Cell 161, 1202–1214 (2015).
5. Kim, D., Langmead, B. & Salzberg, S. L. HISAT: a fast spliced aligner with low memory requirements. Nat Methods 12, 357–360 (2015).
6. Stuart, T. et al. Comprehensive Integration of Single-Cell Data. Cell 177, 1888-1902.e21 (2019).
7. Hafemeister, C., Satija, R. Normalization and variance stabilization of single-cell RNA-seq data using regularized negative binomial regression. Genome Biol 20, 296 (2019). https://doi.org/10.1186/s13059-019-1874-1
8. Chen, H. et al. Single-cell trajectories reconstruction, exploration and mapping of omics data with STREAM. Nat Commun 10, 1903 (2019).
9. Eisinger, B.E., Saul, M.C., Driessen, T.M. et al. Development of a versatile enrichment analysis tool reveals associations between the maternal brain and mental health disorders, including autism. BMC Neurosci 14, 147 (2013). https://doi.org/10.1186/1471-2202-14-147
10. Krendl, C. et al. GATA2/3-TFAP2A/C transcription factor network couples human pluripotent stem cell differentiation to trophectoderm with repression of pluripotency. Proc. Natl. Acad. Sci. U.S.A. 114, (2017).
11. Vento-Tormo, R. et al. Single-cell reconstruction of the early maternal–fetal interface in humans. Nature 563, 347–353 (2018).
12. Liu, Y. et al. Single-cell RNA-seq reveals the diversity of trophoblast subtypes and patterns of differentiation in the human placenta. Cell Res 28, 819–832 (2018).
13. Xu, X., Wells, A. B., O’Brien, D. R., Nehorai, A. & Dougherty, J. D. Cell Type-Specific Expression Analysis to Identify Putative Cellular Mechanisms for Neurogenetic Disorders. Journal of Neuroscience 34, 1420–1431 (2014).
14. Xiang, L. et al. A developmental landscape of 3D-cultured human pre-gastrulation embryos. Nature 577, 537–542 (2020).
15. Sonawane, A. R. et al. Understanding Tissue-Specific Gene Regulation. Cell Reports 21, 1077–1088 (2017).
16. Shannon, P. et al. Cytoscape: A Software Environment for Integrated Models of Biomolecular Interaction Networks. Genome Res. 13, 2498–2504 (2003).
17. Bindea, G. et al. ClueGO: a Cytoscape plug-in to decipher functionally grouped gene ontology and pathway annotation networks. Bioinformatics 25, 1091–1093 (2009).
