# Supplementary material for: Human archetypal pluripotent stem cells differentiate into trophoblast stem cells via endogenous BMP5/7 induction without transitioning through naive state
Source: Sci Rep. 2024 Feb 8;14:3291. doi: 10.1038/s41598-024-53381-w (PMC10853519; doi:10.1038/s41598-024-53381-w)
Supplement: Supplementary file 2 — Supplementary Information 2. [file 41598_2024_53381_MOESM2_ESM.pdf]

RE: Acknowledgements of authorship changes

Please find a complied document of emails from the authors acknowledging authorship changes after they reviewed the Authorship form. The added authors, Alejandra McCord and Yanhong Wang, signed the pdf. All others agreed to the changes by email.

I, Jennifer Erwin, also acknowledge and agree to all authorship changes.

Sincerely,
Jennifer Erwin

Subject: Re: Fw: Action Required: Acknowledgement of Authorship changes for Scientific Reports Manuscript
Date: Wednesday, January 10, 2024 at 5:11:26 PM Eastern Standard Time
From: Alan Lorenzetti
To: Jennifer Erwin
CC: Tomoyo Sawada
Attachments: Authorship_form_TS_unsigned_AL.docx

Hi Jenny,

I agree with the proposed changes in authorship.

Best regards,
Alan.

On 1/10/24 4:27 PM, Tomoyo Sawada wrote:

From: Jennifer Erwin
Sent: Tuesday, December 26, 2023 9:21:27 PM
To: Alejandra McCord; Yanhong Wang
Cc: ethan.tietze@libd.org; andre.barbosa@libd.org; bhsa83@gmail.com; veronicaeuclydes@alumni.usp.br; heonjin.cho@libd.org; youngkyu.lee@libd.org; Arthur Feltrin; Bailey Spiegelberg; jvandeleemput@gmail.com; pasquale.dicarlo85@gmail.com; Tomoyo Sawada; Gianluca Ursini; kynon.benjamin@libd.org; helena.brentani@gmail.com; Joel Kleinman; Thomas Hyde; Daniel Weinberger, M.D.; Ronald McKay; Joo Heon Shin; Apua Paquola
Subject: Action Required: Acknowledgement of Authorship changes for Scientific Reports Manuscript

Dear co-authors,

The trophoblast stem cell manuscript is nearing publication, and Scientific Reports requires an email acknowledgement of all authors agreeing to the final authorship. In the final revision, additional data and work was performed. To reflect these changes, Alejandra and Yanhong were added as authors and the authorship order changed slightly. Please see the attached document explaining the changes from the April submission.
Please respond to me by email acknowledging you agreement to the changes. Thank you for your contributions to this work.

“This form should be used by authors to request any change in authorship (adding/deleting authors) including changes in corresponding authors. This form should not be used for name changes. Please fully complete all sections. Use black ink and block capitals and provide each author’s full name with the given name first followed by the family name.

- By signing this declaration, all authors guarantee that the order of the authors are in accordance with their scientific contribution, if applicable as different conventions apply per discipline, and that only authors have been added who

made a meaningful contribution to the work.

· Please note, in author collaborations where there is formal agreement for representing the collaboration, it is sufficient for the representative or legal guarantor (usually the corresponding author) to complete and sign the Authorship Change Form on behalf of all authors, **next to the added/removed author(s)**. **(Complete Section 3, followed by Section 6.)**
In author collaborations where there is no formal agreement for representing the collaboration and **there are more than 10 authors**, one may sign for all, provided the signer appends correspondence that attests that each of the authors have agreed to the change **and the added/removed authors sign the form**. **(Complete Section 3, followed by Section 6.)"**

--
Alan Lorenzetti, Ph.D.
Postdoctoral Fellow | Erwin-Paquola Lab
Lieber Institute for Brain Development
Johns Hopkins Medical Campus
alan.lorenzetti@libd.org

Subject: Re: Action Required: Acknowledgement of Authorship changes for Scientific Reports Manuscript
Date: Tuesday, January 2, 2024 at 10:43:25 AM Eastern Standard Time
From: Apua Paquola
To: Jennifer Erwin

I approve these changes.

-----Original Message-----
From: Jennifer Erwin <Jennifer.Erwin@libd.org>
To: Alejandra McCord <Alejandra.McCord@libd.org>, Yanhong Wang <Yanhong.Wang@libd.org>
Cc: ethan.tietze@libd.org <ethan.tietze@libd.org>, andre.barbosa@libd.org <andre.barbosa@libd.org>, bhsa83@gmail.com <bhsa83@gmail.com>, veronicaeuclydes@alumni.usp.br <veronicaeuclydes@alumni.usp.br>, heonjin.cho@libd.org <heonjin.cho@libd.org>, youngkyu.lee@libd.org <youngkyu.lee@libd.org>, Arthur Feltrin <Arthur.Feltrin@libd.org>, Bailey Spiegelberg <Bailey.Spiegelberg@libd.org>, jvandeleemput@gmail.com <jvandeleemput@gmail.com>, pasquale.dicarlo85@gmail.com <pasquale.dicarlo85@gmail.com>, Tomoyo Sawada <Tomoyo.Sawada@libd.org>, Gianluca Ursini <Gianluca.Ursini@libd.org>, kynon.benjamin@libd.org <kynon.benjamin@libd.org>, helena.brentani@gmail.com <helena.brentani@gmail.com>, Joel Kleinman <Joel.Kleinman@libd.org>, Thomas Hyde <Thomas.Hyde@libd.org>, "Daniel Weinberger, M.D." <drweinberger@libd.org>, Ronald McKay <Ronald.McKay@libd.org>, Joo Heon Shin <JooHeon.Shin@libd.org>, Apua Paquola <Apua.Paquola@libd.org>
Subject: Action Required: Acknowledgement of Authorship changes for Scientific Reports Manuscript
Date: 12/26/2023 09:21:27 PM

Dear co-authors,

The trophoblast stem cell manuscript is nearing publication, and Scientific Reports requires an email acknowledgement of all authors agreeing to the final authorship. In the final revision, additional data and work was performed. To reflect these changes, Alejandra and Yanhong were added as authors and the authorship order changed slightly. Please see the attached document explaining the changes from the April submission.
Please respond to me by email acknowledging you agreement to the

changes.Thank you for your contributions to this work.

“This form should be used by authors to request any change in authorship
(adding/deleting authors) including changes in corresponding authors. This form shouldnot be used for name changes. Please fully complete all sections. Use black ink and block capitals and provide each author’s full name with the given name
first followed by the family name.

· By signing this declaration, all authors guarantee that the order of the authors are in accordance with their scientific contribution, if applicable as different conventions apply perdiscipline, and that only authors have been added who made a meaningful contribution to the work.

· Please note, in author collaborations where there is formal agreement for representing the collaboration, it is sufficient for the representative or legal guarantor (usually thecorresponding author) to complete and sign the Authorship Change Form on behalf of all authors, next to the added/removed author(s).(Complete Section 3, followed by Section 6.)

In author collaborations where there is no formal agreement for representing the collaboration and there are more than 10 authors, one may sign for all, provided the signerappends correspondence that attests that each of the authors have agreed to the change and the added/removed authors sign the form. (Complete Section

3, followed
by Section 6.)”

Subject: Re: Action Required: Acknowledgement of Authorship changes for Scientific Reports Manuscript
Date: Tuesday, December 26, 2023 at 10:19:02 PM Eastern Standard Time
From: Arthur Feltrin
To: Jennifer Erwin

I have reviewed the changes and agree with them.

Best regards,

Arthur Feltrin

From: Jennifer Erwin <Jennifer.Erwin@libd.org>
Sent: Tuesday, December 26, 2023 9:21:27 PM
To: Alejandra McCord <Alejandra.McCord@libd.org>; Yanhong Wang <Yanhong.Wang@libd.org>
Cc: ethan.tietze@libd.org <ethan.tietze@libd.org>; andre.barbosa@libd.org <andre.barbosa@libd.org>; bhsa83@gmail.com <bhsa83@gmail.com>; veronicaeuclydes@alumni.usp.br <veronicaeuclydes@alumni.usp.br>; heonjin.cho@libd.org <heonjin.cho@libd.org>; youngkyu.lee@libd.org <youngkyu.lee@libd.org>; Arthur Feltrin <Arthur.Feltrin@libd.org>; Bailey Spiegelberg <Bailey.Spiegelberg@libd.org>; jvandeleemput@gmail.com <jvandeleemput@gmail.com>; pasquale.dicarlo85@gmail.com <pasquale.dicarlo85@gmail.com>; Tomoyo Sawada <Tomoyo.Sawada@libd.org>; Gianluca Ursini <Gianluca.Ursini@libd.org>; kynon.benjamin@libd.org <kynon.benjamin@libd.org>; helena.brentani@gmail.com <helena.brentani@gmail.com>; Joel Kleinman <Joel.Kleinman@libd.org>; Thomas Hyde <Thomas.Hyde@libd.org>; Daniel Weinberger, M.D. <drweinberger@libd.org>; Ronald McKay <Ronald.McKay@libd.org>; Joo Heon Shin <JooHeon.Shin@libd.org>; Apua Paquola <Apua.Paquola@libd.org>
Subject: Action Required: Acknowledgement of Authorship changes for Scientific Reports Manuscript

Dear co-authors,

The trophoblast stem cell manuscript is nearing publication, and Scientific Reports requires an email acknowledgement of all authors agreeing to the final authorship. In the final revision, additional data and work was performed. To reflect these changes, Alejandra and Yanhong were added as authors and the authorship order changed slightly. Please see the attached document explaining the changes from the April submission.
Please respond to me by email acknowledging you agreement to the changes. Thank you for your contributions to this work.

"This form should be used by authors to request any change in authorship (adding/deleting authors) including changes in corresponding authors. This form should not be used for name changes. Please fully complete all sections. Use black ink and block capitals and provide each author's full name with the given name first followed by the family name.

- By signing this declaration, all authors guarantee that the order of the authors are in accordance with their scientific contribution, if applicable as different conventions apply per discipline, and that only authors have been added who made a meaningful contribution to the work.

- Please note, in author collaborations where there is formal agreement for representing the collaboration, it is sufficient for the representative or legal guarantor (usually the corresponding author) to complete and sign the Authorship Change Form on behalf of all authors, **next to the added/removed author(s)**. **(Complete Section 3, followed by Section 6.)**
In author collaborations where there is no formal agreement for representing the collaboration and **there are more than 10 authors**, one may sign for all, provided the signer appends correspondence that attests that each of the authors have agreed to the change **and the added/removed authors sign the form**. **(Complete Section 3, followed by Section 6.)"**

Subject: Re: Action Required: Acknowledgement of Authorship changes for Scientific Reports Manuscript
Date: Tuesday, December 26, 2023 at 9:31:48 PM Eastern Standard Time
From: Bruno Araujo
To: Jennifer Erwin

Hi Jennifer,

I agree with the changes in the authorship of the manuscript.

Happy christmas and happy new year.

Best,
Bruno

On Tue, Dec 26, 2023 at 9:21PM Jennifer Erwin <Jennifer.Erwin@libd.org> wrote:

> Dear co-authors,
>
> The trophoblast stem cell manuscript is nearing publication, and Scientific Reports requires an email acknowledgement of all authors agreeing to the final authorship. In the final revision, additional data and work was performed. To reflect these changes, Alejandra and Yanhong were added as authors and the authorship order changed slightly. Please see the attached document explaining the changes from the April submission.
>
> **Please respond to me by email acknowledging you agreement to the changes.** Thank you for your contributions to this work.
>
> “This form should be used by authors to request any change in authorship (adding/deleting authors) including changes in corresponding authors. This form should not be used for name changes. Please fully complete all sections. Use black ink and block capitals and provide each author’s full name with the given name first followed by the family name.
>
> · By signing this declaration, all authors guarantee that the order of the authors are in accordance with their scientific contribution, if applicable as different conventions apply per discipline, and that only authors have been added who made a meaningful contribution to the work.
>
> · Please note, in author collaborations where there is formal agreement for representing the collaboration, it is sufficient for the representative or legal guarantor (usually the corresponding author) to complete and sign the Authorship Change Form on behalf of all authors, **next to the added/removed author(s)**. **(Complete Section 3, followed by Section 6.)**
>
> In author collaborations where there is no formal agreement for representing the collaboration and **there are more than 10 authors**, one may sign for all, provided the signer appends correspondence that attests that

each of the authors have agreed to the change **and the added/removed authors sign the form**. **(Complete Section 3, followed by Section 6.)"**

Subject: RE: Action Required: Acknowledgement of Authorship changes for Scientific Reports Manuscript
Date: Wednesday, January 10, 2024 at 7:52:45 PM Eastern Standard Time
From: Tietze, Ethan
To: Jennifer Erwin
CC: Tomoyo Sawada

Hi Jenny and Tomoyo,

Hope all is well!

I have reviewed and agree to the changes of authorship attached document.

Thank you,
Ethan

From: Tomoyo Sawada <Tomoyo.Sawada@libd.org>
Sent: Wednesday, January 10, 2024 3:21 PM
To: Tietze, Ethan <ethan.tietze@cuanschutz.edu>
Cc: Jennifer Erwin <Jennifer.Erwin@libd.org>
Subject: Fw: Action Required: Acknowledgement of Authorship changes for Scientific Reports Manuscript
Importance: High

[External Email - Use Caution]

Hi Ethan,

Hope you are doing well!

Our placenta paper is finally being at the last stage before publication.

We made some changes on authorship and would like to get your approval.

Please respond to Jenny (by ccing me) by email acknowledging your agreement to the changes.

Best wishes,

Tomoyo

Tomoyo Sawada, Ph.D. | *Research Scientist*

LIEBER INSTITUTE *for* BRAIN DEVELOPMENT

855 N. Wolfe St, #300 | Baltimore, MD 21205

Web: www.libd.org

Email: Tomoyo.Sawada@libd.org

From: Jennifer Erwin
Sent: Tuesday, December 26, 2023 21:21
To: Alejandra McCord; Yanhong Wang
Cc: ethan.tietze@libd.org; andre.barbosa@libd.org; bhsa83@gmail.com; veronicaeuclydes@alumni.usp.br; heonjin.cho@libd.org; youngkyu.lee@libd.org; Arthur Feltrin; Bailey Spiegelberg; jvandeleemput@gmail.com; pasquale.dicarlo85@gmail.com; Tomoyo Sawada; Gianluca Ursini; kynon.benjamin@libd.org; helena.brentani@gmail.com; Joel Kleinman; Thomas Hyde; Daniel Weinberger, M.D.; Ronald McKay; Joo Heon Shin; Apua Paquola
Subject: Action Required: Acknowledgement of Authorship changes for Scientific Reports Manuscript

Dear co-authors,

The trophoblast stem cell manuscript is nearing publication, and Scientific Reports requires an email acknowledgement of all authors agreeing to the final authorship. In the final revision, additional data and work was performed. To reflect these changes, Alejandra and Yanhong were added as authors and the authorship order changed slightly. Please see the attached document explaining the changes from the April submission.

Please respond to me by email acknowledging you agreement to the changes. Thank you for

your contributions to this work.

“This form should be used by authors to request any change in authorship (adding/deleting authors) including changes in corresponding authors. This form should not be used for name changes. Please fully complete all sections. Use black ink and block capitals and provide each author’s full name with the given name first followed by the family name.

- By signing this declaration, all authors guarantee that the order of the authors are in accordance with their scientific contribution, if applicable as different conventions apply per discipline, and that only authors have been added who made a meaningful contribution to the work.

- Please note, in author collaborations where there is formal agreement for representing the collaboration, it is sufficient for the representative or legal guarantor (usually the corresponding author) to complete and sign the Authorship Change Form on behalf of all authors, **next to the added/removed author(s)**. **(Complete Section 3, followed by Section 6.)**

In author collaborations where there is no formal agreement for representing the collaboration and **there are more than 10 authors**, one may sign for all, provided the signer appends correspondence that attests that each of the authors have agreed to the change **and the added/removed authors sign the form**. **(Complete Section 3, followed by Section 6.)”**

Subject: FW: Action Required: Acknowledgement of Authorship changes for Scientific Reports Manuscript
Date: Monday, January 15, 2024 at 10:10:43 AM Eastern Standard Time
From: Jennifer Erwin

From: Yong Kyu Lee <YongKyu.Lee@libd.org>
Date: Friday, January 5, 2024 at 9:07 AM
To: Jennifer Erwin <Jennifer.Erwin@libd.org>
Subject: Fwd: Action Required: Acknowledgement of Authorship changes for Scientific Reports Manuscript

Happy new year, Jennifer.

I approve these authorship changes.

Thank you.
Yong

Sent from my iPhone

Begin forwarded message:

> **From:** Alejandra McCord <Alejandra.McCord@libd.org>
> **Date:** January 2, 2024 at 10:47:16 AM EST
> **To:** Bailey Spiegelberg <Bailey.Spiegelberg@libd.org>, Joel Kleinman <Joel.Kleinman@libd.org>, Tomoyo Sawada <Tomoyo.Sawada@libd.org>, Jennifer Erwin <Jennifer.Erwin@libd.org>
> **Cc:** Yanhong Wang <Yanhong.Wang@libd.org>, ethan.tietze@libd.org, andre.barbosa@libd.org, bhsa83@gmail.com, veronicaeuclydes@alumni.usp.br, heonjin.cho@libd.org, Arthur Feltrin <Arthur.Feltrin@libd.org>, jvandeleemput@gmail.com, pasquale.dicarlo85@gmail.com, Gianluca Ursini <Gianluca.Ursini@libd.org>, kynon.benjamin@libd.org, helena.brentani@gmail.com, Thomas Hyde <Thomas.Hyde@libd.org>, Ronald McKay <Ronald.McKay@libd.org>, Joo Heon Shin <JooHeon.Shin@libd.org>, Apua Paquola <apua.paquola@libd.org>, "Daniel Weinberger, M.D." <drweinberger@libd.org>, Yong Kyu Lee <YongKyu.Lee@libd.org>
> **Subject: Re: Action Required: Acknowledgement of Authorship changes for Scientific Reports Manuscript**
>
> Hi Jenny,

I approve these authorship changes.

Thanks,

Alejandra

From: Bailey Spiegelberg
Sent: Tuesday, January 2, 2024 10:36:41 AM
To: Joel Kleinman; Tomoyo Sawada; Jennifer Erwin
Cc: Alejandra McCord; Yanhong Wang; ethan.tietze@libd.org; andre.barbosa@libd.org; bhsa83@gmail.com; veronicaeuclydes@alumni.usp.br; heonjin.cho@libd.org; youngkyu.lee@libd.org; Arthur Feltrin; jvandeleemput@gmail.com; pasquale.dicarlo85@gmail.com; Gianluca Ursini; kynon.benjamin@libd.org; helena.brentani@gmail.com; Thomas Hyde; Ronald McKay; Joo Heon Shin; Apua Paquola; Daniel Weinberger, M.D.
Subject: Re: Action Required: Acknowledgement of Authorship changes for Scientific Reports Manuscript

Hi Jenny,

I approve these changes.

Bailey

From: Joel Kleinman
Sent: Wednesday, December 27, 2023 3:46:55 PM
To: Tomoyo Sawada; Jennifer Erwin
Cc: Alejandra McCord; Yanhong Wang; ethan.tietze@libd.org; andre.barbosa@libd.org; bhsa83@gmail.com; veronicaeuclydes@alumni.usp.br; heonjin.cho@libd.org; youngkyu.lee@libd.org; Arthur Feltrin; Bailey Spiegelberg; jvandeleemput@gmail.com; pasquale.dicarlo85@gmail.com; Gianluca Ursini; kynon.benjamin@libd.org; helena.brentani@gmail.com; Thomas Hyde; Ronald McKay; Joo Heon Shin; Apua Paquola; Daniel Weinberger, M.D.
Subject: Re: Action Required: Acknowledgement of Authorship changes for Scientific Reports Manuscript

I approve the authorship changes.

Joel E Klenman

From: Tomoyo Sawada
Sent: Wednesday, December 27, 2023 9:24 AM
To: Jennifer Erwin
Cc: Alejandra McCord; Yanhong Wang; ethan.tietze@libd.org; andre.barbosa@libd.org; bhsa83@gmail.com; veronicaeuclydes@alumni.usp.br; heonjin.cho@libd.org; youngkyu.lee@libd.org; Arthur Feltrin; Bailey Spiegelberg; jvandeleemput@gmail.com; pasquale.dicarlo85@gmail.com; Gianluca Ursini; kynon.benjamin@libd.org; helena.brentani@gmail.com; Joel Kleinman; Thomas Hyde; Ronald McKay; Joo Heon Shin; Apua Paquola; Daniel Weinberger, M.D.
Subject: Re: Action Required: Acknowledgement of Authorship changes for Scientific Reports Manuscript

Hi Jenny,

I acknowledge the changes.

Thank you.

Tomoyo

From: Daniel Weinberger, M.D.
Sent: Wednesday, December 27, 2023 8:31
To: Jennifer Erwin
Cc: Alejandra McCord; Yanhong Wang; ethan.tietze@libd.org; andre.barbosa@libd.org; bhsa83@gmail.com; veronicaeuclydes@alumni.usp.br; heonjin.cho@libd.org; youngkyu.lee@libd.org; Arthur Feltrin; Bailey Spiegelberg; jvandeleemput@gmail.com; pasquale.dicarlo85@gmail.com; Tomoyo Sawada; Gianluca Ursini; kynon.benjamin@libd.org; helena.brentani@gmail.com; Joel Kleinman; Thomas Hyde; Ronald McKay; Joo Heon Shin; Apua Paquola
Subject: Re: Action Required: Acknowledgement of Authorship changes for Scientific

Reports Manuscript

I approve the changes...

Daniel R. Weinberger, M.D.

Sent from my iPad

On Dec 26, 2023, at 9:21 PM, Jennifer Erwin <Jennifer.Erwin@libd.org>

wrote:

Dear co-authors,

The trophoblast stem cell manuscript is nearing publication, and Scientific Reports requires an email acknowledgement of all authors agreeing to the final authorship. In the final revision, additional data and work was performed. To reflect these changes, Alejandra and Yanhong were added as authors and the authorship order changed slightly. Please see the attached document explaining the changes from the April submission.
Please respond to me by email acknowledging you agreement to the changes. Thank you for your contributions to this work.

"This form should be used by authors to request any change in authorship (adding/deleting authors) including changes in corresponding authors. This form should not be used for name changes. Please fully complete all sections. Use black ink and block capitals and provide each author's full name with the given name first followed by the family name.

· By signing this declaration, all authors guarantee that the order of the authors are in accordance with their scientific contribution, if applicable as different conventions apply per discipline, and that only authors have been added who made a meaningful contribution to the work.

· Please note, in author collaborations where there is formal agreement for representing the collaboration, it is sufficient for the representative or legal guarantor (usually the corresponding author) to complete and sign the Authorship Change Form on behalf of all authors, **next to the added/removed author(s). (Complete Section 3, followed by Section 6.)**

In author collaborations where there is no formal agreement for representing the collaboration and **there are more than 10 authors**, one may sign for all, provided the signer appends correspondence that attests that

each of the authors have agreed to the change **and the added/removed authors sign the form. (Complete Section 3, followed by Section 6.)"**

<Authorship form_TS_unsigned.docx>

Subject: Fw: Action Required: Acknowledgement of Authorship changes for Scientific Reports Manuscript
Date: Thursday, January 11, 2024 at 12:15:50 PM Eastern Standard Time
From: Alejandra McCord
To: Jennifer Erwin, Tomoyo Sawada, Bailey Spiegelberg

From: Hyeon Jin Cho <hcho1239@umd.edu>
Sent: Thursday, January 11, 2024 12:10 PM
To: Alejandra McCord
Subject: Re: Action Required: Acknowledgement of Authorship changes for Scientific Reports Manuscript

Hi Alejandra,

Thanks for reaching out. I'm okay with the authorship changes.

Best,

Hyeon Jin Cho
'Jin' is part of my first name
University of Maryland College Park
UMD-NCI Partnership Program

Pronouns: She/her

From: Alejandra McCord <Alejandra.McCord@libd.org>
Date: Wednesday, January 10, 2024 at 2:29 PM
To: "hcho1239@umd.edu" <hcho1239@umd.edu>
Cc: Jennifer Erwin <Jennifer.Erwin@libd.org>, Tomoyo Sawada <Tomoyo.Sawada@libd.org>, Bailey Spiegelberg <bspiege2@jhmi.edu>
Subject: Fw: Action Required: Acknowledgement of Authorship changes for Scientific Reports Manuscript

Hi Hyeon Jin,

Apologies, this was sent to your old email address at first. Please let us know if you are okay with the authorship changes.

Thanks a lot,
Alejandra

From: Jennifer Erwin

Sent: Tuesday, December 26, 2023 9:21 PM
To: Alejandra McCord; Yanhong Wang
Cc: ethan.tietze@libd.org; andre.barbosa@libd.org; bhsa83@gmail.com; veronicaeuclydes@alumni.usp.br; heonjin.cho@libd.org; youngkyu.lee@libd.org; Arthur Feltrin; Bailey Spiegelberg; jvandeleemput@gmail.com; pasquale.dicarlo85@gmail.com; Tomoyo Sawada; Gianluca Ursini; kynon.benjamin@libd.org; helena.brentani@gmail.com; Joel Kleinman; Thomas Hyde; Daniel Weinberger, M.D.; Ronald McKay; Joo Heon Shin; Apua Paquola
Subject: Action Required: Acknowledgement of Authorship changes for Scientific Reports Manuscript

Dear co-authors,

The trophoblast stem cell manuscript is nearing publication, and Scientific Reports requires an email acknowledgement of all authors agreeing to the final authorship. In the final revision, additional data and work was performed. To reflect these changes, Alejandra and Yanhong were added as authors and the authorship order changed slightly. Please see the attached document explaining the changes from the April submission.
Please respond to me by email acknowledging you agreement to the changes. Thank you for your contributions to this work.

"This form should be used by authors to request any change in authorship (adding/deleting authors) including changes in corresponding authors. This form should not be used for name changes. Please fully complete all sections. Use black ink and block capitals and provide each author's full name with the given name first followed by the family name.

- By signing this declaration, all authors guarantee that the order of the authors are in accordance with their scientific contribution, if applicable as different conventions apply per discipline, and that only authors have been added who made a meaningful contribution to the work.

- Please note, in author collaborations where there is formal agreement for representing the collaboration, it is sufficient for the representative or legal guarantor (usually the corresponding author) to complete and sign the Authorship Change Form on behalf of all authors, **next to the added/removed author(s)**. **(Complete Section 3, followed by Section 6.)**

In author collaborations where there is no formal agreement for representing the collaboration and **there are more than 10 authors**, one may sign for all, provided the signer appends correspondence that attests that each of the authors have agreed to the change **and the added/removed authors sign the form**. **(Complete Section 3, followed by Section 6.)"**

Subject: Re: Action Required: Acknowledgement of Authorship changes for Scientific Reports Manuscript
Date: Monday, January 8, 2024 at 2:37:50 PM Eastern Standard Time
From: Joo Heon Shin
To: Jennifer Erwin

Hi Jenny,

Sorry I was out of office.
I approve for the change of the authorship.

Joo Heon

From: Jennifer Erwin <Jennifer.Erwin@libd.org>
Date: Tuesday, December 26, 2023 at 9:21 PM
To: Alejandra McCord <Alejandra.McCord@libd.org>, Yanhong Wang <Yanhong.Wang@libd.org>
Cc: "ethan.tietze@libd.org" <ethan.tietze@libd.org>, "andre.barbosa@libd.org" <andre.barbosa@libd.org>, "bhsa83@gmail.com" <bhsa83@gmail.com>, "veronicaeuclydes@alumni.usp.br" <veronicaeuclydes@alumni.usp.br>, "heonjin.cho@libd.org" <heonjin.cho@libd.org>, "youngkyu.lee@libd.org" <youngkyu.lee@libd.org>, Arthur Feltrin <Arthur.Feltrin@libd.org>, Bailey Spiegelberg <Bailey.Spiegelberg@libd.org>, "jvandeleemput@gmail.com" <jvandeleemput@gmail.com>, "pasquale.dicarlo85@gmail.com" <pasquale.dicarlo85@gmail.com>, Tomoyo Sawada <Tomoyo.Sawada@libd.org>, Gianluca Ursini <Gianluca.Ursini@libd.org>, "kynon.benjamin@libd.org" <kynon.benjamin@libd.org>, "helena.brentani@gmail.com" <helena.brentani@gmail.com>, Joel Kleinman <Joel.Kleinman@libd.org>, Thomas Hyde <Thomas.Hyde@libd.org>, "Daniel Weinberger, M.D." <drweinberger@libd.org>, Ronald McKay <Ronald.McKay@libd.org>, Joo Heon Shin <JooHeon.Shin@libd.org>, Apua Paquola <Apua.Paquola@libd.org>
Subject: Action Required: Acknowledgement of Authorship changes for Scientific Reports Manuscript

Dear co-authors,

The trophoblast stem cell manuscript is nearing publication, and Scientific Reports requires an email acknowledgement of all authors agreeing to the final authorship. In the final revision, additional data and work was performed. To reflect these changes, Alejandra and Yanhong were added as authors and the authorship order changed slightly. Please see the attached document explaining the changes from the April submission.
Please respond to me by email acknowledging you agreement to the changes. Thank you for your contributions to this work.

“This form should be used by authors to request any change in authorship (adding/deleting authors) including changes in corresponding authors. This form should not be used for name changes. Please fully complete all sections. Use black ink and block capitals and provide each author’s full name with the given name first followed by the family name.

- By signing this declaration, all authors guarantee that the order of the authors are in accordance with their scientific contribution, if applicable as different conventions apply per discipline, and that only authors have been added who made a meaningful contribution to the work.

- Please note, in author collaborations where there is formal agreement for representing the collaboration, it is sufficient for the representative or legal guarantor (usually the corresponding author) to complete and sign the Authorship Change Form on behalf of all authors, **next to the added/removed author(s)**. **(Complete Section 3, followed by Section 6.)**
  In author collaborations where there is no formal agreement for representing the collaboration and **there are more than 10 authors**, one may sign for all, provided the signer appends correspondence that attests that each of the authors have agreed to the change **and the added/removed authors sign the form**. **(Complete Section 3, followed by Section 6.)"**

Subject: Re: Action Required: Acknowledgement of Authorship changes for Scientific Reports Manuscript
Date: Wednesday, December 27, 2023 at 11:29:22 AM Eastern Standard Time
From: Joyce van de Leemput
To: Jennifer Erwin

Hi Jennifer,
I approve the changes.
Can you make sure my name is spelled correctly on the manuscript? Only the L should be capitalized in my last name.

Thanks.
Joyce

On Tue, Dec 26, 2023, 9:21 PM Jennifer Erwin <Jennifer.Erwin@libd.org> wrote:

> Dear co-authors,
>
> The trophoblast stem cell manuscript is nearing publication, and Scientific Reports requires an email acknowledgement of all authors agreeing to the final authorship. In the final revision, additional data and work was performed. To reflect these changes, Alejandra and Yanhong were added as authors and the authorship order changed slightly. Please see the attached document explaining the changes from the April submission.
>
> **Please respond to me by email acknowledging you agreement to the changes.** Thank you for your contributions to this work.
>
> "This form should be used by authors to request any change in authorship (adding/deleting authors) including changes in corresponding authors. This form should not be used for name changes. Please fully complete all sections. Use black ink and block capitals and provide each author's full name with the given name first followed by the family name.
>
> · By signing this declaration, all authors guarantee that the order of the authors are in accordance with their scientific contribution, if applicable as different conventions apply per discipline, and that only authors have been added who made a meaningful contribution to the work.
>
> · Please note, in author collaborations where there is formal agreement for representing the collaboration, it is sufficient for the representative or legal guarantor (usually the corresponding author) to complete and sign the Authorship Change Form on behalf of all authors, **next to the added/removed author(s)**. **(Complete Section 3, followed by Section 6.)**
>
> In author collaborations where there is no formal agreement for representing the collaboration and **there are more than 10 authors**, one may sign for all, provided the signer appends correspondence that attests that each of the authors have agreed to the change **and the added/removed authors sign the form**.

(Complete Section 3, followed by Section 6.)"

Subject: Re: Action Required: Acknowledgement of Authorship changes for Scientific Reports Manuscript
Date: Friday, December 29, 2023 at 12:27:31 PM Eastern Standard Time
From: Kynon Jade Benjamin
To: Jennifer Erwin

Dear Jenny,

I acknowledge and agree with the submission changes.

Sincerely,
Kynon

Kynon Jade Benjamin, PhD (*he/him*)
Postdoctoral Fellow
LIBD-PDA President

Lieber Institute for Brain Development
Johns Hopkins School of Medicine
855 N Wolfe St, #300
Baltimore, MD 21205
jade.benjamin@libd.org
(317) 292-8067

From: Jennifer Erwin
Sent: Tuesday, December 26, 2023 9:25:59 PM
To: Kynon Jade Benjamin
Subject: Action Required: Acknowledgement of Authorship changes for Scientific Reports Manuscript

Dear co-authors,

The trophoblast stem cell manuscript is nearing publication, and Scientific Reports requires an email acknowledgement of all authors agreeing to the final authorship. In the final revision, additional data and work was performed. To reflect these changes, Alejandra and Yanhong were added as authors and the authorship order changed slightly. Please see the attached document explaining the changes from the April submission.
Please respond to me by email acknowledging you agreement to the changes. Thank you for your contributions to this work.

"This form should be used by authors to request any change in authorship (adding/deleting authors) including changes in corresponding authors. This form should not be used for name changes. Please fully complete all sections. Use black ink and block capitals and provide each author's full name with the given name first followed by the family name.

- By signing this declaration, all authors guarantee that the order of the authors are in accordance with their scientific contribution, if applicable as different conventions apply per discipline, and that only authors have been added who made a meaningful contribution to the work.

- Please note, in author collaborations where there is formal agreement for representing the collaboration, it is sufficient for the representative or legal guarantor (usually the corresponding author) to complete and sign the Authorship Change Form on behalf of all authors, **next to the added/removed author(s)**. **(Complete Section 3, followed by Section 6.)**
In author collaborations where there is no formal agreement for representing the collaboration and **there are more than 10 authors**, one may sign for all, provided the signer appends correspondence that attests that each of the authors have agreed to the change **and the added/removed authors sign the form**. **(Complete Section 3, followed by Section 6.)"**

Subject: Re: Fw: Action Required: Acknowledgement of Authorship changes for Scientific Reports Manuscript
Date: Thursday, December 28, 2023 at 11:33:54 AM Eastern Standard Time
From: André Barbosa
To: Jennifer Erwin

Hi Jenny,
Happy holidays, I hope you and baby are having the best time.

I agree with the proposed new authorship and the proposed change in corresponding author.

Best Regards

On Wed, Dec 27, 2023 at 6:49PM Jennifer Erwin <Jennifer.Erwin@libd.org> wrote:

Hi Andre,
Happy holidays. Please see email thread below. Hope all is well.
Best,
Jenny

From: Jennifer Erwin
Sent: Tuesday, December 26, 2023 9:21:27 PM
To: Alejandra McCord; Yanhong Wang
Cc: ethan.tietze@libd.org; andre.barbosa@libd.org; bhsa83@gmail.com; veronicaeuclydes@alumni.usp.br; heonjin.cho@libd.org; youngkyu.lee@libd.org; Arthur Feltrin; Bailey Spiegelberg; jvandeleemput@gmail.com; pasquale.dicarlo85@gmail.com; Tomoyo Sawada; Gianluca Ursini; kynon.benjamin@libd.org; helena.brentani@gmail.com; Joel Kleinman; Thomas Hyde; Daniel Weinberger, M.D.; Ronald McKay; Joo Heon Shin; Apua Paquola
Subject: Action Required: Acknowledgement of Authorship changes for Scientific Reports Manuscript

Dear co-authors,

The trophoblast stem cell manuscript is nearing publication, and Scientific Reports requires an email acknowledgement of all authors agreeing to the final authorship. In the final revision, additional data and work was performed. To reflect these changes, Alejandra and Yanhong were added as authors and the authorship order changed slightly. Please see the attached document explaining the changes from the April submission.

Please respond to me by email acknowledging you agreement to the changes. Thank you for your contributions to this work.

"This form should be used by authors to request any change in authorship (adding/deleting authors) including changes in corresponding authors. This form should not be used for name changes. Please fully complete all sections. Use black ink and block capitals and provide each author's full name with the given

name first followed by the family name.

· By signing this declaration, all authors guarantee that the order of the authors are in accordance with their scientific contribution, if applicable as different conventions apply per discipline, and that only authors have been added who made a meaningful contribution to the work.

· Please note, in author collaborations where there is formal agreement for representing the collaboration, it is sufficient for the representative or legal guarantor (usually the corresponding author) to complete and sign the Authorship Change Form on behalf of all authors, **next to the added/removed author(s)**. **(Complete Section 3, followed by Section 6.)**

In author collaborations where there is no formal agreement for representing the collaboration and **there are more than 10 authors**, one may sign for all, provided the signer appends correspondence that attests that each of the authors have agreed to the change **and the added/removed authors sign the form**. **(Complete Section 3, followed by Section 6.)"**

--
Andre Rocha Barbosa, PhD

Subject: Re: Action Required: Acknowledgement of Authorship changes for Scientific Reports Manuscript
Date: Wednesday, December 27, 2023 at 5:26:26 AM Eastern Standard Time
From: Gianluca Ursini
To: Jennifer Erwin

Dear Jennifer,

I agree with the authorship changes.

If needed, my orcid id is: 0000-0002-4733-5196

Thank you
Luca

Gianluca Ursini, MD, PhD
Investigator, Lieber Institute for Brain Development
Assistant Professor of Psychiatry and Behavioral Sciences, Johns Hopkins University
855 North Wolfe Street, suite 300, office 383 - Baltimore (MD)
Tel. +1.4109551131 (office); +1.4439342997 (mobile)
gianluca.ursini@libd.org
gursini1@jhmi.edu
www.libd.org/team/gianluca-ursini/

On Dec 27, 2023, at 3:21AM, Jennifer Erwin <Jennifer.Erwin@libd.org> wrote:

Dear co-authors,

The trophoblast stem cell manuscript is nearing publication, and Scientific Reports requires an email acknowledgement of all authors agreeing to the final authorship. In the final revision, additional data and work was performed. To reflect these changes, Alejandra and Yanhong were added as authors and the authorship order changed slightly. Please see the attached document explaining the changes from the April submission.
Please respond to me by email acknowledging you agreement to the changes. Thank you for your contributions to this work.

"This form should be used by authors to request any change in authorship (adding/deleting authors) including changes in corresponding authors. This form should not be used for name changes. Please fully complete all sections. Use black ink and block capitals and provide each author's full name with the given name first followed by the family name.

· By signing this declaration, all authors guarantee that the order of the authors are in accordance with their scientificcontribution, if applicable as different

conventions apply per discipline, and that only authors have been added who made ameaningful contribution to the work.

- Please note, in author collaborations where there is formal agreement for representing the collaboration, it is sufficient for the representative or legal guarantor (usually the corresponding author) to complete and sign the Authorship Change Form on behalf of all authors, **next to the added/removed author(s)**. **(Complete Section 3, followed by Section 6.)**
In author collaborations where there is no formal agreement for representing the collaboration and **there are more than 10 authors**,one may sign for all, provided the signer appends correspondence that attests that each of the authors have agreed to thechange **and the added/removed authors sign the form**. **(Complete Section 3, followed by Section 6.)"**

<Authorship form_TS_unsigned.docx>

Subject: Re: Action Required: Acknowledgement of Authorship changes for Scientific Reports Manuscript
Date: Wednesday, December 27, 2023 at 5:02:39 AM Eastern Standard Time
From: Pasquale Di Carlo
To: Jennifer Erwin

Dear Jennifer,
I acknowledge the authors' list changes.

Best,
Pasquale Di Carlo

Il Mer 27 Dic 2023, 03:21 Jennifer Erwin <Jennifer.Erwin@libd.org> ha scritto:

> Dear co-authors,
>
> The trophoblast stem cell manuscript is nearing publication, and Scientific Reports requires an email acknowledgement of all authors agreeing to the final authorship. In the final revision, additional data and work was performed. To reflect these changes, Alejandra and Yanhong were added as authors and the authorship order changed slightly. Please see the attached document explaining the changes from the April submission.
>
> **Please respond to me by email acknowledging you agreement to the changes.** Thank you for your contributions to this work.
>
> "This form should be used by authors to request any change in authorship (adding/deleting authors) including changes in corresponding authors. This form should not be used for name changes. Please fully complete all sections. Use black ink and block capitals and provide each author's full name with the given name first followed by the family name.
>
> · By signing this declaration, all authors guarantee that the order of the authors are in accordance with their scientific contribution, if applicable as different conventions apply per discipline, and that only authors have been added who made a meaningful contribution to the work.
>
> · Please note, in author collaborations where there is formal agreement for representing the collaboration, it is sufficient for the representative or legal guarantor (usually the corresponding author) to complete and sign the Authorship Change Form on behalf of all authors, **next to the added/removed author(s)**. **(Complete Section 3, followed by Section 6.)**
>
> In author collaborations where there is no formal agreement for representing the collaboration and **there are more than 10 authors**, one may sign for all, provided the signer appends correspondence that attests that each of the authors have agreed to the change **and the added/removed authors sign the form**. **(Complete Section 3, followed by Section 6.)"**

Subject: Re: Action Required: Acknowledgement of Authorship changes for Scientific Reports Manuscript
Date: Saturday, January 6, 2024 at 5:58:54 PM Eastern Standard Time
From: Helena Brentani
To: Jennifer Erwin
CC: Alejandra McCord, Yanhong Wang, ethan.tietze@libd.org, andre.barbosa@libd.org, bhsa83@gmail.com, veronicaeuclydes@alumni.usp.br, heonjin.cho@libd.org, youngkyu.lee@libd.org, Arthur Feltrin, Bailey Spiegelberg, jvandeleemput@gmail.com, pasquale.dicarlo85@gmail.com, Tomoyo Sawada, Gianluca Ursini, kynon.benjamin@libd.org, Joel Kleinman, Thomas Hyde, Daniel Weinberger, M.D., Ronald McKay, Joo Heon Shin, Apua Paquola

Hi Jennifer
I was out of office, sorry for my late answer
I approve the changes
thanks
Helena

On Tue, Dec 26, 2023 at 11:21PM Jennifer Erwin <Jennifer.Erwin@libd.org> wrote:

> Dear co-authors,
>
> The trophoblast stem cell manuscript is nearing publication, and Scientific Reports requires an email acknowledgement of all authors agreeing to the final authorship. In the final revision, additional data and work was performed. To reflect these changes, Alejandra and Yanhong were added as authors and the authorship order changed slightly. Please see the attached document explaining the changes from the April submission.
>
> **Please respond to me by email acknowledging you agreement to the changes.** Thank you for your contributions to this work.
>
> “This form should be used by authors to request any change in authorship (adding/deleting authors) including changes in corresponding authors. This form should not be used for name changes. Please fully complete all sections. Use black ink and block capitals and provide each author’s full name with the given name first followed by the family name.
>
> · By signing this declaration, all authors guarantee that the order of the authors are in accordance with their scientific contribution, if applicable as different conventions apply per discipline, and that only authors have been added who made a meaningful contribution to the work.
>
> · Please note, in author collaborations where there is formal agreement for representing the collaboration, it is sufficient for the representative or legal guarantor (usually the corresponding author) to complete and sign the Authorship Change Form on behalf of all authors, **next to the added/removed author(s)**. **(Complete Section 3, followed by Section 6.)**

> In author collaborations where there is no formal agreement for representing the collaboration and **there are more than 10 authors**, one may sign for all, provided the signer appends correspondence that attests that each of the authors have agreed to the change **and the added/removed authors sign the form**. **(Complete Section 3, followed by Section 6.)"**

--

Helena Brentani MD PhD-
Professora doutora do departamento de Psiquiatria da FMUSP
Departamento de Psiquiatria da FMUSP
Rua Dr Ovidio Pires de Campos,785
CEP05403-010 São Paulo, SP
Caixa Postal n 3671
Fone 2661-7898.
helena.brentani@gmail.com

Subject: Re: Action Required: Acknowledgement of Authorship changes for Scientific Reports Manuscript
Date: Wednesday, December 27, 2023 at 11:42:07 AM Eastern Standard Time
From: Ronald McKay
To: Tomoyo Sawada, Jennifer Erwin
CC: Alejandra McCord, Yanhong Wang, ethan.tietze@libd.org, andre.barbosa@libd.org, bhsa83@gmail.com, veronicaeuclydes@alumni.usp.br, heonjin.cho@libd.org, youngkyu.lee@libd.org, Arthur Feltrin, Bailey Spiegelberg, jvandeleemput@gmail.com, pasquale.dicarlo85@gmail.com, Gianluca Ursini, kynon.benjamin@libd.org, helena.brentani@gmail.com, Joel Kleinman, Thomas Hyde, Joo Heon Shin, Apua Paquola, Daniel Weinberger, M.D.

Hi Jenny,

I approve these changes.
Best,

Ron

On Dec 27, 2023, at 9:24AM, Tomoyo Sawada <Tomoyo.Sawada@libd.org> wrote:

Hi Jenny,

I acknowledge the changes.
Please use the attached form. I was duplicated in the author list (section 4), and I corrected it.

Thank you.
Tomoyo

From: Daniel Weinberger, M.D.
Sent: Wednesday, December 27, 2023 8:31
To: Jennifer Erwin
Cc: Alejandra McCord; Yanhong Wang; ethan.tietze@libd.org; andre.barbosa@libd.org; bhsa83@gmail.com; veronicaeuclydes@alumni.usp.br; heonjin.cho@libd.org; youngkyu.lee@libd.org; Arthur Feltrin; Bailey Spiegelberg; jvandeleemput@gmail.com; pasquale.dicarlo85@gmail.com; Tomoyo Sawada; Gianluca Ursini; kynon.benjamin@libd.org; helena.brentani@gmail.com; Joel Kleinman; Thomas Hyde; Ronald McKay; Joo Heon Shin; Apua Paquola
Subject: Re: Action Required: Acknowledgement of Authorship changes for Scientific Reports Manuscript

I approve the changes…

Daniel R. Weinberger, M.D.

Sent from my iPad

On Dec 26, 2023, at 9:21 PM, Jennifer Erwin <Jennifer.Erwin@libd.org> wrote:

Dear co-authors,

The trophoblast stem cell manuscript is nearing publication, and Scientific Reports requires an email acknowledgement of all authors agreeing to the final authorship. In the final revision, additional data and work was performed. To reflect these changes, Alejandra and Yanhong were added as authors and the authorship order changed slightly. Please see the attached document explaining the changes from the April submission.
Please respond to me by email acknowledging you agreement to the changes. Thank you for your contributions to this work.

“This form should be used by authors to request any change in authorship (adding/deleting authors) including changes in corresponding authors. This form should not be used for name changes. Please fully complete all sections. Use black ink and block capitals and provide each author’s full name with the given name first followed by the family name.

· By signing this declaration, all authors guarantee that the order of the authors are in accordance with their scientific contribution, if applicable as different conventions apply perdiscipline, and that only authors have been added who made a meaningful contribution to thework.

· Please note, in author collaborations where there is formal agreement for representing the collaboration, it is sufficient for the representative or legal guarantor (usually the corresponding author) to complete and sign the Authorship Change Form on behalf of all authors, **next to the added/removed author(s). (Complete Section 3, followed by Section 6.)**

In author collaborations where there is no formal agreement for representing thecollaboration and **there are more than 10 authors**, one may sign for all, provided thesigner appends correspondence that attests that each of the authors have agreed tothe change **and the added/removed authors sign the form. (Complete Section 3, followed by Section 6.)”**

<Authorship form_TS_unsigned.docx>

<Authorship form_TS_unsigned_ts.docx>

Subject: Re: Action Required: Acknowledgement of Authorship changes for Scientific Reports Manuscript
Date: Wednesday, December 27, 2023 at 9:05:08 AM Eastern Standard Time
From: Thomas Hyde
To: Jennifer Erwin

I approve of the changes.

Tom

From: Jennifer Erwin <Jennifer.Erwin@libd.org>
Date: Tuesday, December 26, 2023 at 9:21 PM
To: Alejandra McCord <Alejandra.McCord@libd.org>, Yanhong Wang <Yanhong.Wang@libd.org>
Cc: "ethan.tietze@libd.org" <ethan.tietze@libd.org>, "andre.barbosa@libd.org" <andre.barbosa@libd.org>, "bhsa83@gmail.com" <bhsa83@gmail.com>, "veronicaeuclydes@alumni.usp.br" <veronicaeuclydes@alumni.usp.br>, "heonjin.cho@libd.org" <heonjin.cho@libd.org>, "youngkyu.lee@libd.org" <youngkyu.lee@libd.org>, Arthur Feltrin <Arthur.Feltrin@libd.org>, Bailey Spiegelberg <Bailey.Spiegelberg@libd.org>, "jvandeleemput@gmail.com" <jvandeleemput@gmail.com>, "pasquale.dicarlo85@gmail.com" <pasquale.dicarlo85@gmail.com>, Tomoyo Sawada <Tomoyo.Sawada@libd.org>, Gianluca Ursini <Gianluca.Ursini@libd.org>, "kynon.benjamin@libd.org" <kynon.benjamin@libd.org>, "helena.brentani@gmail.com" <helena.brentani@gmail.com>, Joel Kleinman <Joel.Kleinman@libd.org>, Thomas Hyde <Thomas.Hyde@libd.org>, Danny Weinberger <drweinberger@libd.org>, Ronald McKay <Ronald.McKay@libd.org>, Joo Heon Shin <JooHeon.Shin@libd.org>, Apua Paquola <Apua.Paquola@libd.org>
Subject: Action Required: Acknowledgement of Authorship changes for Scientific Reports Manuscript

Dear co-authors,

The trophoblast stem cell manuscript is nearing publication, and Scientific Reports requires an email acknowledgement of all authors agreeing to the final authorship. In the final revision, additional data and work was performed. To reflect these changes, Alejandra and Yanhong were added as authors and the authorship order changed slightly. Please see the attached document explaining the changes from the April submission.
Please respond to me by email acknowledging you agreement to the changes. Thank you for your contributions to this work.

"This form should be used by authors to request any change in authorship (adding/deleting authors) including changes in corresponding authors. This form should not be used for name changes. Please fully complete all sections. Use black ink and block capitals and provide each author's full name with the given name first followed by the family name.

- By signing this declaration, all authors guarantee that the order of the authors are in accordance with their scientific contribution, if applicable as different conventions apply per discipline, and that only authors have been added who made a meaningful contribution to the work.

- Please note, in author collaborations where there is formal agreement for representing the collaboration, it is sufficient for the representative or legal guarantor (usually the corresponding author) to complete and sign the Authorship Change Form on behalf of all authors, **next to the added/removed author(s)**. **(Complete Section 3, followed by Section 6.)**
  In author collaborations where there is no formal agreement for representing the collaboration and **there are more than 10 authors**, one may sign for all, provided the signer appends correspondence that attests that each of the authors have agreed to the change **and the added/removed authors sign the form**. **(Complete Section 3, followed by Section 6.)"**

Subject: FW: Action Required: Acknowledgement of Authorship changes for Scientific Reports Manuscript
Date: Monday, January 15, 2024 at 10:25:46 AM Eastern Standard Time
From: Jennifer Erwin
To: Jennifer Erwin

From: Veronica Euclydes <veronicaeuclydes@alumni.usp.br>
Date: Wednesday, December 27, 2023 at 12:46 PM
To: Jennifer Erwin <Jennifer.Erwin@libd.org>
Subject: Re: Action Required: Acknowledgement of Authorship changes for Scientific Reports Manuscript

Hi Jennifer,
I approve the changes.

Thanks.
Veronica

On Tue, Dec 26, 2023, 9:21 PM Jennifer Erwin <Jennifer.Erwin@libd.org> wrote:

> Dear co-authors,
>
> The trophoblast stem cell manuscript is nearing publication, and Scientific Reports requires an email acknowledgement of all authors agreeing to the final authorship. In the final revision, additional data and work was performed. To reflect these changes, Alejandra and Yanhong were added as authors and the authorship order changed slightly. Please see the attached document explaining the changes from the April submission.
> **Please respond to me by email acknowledging you agreement to the changes.** Thank you for your contributions to this work.
>
> “This form should be used by authors to request any change in authorship (adding/deleting authors) including changes in corresponding authors. This form should not be used for name changes. Please fully complete all sections. Use black ink and block capitals and provide each author’s full name with the given name first followed by the family name.
>
> · By signing this declaration, all authors guarantee that the order of the authors are in accordance with their scientific contribution, if applicable as different conventions apply per discipline, and that only authors have been added who made a meaningful contribution to the work.
>
> · Please note, in author collaborations where there is formal agreement for representing the collaboration, it is sufficient for the representative or legal guarantor (usually the corresponding author) to complete and sign the Authorship Change Form on behalf of all authors, **next to the added/removed author(s)**. **(Complete Section 3, followed by Section 6.)**
>
> In author collaborations where there is no formal agreement for representing the collaboration and **there are more than 10 authors**, one may sign for all, provided the signer appends correspondence that

attests that each of the authors have agreed to the change **and the added/removed authors sign the form**. **(Complete Section 3, followed by Section 6.)"**
