# Supplementary material for: Human archetypal pluripotent stem cells differentiate into trophoblast stem cells via endogenous BMP5/7 induction without transitioning through naive state
Source: Sci Rep. 2024 Feb 8;14:3291. doi: 10.1038/s41598-024-53381-w (PMC10853519; doi:10.1038/s41598-024-53381-w)
Supplement: Supplementary file 3 — Supplementary Information 3. [file 41598_2024_53381_MOESM3_ESM.pdf]

SPRINGER NATURE

Change of authorship request form - Journals (pre-acceptance)

Important information. Please read.

- This form should be used by authors to request any change in authorship (adding/deleting authors) including changes in corresponding authors. This form should not be used for name changes. Please fully complete all sections. Use black ink and block capitals and provide each author's full name with the given name first followed by the family name.

- By signing this declaration, all authors guarantee that the order of the authors are in accordance with their scientific contribution, if applicable as different conventions apply per discipline, and that only authors have been added who made a meaningful contribution to the work.

- Please note, in author collaborations where there is formal agreement for representing the collaboration, it is sufficient for the representative or legal guarantor (usually the corresponding author) to complete and sign the Authorship Change Form on behalf of all authors, **next to the added/removed author(s). (Complete Section 3, followed by Section 6.)**
  In author collaborations where there is no formal agreement for representing the collaboration and **there are more than 10 authors**, one may sign for all, provided the signer appends correspondence that attests that each of the authors have agreed to the change **and the added/removed authors sign the form. (Complete Section 3, followed by Section 6.)**

- Please note, we cannot investigate or mediate any authorship disputes. If you are unable to obtain agreement from all authors (including those who you wish to be removed) you must refer the matter to your institution(s) for investigation. Please inform us if you need to do this.

- If you are not able to return a fully completed form within **30 days** of the date that it was sent to the author requesting the change, we may have to withdraw your manuscript. We cannot publish manuscripts where authorship has not been agreed by all authors (including those who have been removed).

- Incomplete forms will be rejected.

- Please return/upload this form, fully completed, to the Journals Editorial Office. The Journal and/or Publisher will consider the information you have provided to decide whether to approve the proposed change in authorship. We may decide to contact your institution for more information or undertake a further investigation, if appropriate, before making a final decision.

SPRINGER NATURE

Change of authorship request form - Journals (pre-acceptance)

Section 1: Please provide the current title of manuscript

Manuscript ID no.: 5662b14c-4690-445f-a93e-81323917c826 v2.0

Title: **A single-cell trajectory of human archetypal pluripotent stem cell differentiation to trophoblast stem cells reveals induction of endogenous BMP5/7 and GATA3 without transitioning through a naive state**

Section 2: Please provide the previous authorship, in the order shown on the manuscript before the changes were introduced. Please indicate the corresponding author by adding (CA) behind the name.

	First name(s)	Family name	ORCID or SCOPUS id, if available
1st author	Ethan	Tietze	
2nd author	Andre	Barbosa	
3rd author	Bruno	Araujo	
4th author	Veronica	Euclydes	
5th author	Hyeon Jin	Cho	
6th author	Yong kyu	Lee	
7th author	Arthur	Feltrin	
8th author	Bailey	Spiegelberg	
9th author	Alan	Lorenzetti	
10th author	Joyce	Van de Leemput	

Please use an additional sheet if there are more than 10 authors.

SPRINGER NATURE Springer Nature is one of the world's leading global research, educational and professional publishers, created in May 2015 through the combination of Nature Publishing Group, Palgrave Macmillan, Macmillan Education and Springer Science+Business Media.

Change of authorship request form - Journals (pre-acceptance)

Section 2: Please provide the previous authorship, in the order shown on the manuscript before the changes were introduced. Please indicate the corresponding author by adding (CA) behind the name.

	First name(s)	Family name	ORCID or SCOPUS id, if available
11 author	Pasquale	Di Carlo	
12 author	Tomoyo	Sawada	
13 author	Gianluca	Ursini	
14 author	Kynon	Benjamin	
15th author	Helena	Brentani	
16th author	Joel	Kleinman	
17th author	Thomas	Hyde	
18th author	Daniel	Weinberger	
19th author	Ronald	McKay	
20th author	Joo Heon	Shin	
21 author	Apua	Paquola	
22 author	Jennifer	Erwin (CA)	

SPRINGER NATURE

Change of authorship request form - Journals (pre-acceptan

Section 3: Please provide a justification for change. Please use this section to explain your reasons for changing the authorship of your manuscript, e.g. what necessitated the change in authorship? Please refer to the (journal) policy pages for more information about authorship. Please explain why omitted authors were not originally included and/or why authors were removed on the submitted manuscript.

For the revision, experiments to generate additional TS lines and bulk RNAseq data, and additional writing and analysis were performed. These experiments and analysis were performed by Bailey Spiegelberg, Yanhong Wang, Alejandra McCord and Tomoyo Sawada in addition to previously included authors. Therefore, Bailey Spiegelberg was moved up in authorship order, Yanhong Wang and Alejandra McCord were added as authors. Tomoyo Sawada was moved to a more senior position (3rd from last) and was added as an additional corresponding author because she helped oversee the analysis and paper submission for the revision.

Section 4: Proposed new authorship. Please provide your new authorship list in the order you would like it to appear on the manuscript. Please indicate the corresponding author by adding (CA) behind the name. <u>If the Corresponding Author has changed, please indicate the reason under section 3</u>.

	First name(s)	Family name (this name will appear in full on the final publication and will be searchable in various abstract and indexing databases)	Affiliated institute	E-mail address
1st author	Ethan	Tietze		ethan.tietze@cuanschutz.edu
2nd author	Andre	Barbosa		andrecode20@gmail.com
3rd author	Bruno	Araujo		bhsa83@gmail.com
4th author	Veronica	Euclydes		veronicaeuclydes@alumni.usp.br
5th author	Bailey	Spiegelberg		
6th author	Hyeon Jin	Cho		hcho1239@umd.edu
7th author	Yong kyu	Lee		
8th author	Yanhong	Wang	Lieber Institute for Brain Development	Yanhong.Wang@libd.org
9th author	Alejandra	McCord	Lieber Institute for Brain Development	Alejandra.McCord@libd.org
10th author	Alan	Lorenzetti		

Please use an additional sheet if there are more than 10 authors.

Change of authorship request form - Journals (pre-acceptan[illegible]

Section 4 Continued: Proposed new authorship. Please provide your new authorship list in the order you would like it to appear on the manuscript. Please indicate the corresponding author by adding (CA) behind the name. <u>If the Corresponding Author has changed, please indicate the reason under section 3</u>.

	First name(s)	Family name (this name will appear in full on the final publication and will be searchable in various abstract and indexing databases)	Affiliated institute	E-mail address
11th author	Arthur	Feltrin		
12th author	Joyce	van de Leemput		jvandeleemput@gmail.com
13th author	Pasquale	Di Carlo		pasquale.dicarlo85@gmail.com
14th author	Gianluca	Ursini		
15th author	Kynon	Benjamin		
16th author	Helena	Brentani		
17th author	Joel	Kleinman		
18th author	Thomas	Hyde		
19th author	Daniel	Weinberger		
20th author	Ronald	McKay		
21th author	Joo Heon	Shin		
22th author	Tomoyo	Sawada (CA)		
23th author	Apua	Paquola		
24th author	Jennifer	Erwin (CA)		

Change of authorship request form - Journals (pre-acceptance)

Section 5: Author contribution, Acknowledgement and Disclosures. Please use this section to provide a new disclosure statement and, if appropriate, acknowledge any contributors who have been removed as authors and ensure you state what contribution any new authors made (if applicable per the journal or book (series) policy). Please ensure these are updated in your manuscript - <u>after approval of the change(s)</u> - as our production department will not transfer the information in this form to your manuscript.

New acknowledgements:

Supported by funding from the Lieber Institute for Brain Development and a NARSAD Young Investigator Grant from the Brain & Behavior Research Foundation to JAE, the National Institute of Health (NIH) T32 Fellowship (T32MH015330) to KJB. The authors are grateful for the financial support of the Coordenação de Aperfeiçoamento de Pessoal de Nível Superior (CAPES) - Financing code 001 (ARB, VE and AF). This work was also supported in part by FAPESP - Sao Paulo Research Foundation (http://www.fapesp.br/en/): Grant Number 2018/18560-6. ChatGPT was used to correct grammatical errors and improve text flow. We thank Bill Ulrich for the scRNA-seq browser. We thank Tricia Nilles and Worod Allak from the Becton Dickinson Immune Function Laboratory at the Johns Hopkins Bloomberg School of Public Health, for flow cytometry technical assistance. The facility was supported in part by CFAR: P30AI094189-04 (Chaisson).

New Disclosures (financial and non-financial interests, funding):

The authors declare no competing interests.

New Author Contributions statement (if applicable per the journal policy):

JAE and ET conceptualized the study; JAE, ET, ARB, BA and VE wrote the original draft; JAE, ET, ARB, BA, VE, HJC, YKL, YW, AM, AL, AF, JL, PDC, TS, and KJB performed experiments and data analysis; HB, JEK, TMH, BGS, AM, DRW, GU, RM, JHS, TS, ACMP and JE were responsible for writing, reviewing, and editing of the manuscript; JAE coordinated the study.

State 'Not applicable' if there are no new authors.

SPRINGER NATURE

Change of authorship request form - Journals (pre-acceptance)

Section 6: Declaration of agreement. *All* authors, unchanged, new and removed *must* sign this declaration.
(NB: Please print the form, (docu)-sign and return/upload a scanned copy. Please note that signatures that have been inserted as an image file are acceptable as long as it is handwritten. Typed names in the signature box are unacceptable.) * **Please delete as appropriate. Delete all of the bold if you were on the original authorship list and are remaining as an author.**

	First name	Family name		Signature	Date
1st author			I agree to the proposed new authorship shown in section 4 and the proposed change in corresponding author		
2nd author			I agree to the proposed new authorship shown in section 4 and the proposed change in corresponding author		
3rd author			I agree to the proposed new authorship shown in section 4 and the proposed change in corresponding author		
4th authors			I agree to the proposed new authorship shown in section 4 and the proposed change in corresponding author		
5th author			I agree to the proposed new authorship shown in section 4 and the proposed change in corresponding author		
6th author			I agree to the proposed new authorship shown in section 4 and the proposed change in corresponding author		
7th author			I agree to the proposed new authorship shown in section 4 and the proposed change in corresponding author		

SPRINGER NATURE

Change of authorship request form - Journals (pre-acceptance)

	First name	Family name		Signature	Date
8th author	Yanhong	Wang	I agree to the proposed new authorship shown in section 4 **and the addition of my name to the authorship list** and the proposed change in corresponding author	[illegible]	1/12/24
9th author	Alejandra	McCord	I agree to the proposed new authorship shown in section 4 **and the addition of my name to the authorship list** /and the proposed change in corresponding author	[illegible]	1/12/24
10th author			I agree to the proposed new authorship shown in section 4 and the proposed change in corresponding author		

Please use an additional sheet if there are more than 10 authors.

In case of author collaborations with formal agreement:

	Name of consortium/consortia	First name	Family name		Signature	Date
Representative/legal guarantor				I agree to the proposed new authorship shown in section 4 **/and the addition/removal*of my name to the authorship list** /and the proposed change in corresponding author		

Both added/removed authors should complete the information in the first table under Section 6.

---- End of form ----
